# DNA damage repair: historical perspectives, mechanistic pathways and clinical translation for targeted cancer therapy

**DOI:** 10.1038/s41392-021-00648-7

**Published:** 2021-07-09

**Authors:** Ruixue Huang, Ping-Kun Zhou

**Affiliations:** 1 Department of Occupational and Environmental Health, Xiangya School of Public Health, Central South University, Changsha, Hunan China; 2 Department of Radiation Biology, Beijing Key Laboratory for Radiobiology, Beijing Institute of Radiation Medicine, AMMS, Beijing, China

**Keywords:** Cancer genomics, Molecular medicine

## Abstract

Genomic instability is the hallmark of various cancers with the increasing accumulation of DNA damage. The application of radiotherapy and chemotherapy in cancer treatment is typically based on this property of cancers. However, the adverse effects including normal tissues injury are also accompanied by the radiotherapy and chemotherapy. Targeted cancer therapy has the potential to suppress cancer cells’ DNA damage response through tailoring therapy to cancer patients lacking specific DNA damage response functions. Obviously, understanding the broader role of DNA damage repair in cancers has became a basic and attractive strategy for targeted cancer therapy, in particular, raising novel hypothesis or theory in this field on the basis of previous scientists’ findings would be important for future promising druggable emerging targets. In this review, we first illustrate the timeline steps for the understanding the roles of DNA damage repair in the promotion of cancer and cancer therapy developed, then we summarize the mechanisms regarding DNA damage repair associated with targeted cancer therapy, highlighting the specific proteins behind targeting DNA damage repair that initiate functioning abnormally duo to extrinsic harm by environmental DNA damage factors, also, the DNA damage baseline drift leads to the harmful intrinsic targeted cancer therapy. In addition, clinical therapeutic drugs for DNA damage and repair including therapeutic effects, as well as the strategy and scheme of relative clinical trials were intensive discussed. Based on this background, we suggest two hypotheses, namely “environmental gear selection” to describe DNA damage repair pathway evolution, and “DNA damage baseline drift”, which may play a magnified role in mediating repair during cancer treatment. This two new hypothesis would shed new light on targeted cancer therapy, provide a much better or more comprehensive holistic view and also promote the development of new research direction and new overcoming strategies for patients.

## Introduction

### The Journey of DNA repair machinery system discovery

**Fig. 1:**
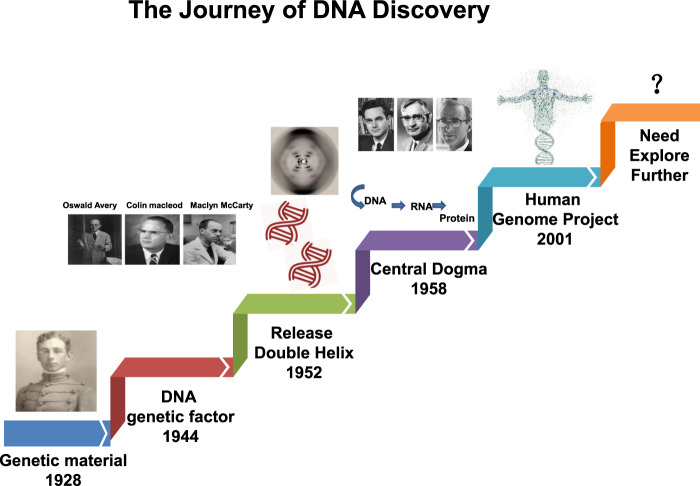
The journey of DNA discovery

In recent years, the availability of high-quality data on DNA from in vivo and in vitro research reported in the literature, as well as international conferences, funding, and collaboration among scientific communities has increased. In addition, new technologies related to translational research, targets for clinical therapy, and expertize among scientists have developed rapidly, indicating that DNA damage repair and genomic stability research has entered a new era after a century of steady progress. This field continuous to offer unprecedented opportunities for exploring further the secret of our genomic DNA structure integrity and function harmonization while also promoting clinical disease prevention and therapeutic options, especially boosting the precise cancer therapy. The secret veil of DNA was uncovered 70 years ago, since the famous “Photo 51”was published^1^ along with the ground breaking report entitled “Molecular Structure of Nucleic Acids: A Structure for Deoxyribose Nucleic Acid” by James Watson and Francis Crick^2^ in 1953. In this landmark study, DNA was illustrated as a double helix, resembling a ladder twisted along its length.. Over the following decades, many distinct biological topics such as DNA damage repair, genomic instability, cancer therapy and control of genetic diseases have been explored and found to be associated with DNA sequences and genomic profiles.^3–6^ In Fig. 1, we illustrate the brief history of the DNA and DNA damage repair discovery journey. Some important moments and scientists should be noted for their groundbreaking contributions to this journey. In 1927, the landmark discovery of gene mutation induced by X-ray was claimed by Gager and Blakeslee, Miller.^7^One year later, the genetic transformation of bacteria was reported by Frederick Griffith.^8^ In 1946, Hermann J. Muller was awarded the Nobel Prize in Physiology or Medicine for his contribution in discovery of genetic mutations in fruit flies and revealed that higher the dose of X-ray and other ionizing radiation exposed, the greater the number mutations that occurred.^9,10^ In 1944, Oswald Avery, Colin MacLeod and Maclyn McCarty provided robust evidence demonstrating that DNA was our genetic material.^11^ Shortly thereafter, Watson and Crick published the structure of DNA and announced that “we have discovered the secret of life” in 1953^2^. In 1964, the keyword “DNA repair” was formally introduced with the discovery of “Dark Repair” and photo reactivating “repair-replication” of UV light-induced *E. Coli* DNA injury by excision of damaged areas containing thymine dimers.^12–15^Since then, the DNA damage repair research rapidly spreads into the area of photobiology, radiobiology and cancer biology, etc. We understand presently that the term of “DNA repair” defines the biochemical and molecular biological processes of DNA damage removing and genomic integrity restoring, which including DNA damage sensing and signaling, repair machinery proteins recruited onto the damage sites, functioning and released step by step to restore the genomic integrity. The first evidence of the direct association between DNA repair deficiency and human disease and cancer predisposition was demonstrated in Xeroderma pigmentosum.^16,17^ In 2015, the Nobel Prize in Chemistry has been awarded to Tomas Lindahl, Paul Modrich and Aziz Sancar for their pioneering and fundamental contributions of mapping, at a molecular level, how cells repair damaged DNA and safeguard the genomic stability. Their work has provided fundamental knowledge of how a living cell functions and is, especially, used for the development of new cancer treatments.

Now, it is well known that mammalian cells have evolved multiple and diverse machineries for repairing every type of spontaneously occurring as well as exogenous factors-induced DNA damage.

Following these critical discoveries and foundational works, the Human Genome Project was initiated in 2001, which provided a deep understanding of the evolution of the human population as well as relationships between human health and diseases.^18,19^ Other projects exploring the topics of genome organization and function, such as the Encyclopedia of DNA Elements(ENCODE),have provided significant conclusions based on extensive sequencing results.^20,21^ Over the past century, understanding of the DNA double helixintegrityled to extremely significant advances in our appreciation of biological processes including gene transcription, replication and protein expression. Notably, clinical medicine has become more broadly based on genetics and DNA function,^22,23^ the increasing knowledge of DNA repair opened up a new irreplaceable path of precisely targeted cancer therapy. Over the next century, many more promising advancements will undoubtedly occur in DNA-related research. Despite these significant past discoveries, the underlying molecular mechanisms and functions of DNA have remained unclear in recent decades. Clinical applications for disease therapy necessarily lag behind research into DNA damage repair-based medical diagnosis and therapeutic targets. Nevertheless, DNA damage repair research is leading to better appreciation of the complexity and diversity of diseases. Future efforts are needed to elucidate further the secrets of life using DNA and to introduce novel concepts and hypotheses as powerful and compelling as the discovery of the DNA double helix and DNA repair-based genomic integrity maintaining mechanisms.^22–25^

### A historical perspective of linking the genomic distortion with cancer

The origin of cancer medicine is associated with a clinical discovery based on medical record analysis and an epidemiological survey. Percival Pott, known as the father of epidemiology, observed a high prevalence of scrotal cancer among the boys who were employed as chimney sweeps, and attributed the cancer to soot exposure.^26^ This was the first evidence of occupational exposure to hazard factors associated with cancer development. Prior to the discovery of the structure of DNA, Dr. Theodor Boveri proposed in 1914 the remarkable theory that the origin of malignant tumors was from cancer cells, and that cancer cells formed through alteration of normal cells.^27^ He expounded on this theory by suggesting that tiny microscopic bodies called chromosomes might be abnormally distributed in tumor cells.^28,29^ In the late 1920s, Hermann Muller, the principal discoverer of gene mutations mentioned above, reported that exposing *Drosophila melanogaster* to ionizing radiation from X-rays could result in the “transmutation” of a gene, contributing to aberration of the chromosome.^9,10^ In the 1930s, it was observed that, compared to the normal human cells with 46 chromosomes, the number of chromosomes in cancer cells typically varies and frequently exceeds 46.^30,31^ Meanwhile, scientists noted that cancer cells have more rapid and stronger growth ability than normal cells.^32–34^ By the 1950s, shortly after the DNA structure was described, it was shown that exposure to chemical mutagens such as the chemical benzene could produce chromosome breakage and increase DNA mutation rates.^35,36^ In the 1980s, the process of carcinogenesis was described, with necessary conditions of DNA mutations generated due to environmental mutagen insult and the occurrence of DNA damage without effective repair.^37,38^ Thus, the critical role of DNA damage response(DDR) was determined. In the following years, extensive evidence obtained using many new methods developed from the study of DDR processes indicated that DNA repair,^39^ DNA damage signaling and repair pathways,^40,41^ cell cycle checkpoints,^42,43^ apoptosis,^44–46^ fidelity of replication,^47,48^ DNA re-replication^49^ and telomeres^50,51^are all closely associated with cancer.^51^ Based on these studies of the DNA molecule integrity and the process of genetic mutation, the linkage between DNA mutation and cell carcinogenesis became increasingly clear. Phil Lawley, a pioneering researcher of DNA damage and carcinogenesis, found that some alkylating agents, such as butadiene dioxide,^52–54^ could interact with DNA, forming harmful adducts and eventually disrupting the normal role of DNA as a molecule template.^55^ The hypothesis that certain cancer genes are susceptible to such agents was proposed and extensively studied over the past few decades. Since then, chemotherapeutic agents and radiotherapy have been found to treat various cancers effectively through DNA damage induction. In the war against cancer, numerous agents have been developed and novel technical strategies have also been explored. However, many challenges and unsolved issues remain that require further study, such as: (i) the detailed molecular mechanisms underlying the cancer cell DNA response to chemotherapeutic agents and radiotherapy; (ii) how cancer cells become resistant to chemotherapeutic agents and radiotherapy; (iii) possible new and promising biomarkers for investigation as novel inhibitors or therapy agents; and (iv) most importantly, the basic biological mechanisms underlying the DDR. With such information, effective cancer therapies could be developed to target DDR and ultimately prevent or cure cancer.

### DNA damage, cellular response, repair and cancer

#### Genome stability

To support survival and reproduction, maintaining genome stability is a critical priority of all cells.^56^ Any abnormal alterations of the genetic base sequence can disrupt cellular biological processes, hampering cellular functions and possibly inducing carcinogenesis or even cell death.^57^ Specifically, strong evidence has indicated that genomic instability promotes cancer pathogenesis through a cascade response involving a series of proto-oncogenes that are continuously triggered or anti-oncogenes that are suppressed.^58–60^ In this context, the *EGFR*(epidermal growth factor receptor), *MYC* and *RAS* families have been commonly recognized as proto-oncogenes,^61^ whereas *TP*53 is a well known tumor suppressor gene.^62,63^ Accordingly, to reduce the possibility of genetic dysregulation of genome stability, cells have evolved a range of genome stability-related signal pathways and post-translational modifications,^64^ which assess the accuracy of DNA metabolism and prevent accumulation of DNA damage.^65^ For example, multiple families including ATM (ATM serine/protein kinase), ATR (ATR serine/threonine kinase), and DNA-PKcs (DNA-dependent protein kinase catalytic subunit) can initiate the signaling cascade in mammalian cells.^66^ A recent review by Monique PCM et al. summarized the advances of ubiquitination research and noted that ubiquitination performs vital roles in regulating cellular homeostasis through numerous enzymes^67^ and proteins. The complex functions of this compound have become known as the “ubiquitin code” in the scientific community.^56^ Genomic instability is a common characteristic of most cancer cells.^68^ For example, a high ratio of chromosomal instability is associated with mitotic spindle checkpoint deficiency in most breast cancer cell lines.^69^ The molecular mechanisms through which cells maintain genome stability and the repercussions of genomic instability are essential emerging issues relevant to clinical cancer avoidance.

Accumulating evidence has shown that a DNA double-strand break(DSB) is typically the most harmful type of DNA damage, and that it compromises genome stability.^70^ In mammalian cells, a number of vital DNA repair functions and processes against various DNA damage have evolved. For example, the mismatch repair pathway, base excision repair pathway and nucleotide excision pathways have been well characterized.^71^ However, cancer cells have frequently evolved in relation to abnormal DNA damage repair functions and processes. For example, in many cancer cell lines, such as mantle cell lymphoma(MCL), ATM is recurrently mutated in around thirty to almost fifty percent of cases.^72^ These mutations may be linked with cancer chemotherapy resistance.^73^ Furthermore, cell cycle machinery-related genes play critical roles in driving avoidance of chemotherapy and radiotherapy treatment effects by cancer cells.^74^ Most measures developed to kill cancer cells involve: (i) stimulating G1 phase aberrant homologous recombination in cancer cells; (ii) inducing mitotic catastrophe in cancer cells; or (iii) deleting the cell cycle checkpoint.^70,75,76^ Despite data showing that genomic instability may be associated with ROS(reactive oxygen species),^77,78^ in this review, we focus on DNA damage repair, as it is a major clinical target of cancer chemotherapy and radiotherapy.

### DNA damage

#### DNA damage and cancer

It is critical for maintaining genomic DNA stability due to its role as the template for replication and transcription.^79^ As described above, damage to DNA from environmental hazards insult as well as endogenous toxic agents such as free radicals can compromise genome stability and cause or promote many diseases, particularly cancer.^37,80,81^ As the DNA molecule is the basic genetic material, it is vital for ensuring the integrity of DNA structure and function to support normal life activities and stable species characteristics.^82,83^ Indeed, when experiencing either endogenous or exogenous stresses, cells can generate various types of DNA damage, including base pair alterations, DNA replication errors^84^ and distortion and breakage of the DNA double helix strands.^85^ Common exogenous factors, especially certain environmental hazards such as toxic heavy metals and ionizing radiation, have been intensively studied and found to cause serious DNA damage.^86–90^ Endogenous materials are often released during the metabolism of exogenous materials in the body or after cell damage and the loss of cell membrane integrity.^91^ DNA damage can occur through two pathways, namely direct effects and indirect effects. In the direct pathway, endogenous or exogenous materials directly contact DNA, leading to the breakage of chemical bonds in DNA molecules, and thereby changing the structure and activity of DNA.^92,93^ In the review by Anthony T et al., endogenous stresses including gene transcription and replication in cancer cells are noted to cause genomic instability.^79^ In the indirect pathway, endogenous or exogenous materials activate products such as free radicals^94,95^ that can damage DNA.^96^

**Fig. 2:**
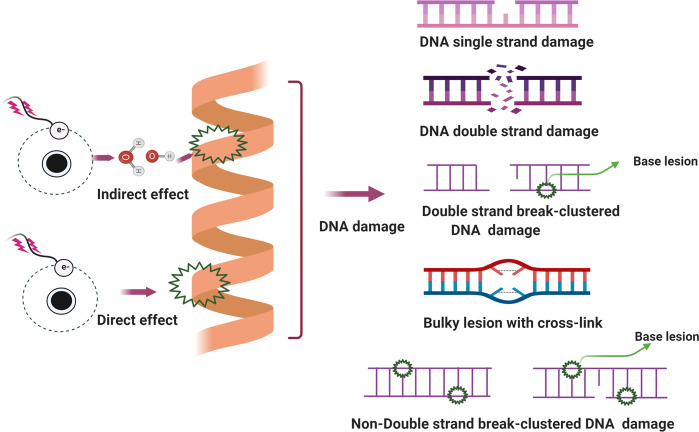
Main types of DNA damage along with differential definitions of double-strand breakage-based and non-double-strand breakage-based clustered DNA damage

Several types of DNA damage have been reported previously, as follows: (i) single-strand breaks; (ii) double-strand breaks (DSBs);^97^ (iii) base damage; (iv) sugar damage; (iv) DNA cross-linking and (v) clustered damaged sites,^98^ of which the most deleterious lesion and the most severe threat to cells is the DSB. DSBs that occur without effective repair or error-prone repair can cause carcinogenesis or cell death.^99^ Lindahl et al. reported that, each day, our cells may be subject to around 70,000 instances of DNA damage.^100^ Most of these lesions are single-strand breaks, and only a few are DSBs, which are less frequent. Numerous studies have illustrated that DNA subject to oxidative stress exhibits a large number of base and sugar lesions,^101^ such as guanine modification or 7,8-dihydro-8-oxo-2′-deoxyguanosine (8-OH-dG). Base lesions are usually caused indirectly by ROS generated due to oxidative stresses such as radiolysis of water molecule induced by ionizing radiation.^102^ Sugar damage, such as 8,5′-cyclopurine-2′-deoxynucleosides, can be caused by free radical insult to the sugar moiety.^103,104^ DNA cross-linking is often attributed to exposure to chemical cross-linking agents, e.g. cis-platinum, or free radical-generating ionizing radiation.^105^ With this type of damage, DNA repair-related proteins are trapped with DNA, causing the proteins to adhere to the 5′ or 3′ end of the DNA strand break.^106^ Furthermore, DNA cross-linking can hinder the activities of some vital enzymes such as DNA helicases and polymerases.^107,108^ A review described the formation of DNA cross-links due to exposure to various endogenous, environmental and chemotherapeutic agents.^106^ However, elucidation of how this process is regulated and its full biological functions in mammalian cells and cancer cells require further research. Clustered DNA damage, sometimes described as multiple local damage sites, refers to damage in which at least 20 base pairs are separated.^109^ Clustered DNA damage usually consists of multiple lesions such as base damage, a basic site damage and single-strand breakage.^110^ However, in contrast to DSBs, the multiple lesions of clustered DNA damage may be present on the same DNA strand or on opposing strands within a tiny range. Figure 2 illustrates the main types of DNA damage along with differential definitions of double-strand breakage-based and non-double-strand breakage-based clustered DNA damage. In general, clustered DNA damage results in enhanced mutation frequency,^111^ cancer, and cell death. The mechanism of clustered damage has been described as a base obtaining a single electron, after which multiple electron pathways are activated.^111^ However, whether a beneficial result (friend) or non-beneficial result (foe) is obtained from clustered DNA damage in cancer cells or normal cells requires further study.^112^ As cells face a tremendous amount of DNA damage arising from various exogenous and endogenous stressors, such as ionizing radiation or ROS,^112,113^ recognition of how DNA damage occurs requires deeper investigation. Many scientific issues remain to be addressed in future research, such as: (i) excluding the currently known DNA damage types, other novel DNA damage styles may exist that have not yet been discovered; (ii) methods to evaluate and measure DNA damage types and degrees, or visualization techniques for DNA damage; (iii) monitoring processes for DNA damage and identification of effective biomarkers for early detection of DNA damage; and (iv) obtaining reference values for the exogenous and endogenous stressors that drive DNA damage. Investigating these issues may help to standardize DNA damage caused by various insults. Importantly, innovative technologies and unique theoretical models would be developed while exploring these interesting issues.

### DNA damage response

#### Sensors in the DNA damage response

To avoid DNA damage, cells have evolved numerous interacting mechanisms for ensuring genomic stability or have even used DNA damage to produce new opportunities for natural selection.^114,115^ These mechanisms have been identified as the DNA damage response (DDR). Generally, DDR mechanisms involve feedback signals from damage sites and movement of repair factors to cluster at damage sites. In our previous published review, we used ionizing radiation to explore how the cell’s DNA damage sensors and signaling transducers interact in the DDR. We focused on the critical issue of recognizing and identifying DNA damage signals to activate the subsequent biological response cascade.^6^ Therefore, in this review, we focus on the association between DDR and cancer. Due to the characteristic genomic instability of cancer cells, mutations and tumor heterogeneity are common and widespread.^116^ These features suggest that cancer cells are prone to enhanced proliferation, growth and tumorigenes is due to dysregulation of DDR-related mechanisms.^117^ The acquisition of specific mutations in cancer cells might, in turn, increase the dependence on other DDR factors for survival.^118^ The development of cancer requires both mutagenic and non-mutagenic events. Cells exposed to endogenous and exogenous factors that act as mutagenic agents show impacts throughout the process of cell oncogenesis, but these effects are stronger in cancer cells with mutated or deficient DDR genes.^119,120^ Alteration of DDR genes has been demonstrated in various cancers, including breast cancer and prostate cancer.^40,118^ For example, BRCA1 or BRCA2 inactivating mutations were found in ninety out of almost six hundred breast cancer patients.^121^ Moreover, DNA mismatch repair-dependent DDR pathways, such as loss of non-canonical mismatch repair gene functions, contribute to improved treatment outcomes of colorectal cancer.^122^

Here, as a few DNA damage sensors such as γH2AX, Mre11-RAD50-NBS1 complex, Ku70/Ku80, MDC1 and 53BP1 can initiate the damage signaling thus trigger the DDR,^123^ In a study,γH2AX may be expressed not only to detect genetic effects caused by various toxic substances but also to monitor the clinical efficacy of chemotherapy and radiotherapy and the sensitivity alternations of cancer cells to anticancer agents.^124^ Another study assessed DDR processes after hepatocellular cancer therapy and found that γH2AX expression increased.^125^ Screening for H2AX variant functions and targeting of H2AX have been proposed as cancer treatments.^126^ Ku70/Ku80 expression also exhibited a significant increase in rectal cancer patients after chemotherapy and radiotherapy treatment, and further study showed that the increase in Ku70/Ku80 expression was correlated with chemo- and radio-resistance in various cancers.^127^ Ku70/Ku80 expression can be used as a molecular cluster for predicting the susceptibility of rectal cancer to chemoradiotherapy.^128^ In contrast to oxygen sensors, which have been extensively investigated, these DDR sensors are still in the early stages of molecular characterization, and their roles in sensing DNA damage and signaling, cancer progression and therapy require further study.

#### DNA damage response and cancer

**Fig. 3:**
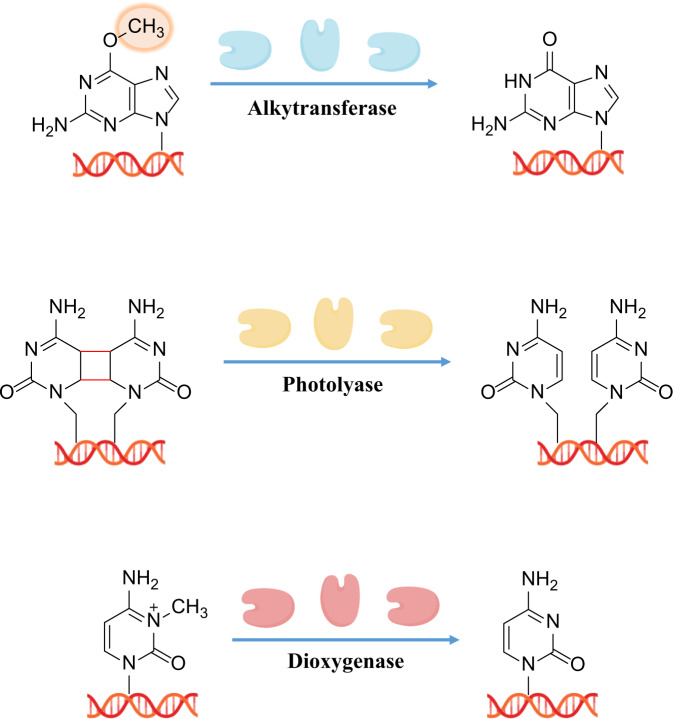
Timeline of DDR-related findings and concepts related to cancer, highlighting the scientists who worked to provide a deeper understanding of the roles of DDR in cancer

A review literature highlighted the various concepts behind targeting of DDR in cancer,^129^ which were summarized that (i) DDR can be used as a target of anticancer drug treatments; (ii) as most cancer cells have a deficiency of some DDR pathways’ ability, inhibition targets can be explored in the remaining pathways; (iii) DDR inhibition can be used to investigate cancer replication stress; and finally (iv) the author considered using DDR inhibitors in specific DDR-lacking backgrounds initially to promote exploration of DDR-based agents for cancer treatment in the future. The first description on the association of cancer with occupational exposures was presented in 1775 by the British surgeon Percivall Pott,^130^ who first showed the link between the occupational exposure of chimney sweeps and scrotal cancer. In 1946, the X-ray induced recessive lethal of *Drosophila* was first reported to be related to the chromosomal breakage.^131,132^ Later, the discovery of the helical structure of DNA led to the introduction of the concept of DNA as the hereditary material.^133,134^ Soon thereafter, repair of X-ray damage to DNA was reported in bacteria in 1966^135^ and in eucaryotic cell in 1967.^136^ Significant research showed that defective DNA repair resulted in many diseases and, in particular, cancer propensity, during the period from 1969 to 2015.^137,138^ In 1972, apoptosis was defined, which is a programmed cell death pathway occurring in cells during the normal tissue development or encountering exogenous stresses, especially DNA damage.^139,140^ In 1981, the concept of oncogenes was introduced,^141^ followed by the concept of tumor suppressors three years later.^142^ In 1989, cell cycle checkpoints were proposed and in 1990, p53 was reported to be mutated in various cancers.^143^ Then, in 1997, caretaker and gatekeeper genes were proposed based on the research discoveries on DNA repair genes *BRCA*1/2, *RAD*51.^144–146^ In 2002, ROS production and DNA damage attributable to deregulated metabolism induced by oncogene expression were reported.^147,148^ In 2005, DDR was described as an anticancer barrier in early-stage tumorigenesis, but the genes showing DDR mutations were absent from later-stage tumors.^149^ To date, a large number of DDR genes have been identified in various cancers.^150,151^ Figure 3 illustrates the timeline of DDR-related findings and concepts related to cancer, highlighting the scientists who worked to provide a deeper understanding of the roles of DDR in cancer.

Alongside the DDR processes described above, including cell cycle checkpoints and apoptosis, we present DDR signaling by way of a brief introduction to how DDR pathways can affect cancer development. First, a healthy cell affected by environmental hazards, viral or bacterial infection, or ROS may initiate DNA damage and mutations, increasing oncogene activation, tumor suppressor inactivation and replicative and oxidative stresses. The oncogene activation might occur directly or indirectly. As a result, DNA replication fork fidelity and replication recovery are compromised.^152–154^ Hence, increased DNA damage and mutations in normal cells would hamper genomic stability. At this stage, the damaged cells still exhibit a range of responses, including activation of checkpoint arrest and triggering of increased p53 expression to protect cells against further damage. However, downregulation of DDR processes should disturb the proliferation of pre-cancerous cells.^155–157^ General DDR pathway research in relation to cancer development provides important information that may be useful for the design of targeted cancer therapies.^158,159^ More importantly, understanding DDR can also help elucidate why targeted clinical therapy strategies often fail.^160,161^ Debjani P et al. assessed the performance of cancer cells escaping targeted lung cancer therapy, found that the key event was activation of the TGF-β signaling pathway in some cancer cells after targeted therapy.^162^ TGF-β activation can inhibit the expression of DDR-related genes, resulting in decreased DNA repair ability and, thus, accumulation of mutations. Other advances have shown that tumor heterogeneity may influence the outcomes of targeted cancer therapy.^163,164^ For example, some scientists have reported that ALK-targeted therapy differs among cancer patients, with many patients treated with ALK-targeted therapy developing therapy resistance, which results in cancer progression.^165^ Targeted therapies against other cancers, such as non-small cell lung cancer (NSCLS), also face challenges related to tumor heterogeneity, which impact acquired and inherent drug resistance.^166^

New technologies, such as cancer genome profiling using deep sequencing and microarrays and single-cell sequencing offer more information about which DDR-related genes are mutated or mis-regulated.^62,167–170^ However, a better understanding of DDR pathways and discovery of new and valuable ideas for improving cancer treatment are still urgently needed.

### DNA damage repair

#### A historical perspective of DNA damage repair

Many human pathologies such as tumors and chronic metabolic diseases can be clearly attributed to DNA damage induction.^171^ Indeed, although DNA damage is common and its occurrence is very frequent, such damage must be repaired immediately and correctly to ensure the exact transfer of genetic information during cell division.^172,173^ Without appropriate DDR capacity after insult from environmental or endogenous stressors, negative effects may occur in normal cells, as follows: (i) increasing opportunities for genomic defects; (ii) possible genomic instability and malignant transformation; (iii) enhanced development of cancer; and (iv) further injury to cellular DNA repair ability, as DNA damage signaling and inappropriate repair processing in cancer cells would benefit cancer cell growth and proliferation while disrupting the outcomes of cancer chemo-and radiotherapy. Over the long period of around 4 billion years of evolution, it is unsurprising that cells have prioritized minimizing mutagenesis and protecting genomic replication through effective and quick repair of DNA damage.^174^ In recent years, numerous studies have reported evidence of the importance of DNA damage repair: (i) a few types of DNA damage have been illustrated over recent decades, of which DNA double-strand breaks (DSBs) represent the greatest risk for causing genomic instability;^173^ (ii) some components of DNA repair pathways are so important that life would not be sustained without them, such as ATR, which is critical for early embryonic development and its deficiency results in chromosomal fragmentation and early embryonic lethality;^175^ (iii) many hereditary disorders have been attributed to DNA repair deficiencies, such as the observation by Jim Cleaver that patients with the rare autosomal-recessive cancer predisposition syndrome, xeroderma pigmentosum, lacked the ability to perform DNA repair after damage due to UV exposure;^138^ and (iv) defective DNA repair pathways are associated with cancer initiation, as shown by a study in the 1990s, which found Lynch syndrome was related to mutation of the DNA repair proteins MutS and MutL.^176^

From a historical perspective, early research into DDR focused on observations, as described above.^177^ In 1940, American biologist Albert Kelner identified photo reactivation, which is an enzyme-catalyzed reaction, as the enlightenment DNA repair mechanism.^178^ Then, in 1964, Setlow RB and Carrier WL reported an error-correcting mechanism in which intrastrand thymine dimers formed after UV radiation of DNA accounted for a large fraction of the observed biological damage to DNA.^15,179^ In the same year, the term “DNA repair” was formally founded with the discovery of “Dark repair” and “Repair-replication” or “unscheduled DNA synthesis” of ultraviolet injury to the DNA in *Escherichia coli*.^12,13,180^ These studies showed that one strand of damaged DNA could be excised, and the resulting gap could be repaired, using the intact complementary strand as a template.^12,181^ This repair pathway is known as nucleotide excision repair. In 1968, J R Cleaver et al. validated it as a repair replication mechanism through observation of UV-induced lesions to HeLa cell DNA.^182^ Soon thereafter, polynucleotide ligase activity was discovered in the cell-free extracts from *E. Coli* by Gellert M in a study showing that *E. coli* extracts could convert lambda DNA to covalent circles.^183^ In other words, polynucleotide ligase can combine a newly synthesized patch with the contiguous parental DNA strand.

In the mid-1970s, the excision repair processes of base excision repair and mismatch repair were described. Lindahl revealed that an N-glycosidase was active in DNA repair based on its ability to deaminate dCMP residues into an easily repairable form.^184^ Meanwhile, Wagner Jr. and Meselson used *E.coli* to identify repair tracts originating at mismatches. The repair process propagatesin the direction of 5′ to 3′ and can cover approximately three thousand nucleotides.^185^ In terms of excision repair, the optimal outcome is that the DNA can be replicated normally after excision and repair, but it is also possible for an advancing replication fork to encounter the damage site after excision and prior to the completion of repair, which is known as synthetic death.^174^ Compared to the former repair type, the latter is more complicated and more likely to be lethal. In 1975, an interesting hypothesis was raised by Radman, who suggested that *E. coli* possesses an inducible DNA repair system, called “SOS repair”.^186^ The main components of this hypothesis were: (i) DNA damage initiated the “SOS repair” process; (ii) de novo protein synthesis is involved in the repair process; and (iii) physiological and genetic status requirements must be met for further prophage induction.^186^ This hypothesis was confirmed through many later experiments, and some new concepts have been incorporated into this model. For example, a study showed that cells treated with rifampin to eliminate their ROS repair ability exhibited reduced repair efficacy of global cyclobutane pyrimidine dimer (CPD) formation due to UV radiation exposure.^187^ These findings provided the insights into the enzymes responsible for DNA damage detection, and showed that they may attack undamaged DNA, with deleterious consequences. In other words, these sensitive DNA repair enzymes perform dual roles depending on their concentrations. At low concentration, these enzymes are kept in check until needed for repairing specific DNA damage. Since these studies, the concepts of transcription-coupled repair sub-pathway and global genome repair sub-pathway of nucleotide excision repair (NER) have been supported by numerous studies.^188,189^ The DNA damage in the active transcription gene or the transcribing strand is preferentially repair through the transcription-coupled repair sub-pathway.^188,190^ Global genome repair is another sub-pathway of nucleotide excision repair, through which the DNA damage in whole genome is repaired with equal efficiency. The mechanistic difference between transcription-coupled repair and global genome repair is mainly that, in the former process, the stalling of RNA polymerase at transcriptionally active genes favors the recruitment of Cockayne syndrome proteins A and B, whereas in the latter process, helix-distorting damage is recognized by XPC and its partners RAD23B (Rad 23 homolog B) and CETN2 (centrin 2).^191^ However, compared with global genome repair, transcription-coupled repair is more constitutive and is evidently not inducible as a DDR response. Excluding excision repair, several other repair pathways that support improved replication to overcome the obstruction of replication caused by lesions without their removal have been reported; they are known as tolerance pathways. These pathways require the function of specialized DNA polymerases.^93,192,193^ At this point, a “collapsed replication fork” had been defined and its role in the loss of DNA synthesis capacity was known.^194^ In this process, the fork collapse contributes to genomic instability or even death.^194,195^ In general, after reviewing DNA damage repair from a historical perspective,^196^ concerns remain, which can be summarized as follows: (i) Is the previous DNA damage repair definition sufficient to represent fully the process and its significance? Based on the rapid development of DNA damage research and a deeper appreciation of DNA damage repair, the definition should be expanded to include exogenous and endogenous insults, genomic early and later responses, DNA repair-related enzymes, and early events associated with later outcomes. (ii) Most of previous researches aimed to uncover new targeted proteins and enzymes rather than considering the interactions among multiple DNA repair pathways. Sometimes, various DNA repair pathways can handle the same damage sites in competing ways, but how this interaction occurs remains unclear. Moreover, the processes that occur at each step of multiple repair routines require further investigation. (iii) The threshold level is an essential concern for initiation of DNA damage repair, but leads to low-level lesions often being overlooked. However, multiple long-term low-level lesions may lead to DNA repair via some novel pathway or mechanism, which requires further validation and testing. (iv) Finally, basic information about DNA repair is lacking, including how damage to bases and other structures of DNA is sensed in cells, what roles the sensing machinery plays in the cellular response to DNA damage, and how the cells perform cell cycle arrest in response to DNA damage in normal cells and cancer cells. Furthermore, in the context of cell mutagenesis or lethality, more information is needed about how DNA damage repair-related enzymes and proteins regulate downstream events in combination with other factors after the recognition of an aberration. These questions are very basic, but have yet to be answered fully and clearly. In addition, it is important to learn from previous research and apply these discoveries in the clinic setting in the future. The greatest value of DDR research is that a deeper understanding of the secrets of life will allow us to face the challenges that arise from environmental, social and technological issues more effectively.

#### DNA damage repair pathways

Several repair pathways exist, including direct reversal, base excision repair, nucleotide excision repair, mismatch repair, single-strand break repair and DSB repair.^197–199^ Direct repair generally refers to the repair of pyrimidine dimers formed due to UV exposure or other factors or the repair of alkylated bases. Nucleotide excision repair refers to repair of DNA replication lesions or bulky adducts arising from distortions of the DNA structure.^3,200^ Mismatch repair refers to adjustment of mismatched base pairs in double-stranded DNA, as well as repair of some insertions or deletions of less than 4 nt.^201,202^ Double-strand break repair refers to repair of DSB lesions.^203–205^

### Direct reversal repair

As described above, induced DNA damage often refers to damage caused by alkylation, oxidation, UV and cross-linking agents.^206^ Direct reversal of the base lesion rather than excision is the one simplest step error-free and most economical DNA repair mechanism to have evolved.^206–208^Cells have also developed direct reversal mechanisms for several types of DNA damage, such as alkylation, inter/intra-strand cross-link. In *E. coli*, the mechanism of the direct DNA damage reversal reaction was described as a “flip-out”process.^209^ Briefly, enzymes first form a long loop, then DNA photolyase binds to duplex DNA, goes through a series of energy transfer, single electron transfer and enzymatic catalysis steps, and forms a flip-out helix structure to skip the break site for direct reversal.^209–211^ This form of direct DNA damage reversal is considered to be beneficial to cells, as it is a highly effective and simple method to address an important and necessary issue.

Unlike other molecules, which can be replaced, DNA cannot be replaced after being damaged, and must instead be repaired. Three classical DNA damage direct reversal mechanisms have been described, namely, repair of O-alkylated DNA damage by alkyltransferases and dioxygenases, repair of photolesions caused by ultraviolet (UV) radiation through the work of spore photoproduct lyases and photolyases,^212,213^ and reversal of N-alkylated base adducts by AlkB family dioxygenases.^214^ DNA is constantly subjected to numerous environmental insults. Among such hazards, alkylating chemicals, which are often applied as cancer chemotherapy agents, can cause DNA damage in the form of alkylation.^215,216^ Endogenous products, including metabolites such as adenosylmethionine arising from many biological processes, may also damage DNA.^217,218^ After damage from these agents, some typical damage response molecules such as methylguanine and methyladenine are formed on double-stranded DNA.^219,220^ These materials can increase the cell’s mutagenic and carcinogenic potentials, for example, by increasing the chance of base misincorporation.^221^ Alkyltransferases are associated with direct removal of DNA alkylation damage. Studies have shown that this enzyme is responsible for the removal of mutagenic alkyl adducts on the bases of the O6 atom of 2′-deoxyguanosine and the O4 atom of thymidine.^222,223^ In cells, the importance of O6-alkylguanine DNA alkyltransferase-based direct reversal is greater than that of nucleotide excision repair or base excision repair, suggesting the critical role of alkyltransferases.^224^ The potential role of this protein in DNA repair was reported 40 years ago. Its main roles include prevention of mutations, cytotoxicity, and cancer development.^225^ Alkyltransferases have been identified in many living things.^226^ However, whether other co-factors or energy resources interact with alkyltransferases to perform direct DNA damage reversal remains unknown.^225^ Meanwhile, although multitudinous studies have been conducted to investigate the activities of alkyltransferases, how their polymorphisms relate to health, and specifically cancer therapy, remain unclear at present.

Photolyases, which are 50–55kD single-chain flavoproteins, are damage-specific binding proteins active in the response to the formation of UV-induced cyclobutane pyrimidine dimers (CPDs)^227,228^ and 6–4 photoproducts.^229^ From the perspective of LO Essen et al., photolyases are highly effective light-driven DNA repair enzymes, which function specifically in the reversal of genomic lesions induced by UV radiation.^230^ The mechanism of photolyase-related reversal is generally similar to that following induction of DNA lesions by UV insult; specifically, an electron is injected at the lesion site, activating cleavage of cyclobutane-pyrimidine dimers or 6–4 photoproducts inside the duplex DNA structure.^230^ This reversal method is highly effective and simple, as an electron is shuttled to the lesion site for direct destabilization.^231^ The energy to drive this reversal reaction comes from chromophores excited through intake of a photon.^232–234^ With the advancement of this research field, other mechanisms, including an exclusive bifurcating-electron-transfer method with a cyclic radical mechanism, have been continuously reported. For dimer repair, six steps have been identified, typically including three electron transferences and two bindings to lesions.^229^ Through dynamic analysis, new discoveries such as a unique electron-tunneling pathway and essential residues at repair trigger sites have been revealed.^229,235,236^ Importantly, recent crystal structure determination of photolyases has provided new direct insights into the relationship between photolyase structure and its roles in DNA damage repair,^237^ supporting further comparison between DNA photolyases and spore photoproduct lyases.^237–239^

**Fig. 4:**
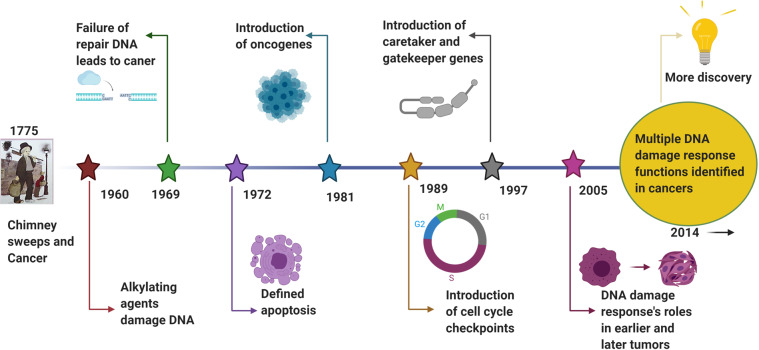
Three types of direct DNA damage reversal including representative substrates, repair proteins, cofactors, and the corresponding repair products

In recent years, the AlkB family of demethylases has attracted increasing attention for its regulatory role in oxidative DNA repair. In 2002, studies by Pal F et al.^240^and Sarah CT et al.^241^ revealed a third type of direct reversal mechanism for DNA damage, reporting that AlkB protein in *E. coli* can repair cytotoxic damage due to 1-methyladenine and 3-methylcytosine in DNA, and that this reaction by AlkB is dependent on oxygen and α-ketoglutarate. Figure 4 lists the three types of direct DNA damage reversal described above, including representative substrates, repair proteins, cofactors, and the corresponding repair products.

### Base excision repair

Exposure to ionizing radiation produces radicals.^242,243^ Radicals may cause base lesions and thus, base excision repair may be initiated to address these lesions.^244,245^ Key enzymes such as OGG1 can sense damaged bases and are responsible for the recognition and removal of 8-oxoguanine.^246,247^ The result of base excision repair is that 1 to 10 nucleotides, but no more, can be replaced by short to long patches.^248^ The cell’s status and background, lesion style, and levels of exogenous and endogenous materials affect the selection between short patch and long patch base excision repair.^249–251^ The importance of base excision repair is not only for ensuring genomic stability but also that its dysregulation would lead to increased risks of cancer, aging-related diseases and other serious disorders.^252^ However, base excision repair does not simply serve as an isolated repair pathway, it is one component of the larger DNA damage repair machinery. It forms a network in combination with other pathways and may in turn be regulated by other pathways via a feedback mechanism.^253^

### Nucleotide excision repair

Compared to base excision repair, nucleotide excision repair is more complex, as it is responsible for addressing complicated lesions including bulky adducts and cross-linking lesions,^254,255^ and there are two sub-pathways of TCR-NER and global genome repair (GGR)-NER as mentioned above. The source of this type of DNA damage is alkylating or cross-linking agents. Deficiency of nucleotide excision repair is typically associated with several human diseases, including xeroderma pigmentosum and other neurological diseases.^200^ The repair process has been elucidated through research over previous decades. In TCR-NER, the hampered RNA polymerases by the damage constitutes the initial step for recognizing DNA lesions, then recruits the CSB/ERCC6, which in turn recruits CSA/ERCC8 complex. In GGR-NER, the protein complex XPC/RAD23B/CETN2 can sense and recognize DNA distortion and recruit helicase TFIIH to form a XPC-RAD23B-TFIIH complex to unwind the DNA helix. Once the pre-incision complex is ready, the endonuclease XPG and XPF/ERCC1 are recruited, which can cut the strand at the 3′ and 5′ flanks of damage site, respectively, to ensure that of a piece of damage-containing nucleotides are removed. Then, the proteins responsible for synthesizing the missing nucleotides are recruited and, finally, DNA ligase fills the gap to complete the repair process.^256–258^

### Mismatch repair

The primary purpose of mismatch repair is to counteract replication errors and thus improve the fidelity of replication.^259^ This repair pathway is mainly used to resolve single nucleotide mismatches and small insertion loops generated by DNA polymerase.^119,260^ The mismatch repair pathway consists of three steps. First, protein complexes such as the MSH2-MSH6 heteroduplex sense and recognize the mismatch and identify the site of the insertion-deletion loop.^261,262^ These protein complexes will quickly move to the mismatch lesion site and bind to the DNA molecule to form a sliding clamp. At this point, many proteins gather to perform various functions. For example, exonuclease 1 (EXO1) has been reported to carry out excision of nucleotides in the 5′- > 3′ direction.^263^ Another protein, replication protein A (RPA) serves as a binding function of single-strand DNA produced by EXO1 to prevent further DNA degradation.^264^ MLH1, a subunit of MutLα, whose defect is responsible for ~50% of MMR defected cancers, may restrained DNA excessive excision by EXO1.^265^ Immediately following the recognition step, the removal step occurs. The mismatched bases are removed, and then the replacement DNA is synthesized by DNA polymerase δ while DNA ligase ligates the remaining nick.^259,266,267^ Certain proteins are also necessary for this synthesis step. Proliferating cell nuclear antigen (PCNA) functions not only in the mismatch recognition step but also in the processing of DNA polymerase during the final synthesis step.^268,269^ As reported by Kira CB et al., PCNA can trigger other proteins to cut the error-containing strand, leading to more rapid and effective excision and synthesis.^270^ Due to their critical roles in DNA damage repair and, in particular, mismatch repair, loss of expression of essential proteins such as MSH, EXO and PCNA is closely and significantly linked to increased predisposition to a number of diseases, including various cancers^202^ and other metabolic pathologies.^201,202,271–273^ Recent reports indicate a broader spectrum of non-canonical roles of mismatch repair. These roles include the responses to oxidative DNA lesions, helix-distorting nucleotide lesions and environmental chemical toxicants such as benzo(α)pyrene-induced cellular senescence, as well as regulation of the cell cycle.^269,274–276^ Undoubtedly due to its importance in maintaining genomic stability, mismatch repair deficiency leads to increased DNA mutations. The opportunity for secondary mutations increases by 100 or even 1000 times in mismatch repair-deficient cells.^201,277^ With the development of high-throughput sequencing technology and compound screening strategies, many novel functions and mechanisms of mismatch repair have been identified, and further research should aim to clarify the genes and proteins of the mismatch repair pathway in depth and elucidate how each gene or protein may differentially function in each step. Moreover, non-canonical roles of the mismatch repair pathway should be further investigated to provide new insights into DNA damage repair and identify potential new fields for targeted cancer therapy or improvement of chemo-and radio-therapy outcomes in the future.

**Fig. 5:**
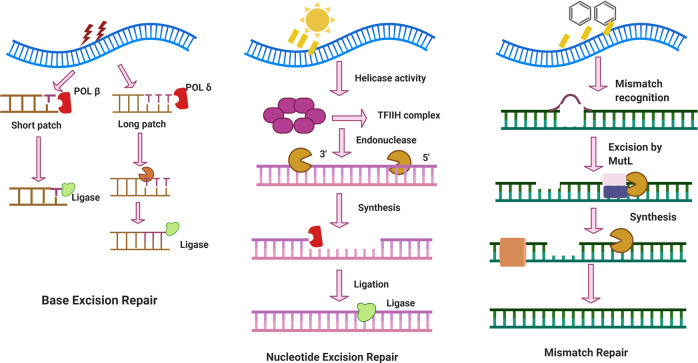
Main characteristics of base excision repair, nucleotide excision repair and mismatch pathways and the main differences in their lesion sensors, mediator proteins and effector proteins

Figure 5 illustrates the main characteristics of base excision repair, nucleotide excision repair and mismatch pathways and the main differences in their lesion sensors, mediator proteins and effector proteins.^278^ Generally, in base excision repair, APE1 and DNA glycosylases are the main DNA damage sensors. Meanwhile, the damage sensors in nucleotide excision repair are XPC and CSA; the mediator proteins are XPA, XPF and RPA; and the effect or proteins are XPG, ERCC1, and POLD1. In mismatch repair, the roles of synthesis proteins and protein complexes differ.

Single-strand break repair can be conducted through the base excision repair, nucleotide excision repair and mismatch repair pathways.^279^ In this section, we focus on the DSB repair pathway, as this damage type is most deleterious to genomic stability.

### Double-strand break repair

Two main types of DSB repair pathways have been reported, namely, homologous recombination (HR)^280^ and non-homologous end joining (NHEJ).^281^ Compared to the NHEJ pathway, HR is more conservative and error-free due to its dependence on the existence of sister chromatids.^282,283^ However, this property limits the HR pathway repair to the cell cycle S/G2 phase when sister chromatids exist, whereas the NHEJ pathway can repair DSBs throughout the cell cycle.^284–286^

#### Homologous recombination (HR)

The HR pathway is comprised of three processes: (i) double-strand break recognizing, DSBR; (ii) synthesis-dependent strand annealing, SDSA; and iii) break-induced replication, BIR. After a DSB occurs, a single-strand is cut from the DSB’s end to form two single-strand ends, of which the 3-terminal ends pair with the homologous templates to form a D-loop structure. The single-stranded DNA (ssDNA) that breaks after this step is synthesized using homologous DNA as a template. After D-loop formation, the repair modes differ among the three pathways. The resected 3′ ssDNA end(s) of the DSB sense, bind with, and insert a homologous DNA sequence to prime the synthesis of repaired DNA. Somatic cells use the sister chromatid rather than the homologous chromosome as the repair template. In the DSBR pathway, the D-loop structure becomes stable through capture of another 3′ terminus, forming a double Holliday junction (dHJ) structure. In the SDSA pathway, the 3′ end is extended and released from the template, and then matches with another 3′ end to continue the DNA repair process. In the BIR pathway, the D-loop forms a replication fork to ensure synthesis of the following chain and leading chain. Within the HR pathway, many proteins must combine with each other to function. After the formation of DSBs, under the action of specific nucleases (e.g., the Mre11-Rad50-Nbs1, or MRN, complex), the 5′ ends of DSBs are excised to form a 3-terminal single-stranded DNA (ssDNA). Subsequently, the ssDNA is encapsulated by RPA, which is replaced by Rad51 to form the nuclear fiber structure of Rad51. Mediators such as RAD52 and BRCA2 participate in this process. Subsequently, with the help of PALB2 and rad51ap1, the Rad51 nuclear fiber combines with the homologous double-stranded DNA to form a D-loop structure. After this association, the D-loop dissociates under the action of FANCM to form a product without cross exchange. The dHJ structure formed in the DSBR repair pathway can also be dissolved by a helicase topoisomerase complex (BLM-TopoIII) to form a product without cross exchange.

Rad51 plays an important role as the core molecule in the HR pathway.^287^ Rad51 in mammalian cells is similar to Rad51 in yeast cells and RecA in bacteria, with specific functions before, during, and after HR association.^288^ First, Rad51 interacts with DNA to form the Rad51 nuclear fiber structure, which effectively elongatesss DNA and is therefore conducive to ssDNA encountering its homologous DNA template.^289^ Rad51 can promote the combination of ssDNA with homologous DNA templates and, thereby, promote formation of the D-loop. After association, Rad51 breaks away from the leading strand of DNA during DNA synthesis, exposing its 3-terminal sequence, which is used as the primer for DNA synthesis.^290^

RPA is a single-stranded DNA-binding protein comprised of a trimer of RPA1, RPA2 and RPA3.^291^ RPA1 has four domains in the trimer, which play roles in DNA synthesis.^292^ The N-terminus of RPA1 has a protein-binding domain, DBD-F, along with three domains that bind to ssDNA, DBD-A, DBD-B and DBD-C. The second large component of the trimer, RPA2, has a central structure domain, DBD-D. RPA3 has only one domain, DBD-E.^293^

CtIP (CtBP-interacting protein) plays important roles in cell cycle regulation and DNA damage repair.^294,295^ It contains a dimerization domain (40–165 amino acids) at the N-terminus, which has the same amino acid sequence and binding site as the RB family. CtIP possesses a central domain, which interacts with CtBP. The C-terminal sae2/ctp1-like domain of CtIP is conserved between human and yeast. The phosphorylation of the CtIP S327 site promotes its binding to BRCA1, which then binds to itself, and then is ubiquitinated by BRCA1 and recruited to the damage site.^296^ The DNA-binding domain of CtIP is located between amino acids 515–557, which is conducive to the recruitment of CtIP to DSB sites. The two lysine sites in this domain, K513 and K515, are crucial to the interaction between CtIP and DNA. The N-terminus and C-terminus of CtIP contain structural domain segments that interact with MRN. The T847 site of CtIP can be phosphorylated by CDK, which helps CtIP to activate the nuclease activity of MRN and, thus, to promote the single-strand excision of DNA DSB ends.^297^

#### Non-homologous end joining (NHEJ)

In the classical NHEJ pathway, the heterodimer of Ku70 and Ku80 first binds to the broken DNA ends and then recruits DNA-PKcs (DNA-dependent protein kinase catalytic subunit). DNA-PKcs is a member of the phosphatidylinositol 3-kinase (PIKK)kinase family that can pull two broken DNA ends together and recruit processing-related enzymes, such as Artemis, PNKP (polynucleotide kinase/ phosphatase), APE1 (AP endonuclease 1) and Tdp1 (tyrosyl DNA phosphatase 1), and then recruit the XRCC4-XLF-LIG4 complex.^298^ Ku70 and Ku80 are subunits of the first protein complex to be recruited to the damage site, both of which have a central domain (Ku core) that binds to DNA. An acid domain, serine 6, is present in the N-terminus of Ku70 that can be phosphorylated by DNA-PKcs. SAP (SAF-A/B, Acinus and PIAS) possesses a C-terminal domain.^299^ There is a linking region between SAP and the Ku core of about 536–560 amino acids. Both SAP and this linking region can bind to DNA, so SAP may anchor the Ku dimer to chromatin. The C-terminal region of Ku80 interacts with the Ku core through a highly flexible linking region.^300^ At the end is a 12-amino-acid region that can directly interact with DNA-PKcs. The Ku dimer can recruit DNA-PKcs, XRCC4 and XLF to a damage site. When the Ku dimer binds to DNA, Ku70 is directed toward DSBs, while Ku80 is directed away from DSBs.^301^

DNA-PKcs, as a member of the PIKK family of serine/threonine protein kinases,^302^ contains a leucine-rich domain (LRR) at the N-terminus, which may play an important role in DNA binding, and a series of heat repeat sequences (huntingtin, elongation factor 3, a subunit of protein phosphate 2a and tor1, heat). The C-terminus contains a FAT (FRAP, ATM, TRRAP) domain. The PIKK regulation domain (PRD) may be located between the kinase domain and the FAT domain.^303^ Cells lacking DNA-PKcs showed high radio sensitivity. Moreover, mouse experiments showed that mice lacking DNA-PKcs might suffer from severe comprehensive immunodeficiency (SCID).^304^ The binding region between DNA-PKcs and Ku is located in the C-terminal region of DNA-PKcs. When the Ku dimer is on DNA, Ku recruits DNA-PKcs to the DNA break terminus, and two DNA-PKcs molecules interact with the DSB site to form a synaptic complex. The DNA-PKcs/Ku/DSB complex can fix the ends of DSBs, thereby protecting the DSB site from nuclease digestion.

After the end of DNA is processed by Artemis and other end-processing molecules, the subsequent repair process must connect the disconnected DNA, and LIG4 executes the DNA connection. XRCC4 has no known enzymatic activity, but can function as a scaffold protein, aiding the recruitment of other NHEJ pathway molecules. In structure, the best binding ligand of XRCC4 is LIG4.^305^ The C-terminus of LIG4 contains two BRCT domains, with a connecting region between the two domains. The highly stable complex XRCC4-LIG4 can be formed through interaction with the helical region at the C-terminus of XRCC4. XRCC4 can stabilize LIG4 and promote its activity. The XRCC4-LIG4 complex can interact with Ku, PNK, APLF, XLF and DNA.^306^ XRCC4 can be highly phosphorylated, and DNA damage can promote its self-phosphorylation. DNA-dependent protein kinase (DNA-PK)is necessary for the phosphorylation of XRCC4 induced by DNA damage, and promotes the binding of XRCC4-LIG4 to DSBs.^307^ The SUMO modification of XRCC4 is essential to its nuclear localization and DSB repair function.

53BP1 (p53-binding protein 1) is a very important molecule in the DSB repair pathway, functioning as an intermediary molecule or effector.^308^ It can promote the terminal junction of DNA after DSB occurrence. To be recruited to DNA, 53BP1 must directly recognize the specific histone structure produced by the DSB. Moreover, 53BP1 can promote the NHEJ pathway and inhibit the HR repair pathway. The N-terminus of 53BP1 contains 28 serine/threonine glutamine sites (s/t-q), which are the target sites of ATM. When the N-terminus of 53BP1 is phosphorylated by ATM, the interaction of 53BP1 with Rif1 (Rap1-interacting factor 1) and PTIP(Pax activation domain-interacting protein) is promoted.^309,310^ The C-terminus of 53BP1 contains a BRCT domain, which interacts with p53 and EXPAND1. The minimal focal region of 53BP1 contains an OD (oligomerization domain), a glycine- and arginine-rich (GAR) motif, and a ubiquitination-dependent recruitment (UDR) domain. It can be dimethylated atlysine 20 within its GAR motif, and the UDR domain can interact with ubiquitinated H2AK15.^311^

#### Alternative end joining

While the c-NHEJ and HR pathways are primarily responsible for repairing DSBs of DNA, alternative end joining (alt-EJ) was considered responsible for residual DSBs that c-NHEJ and HR are unable to repair.^312,313^ However, it is unsure whether alt-EJ represents a standing pathway or only the end-joining components of the pathway usually serving in dsDNA processing of other functions, such as in replication, recombination or repair. Alt-EJ is also called microhomology-mediated end joining(MMEJ).^314^ Alt-EJ refers to repair of DSB damage independently of classical NHEJ factors such as Ku70, DNA-PKcs and lIG4.^315^ Although this process appears similar to c-NHEJ, alt-EJ is Ku-independent, depending instead on regions of microhomology on each side of the breakage site.^315^ Specific proteins including PARP-1[Poly(ADP-ribose) polymerase] are critical for facilitating the alt-EJ pathway.^316^ As reported by Huang YJ et al., PARP-1 is vital to DSB repair in breast cancer cells, and the alt-EJ pathway is triggered by radiomimetic agents.^317^ Other studies have shown that PARP-1 and DNA ligases are required for chromosomal translocation followed by alt-EJ activation due to ionizing radiation.^318^ In addition, alt-EJ is mainly mediated by the CtIP/MRN complex.^319^ Furthermore, Polθ can indicate the microhomologous DNA ends to support the joining of DNA ends.^320^ Moreover, the maximum activity of the alt-EJ pathway was observed in the G2 phase of the cell cycle.^321^ In mammalian cells, PARP-1 binds completely to the DNA ends with the Ku heterodimer, and resection is then triggered by the MRN complex, followed by DNA ligase III mediating DNA end ligation.^322–324^ DNA polymerase theta (pol teta) is an evolutionarily conserved protein encoded by the *POLQ* gene in mammalian genomes^325^ with the ability to mediate joining of single-stranded 3′ ends. Without pol theta, end joining is damaged and residual repair would create large deletions flanking the break site.^326^

**Fig. 6:**
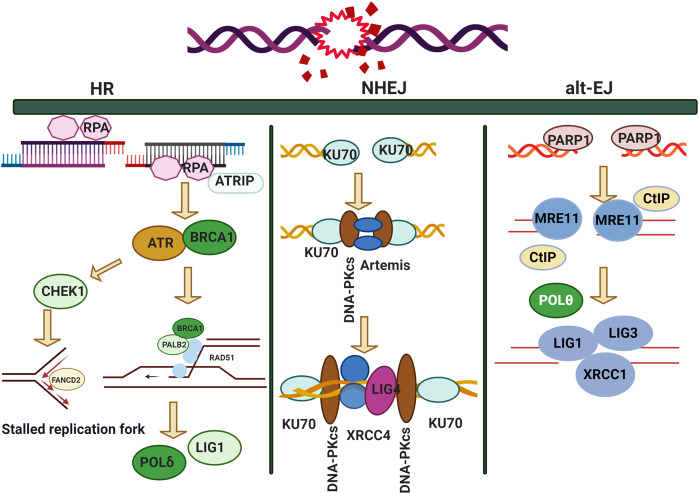
DNA DSB repair pathways with the roles of the relevant proteins

Figure 6 illustrates DNA DSB repair pathways with the roles of the relevant proteins. As shown in Fig. 6, DSB repair depends on whether end resection occurs. If the end resection process is blocked, the only repair pathway available is NHEJ, whereas if end resection occurs, three repair pathways, namely HR, NHEJ and alt-EJ, can be initiated to repair lesions in a competitive model. The competitive model includes two layers, as NHEJ competes with the resection-dependent pathways, while HR and alt-EJ compete for lesion repair. The consequences of these three pathways differ. The outcome of NHEJ is accurate deletions of 1–4 nt, while the outcome of HR is loss of heterozygosity, and that of alt-EJ is mutagenesis rearrangement (insertions/deletions). In the review by Anabelle D, this pathway was described as relying on a subset of HR enzymes, and alt-EJ is a highly mutagenic pathway in vivo, driving telomere fusion events and tumor-related chromosomal translocations in various mouse models.^327^ These findings raise vital questions about the rules for selecting a repair pathway and the consequences of each option for DSB repair.

According to the review by Raphael et al.,^97^ certain factors may influence DSB damage repair pathway selection. First, the role of end resection affects the DDR choice.^328^ Nicole et al. indicated that resection is commonly used by cells for selection of DSB repair pathways,^329^ especially in the G1 phase of the cell cycle or with complex damage. End resection includes two phases, of which the first is “end clipping”, where in a small number of base repairs occur, making the DNA ends available for alt-EJ.^314^ In the second phase, known as “extensive resection”, proteins including CtIP and EXO1 produce long stretches of ssDNA, which are then subject to HR.^330,331^ Thus, the factors that affect the end resection process also influence DNA DSB repair pathway selection. For example, CDKs phosphorylate the long-range resection nuclease EXO1 in the S to G2 phases of the cell cycle, regulating the DNA end resection and repair pathway choice.^332^ However, in non-cycling cells, DSB end resection is significantly reduced, facilitating the c-NHEJ repair pathway.^97^ Post-translational modification was also found to regulate end resection. For example, acetylation plays a dual role, inhibiting end resection in budding yeast while promoting end resection via sumoylation.^333,334^ Sonia et al. demonstrated that protein deneddylation is another major controller of DSB repair pathway choice. RNF111/UBE2M-mediated neddylation can inhibit end resection mediated by CtIP through regulation of ssDNA length.^335^ Additional factors, such as maintenance of the balance between BRCA1- and 53BP1-mediated end resection, also modulate pathway selection. In BRCA1-knockout cells, 53BP1 can end resection by blocking CtIP from DNA ends, leading to c-NHEJ pathway selection.^336^ Furthermore, hyperactivity of 53BP1 drives genomic instability in BRCA1-/- mice through inhibition of HR pathway selection.^337^ In addition to these factors associated with end resection, the repair pathway-related protein complex can affect complex formation or dissociation, and may thus also influence end resection, as the linkage among the protein complexes is likely to be an elaborate interaction network. Alteration of either protein in this network may affect end resection, leading to resection-dependent repair pathway selection.^338^ A recent study showed that ATM mediated the interaction of the UBQLN4-MRE11 complex to repress HR further, indicating that ATM not only initiates the HR pathway but also suppresses excessive end resection through various protein interactions.^339^ Importantly, competitive relationships among various DSB repair pathways have also been reported. For example, an enzyme in the alt-EJ pathway, Polυ, can inhibit HR pathway activity through binding with RAD51, indicating that Polυis vital for alt-EJ to compete with the HR pathway.^340^ In contrast, HR factors such as FA (Fanconi anemia) proteins also promote alt-EJ activity,^341^ suggesting that these factors, which interact with ATM and RAD51, can influence repair pathway selection in a background-dependent manner.^97,342^ In general, these studies confirm that multiple connections exist between alt-EJ and HR. These connections may be competitive and one protein may function in multiple pathways under multiple avenues of regulation, increasing the complexity of DSB repair pathway selection.

**Fig. 7:**
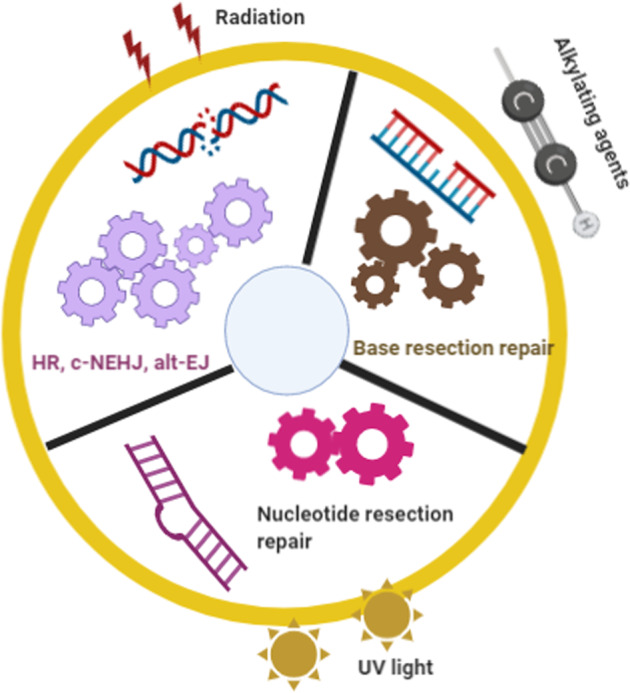
Hypothesis of “environmental gear selection” to describe DNA damage repair pathway evolution. Environmental hazards such as radiation, ROS, alkylating agents, cross-linking agents, topoisomerase inhibitors, and UV light can affect DSB repair pathway selection

Over the past decade, many hypotheses as to how DSB repair pathways are selected have been proposed. From the perspective of George et al., alt-EJ may operate as a backup to the c-NHEJ and homologous recombination repair pathways in the G2 phase. However, as the fidelity of alt-EJ is lower than that of c-NHEJ, chromosome translocations become more likely.^343^ Previous reports have noted that the cell cycle and chromatin context also affect the selection of the double-strand repair pathway, and some studies have shown that euchromatin and heterochromatin both affect this choice.^344^ Shuren et al. hypothesized that the DNA end structure is another major determinant of the DSB repair pathway.^345^ However, these hypotheses were published considering single factors rather than from a global perspective of the DSB repair process. In this review, we raise a novel hypothesis based on a global view of DSB causes, repair selection and consequences. We designated this hypothesis “environmental gear selection”. As shown in Fig. 7, environmental hazards such as radiation, ROS, alkylating agents, cross-linking agents, topoisomerase inhibitors, and UV light can affect DSB repair pathway selection. For example, alkylating agents may lead to the NER and BER [Please define these abbreviations] repair pathways, whereas radiation damage may be repaired through the BER, HR, c-NHEJ or alt-EJ pathways. UV light leads to NER selection over c-NHEJ and alt-EJ. After insults from different DNA damage sources, different sensors, proteins and protein complexes are activated to initiate various repair pathways. Some proteins function in specific pathways, such as ATM and DNA-PKcs in the HR and c-NHEJ pathways, respectively. The activities of such specific proteins contribute to repair ability and subsequent repair outcomes. This process is similar to the principle of gears’ function in a hydrodynamic force model as illustrated in Fig. 7. Comparing a watch to genomic stability, the rollinggears in the watch represent the working proteins or protein complexes in a repair pathway to genetic damage. When the watch is disturbed by radiation, gear A may be chosen to trigger the BER repair pathway, whereas when the watch is lesioned by antimetabolites, causing more serious damage, the HR, c-NHEJ and alt-EJ pathway choice may be activated by gear B. At this stage, these three pathways compete depending on the cell cycle phase and end resection status. When exposed to UV light, gear C is activated to select the NER pathway. This “environmental gear selection” hypothesis may provide new insights into the environment-dependent selection among DNA damage repair pathways. In certain significance, this selection hypothesis is generated from the natural selection and can be considered as a result of evolution. Notably, this hypothesis will help clarify the roles of DNA damage, response, and repair, along with providing targets for cancer therapy.

## Targets for cancer therapy

Cancer, one of the main causes of death around the world, is a threat to human health that requires urgent attention. Currently, chemotherapy, radiotherapy and immunotherapy are the main treatment measures for cancer. Some of these therapy strategies have been found to inhibit cancer through disruption of the DDR process, interfering with DNA replication and inducing DNA lesions in cancer cells, and signaling cell death. Thus, it has been suggested that improved cancer treatment outcomes may be obtained through targeting the DDR and DNA replication along with promotion of mitotic catastrophe in cancer cells.^346^ To achieve stronger oncogene triggering ability, cancer cells generally exhibit genomic instability, cancer suppressor gene inactivation and tolerance of attenuated DNA damage repair.^347^ DDR enables cells to respond to a variety of exogenous and endogenous insults that threaten the cell’s genomic stability.^348^ A number of essential components of this response are enzymes and proteins, which are encoded by various genes.^349,350^ The specific activation or inactivation of these factors in various cancers and the development of corresponding inhibitors or activators represent a recent hot spot of cancer therapy research.^351^ However, some studies have found that inducing DNA damage in cancer cells during cancer therapy may have unfavorable side effects.^347^ For example, the cancer suppressor proteins related to DDR, DNA-PK and CHK1, exhibit oncogenic functions in the later period of cancer development.^352^ Moreover, much serious obstacle to cancer prevention and control is cancer cell resistance to therapy.^353–355^ Numerous studies have discussed this problem of resistance, some in the cancer microenvironment and others using cancer stem cells^356^ or cancer heterogeneity.^357^ In this review, we focus on the targeting of DDR and repair-related proteins, kinases and pathways to explore possible cancer therapies.

### Targeting the DNA damage response for cancer immunotherapy

#### Pivotal milestones in cancer immunotherapy

Immune evasion, a hallmark of cancer cells, causes difficulty and frequent failure of cancer therapies aimed at activating the immune system against malignancy. A recent review described the immune escape mechanisms in lung cancer. The authors noted that specific mechanisms, including impaired antigen presentation and activation of immune checkpoints, lead to immunotherapyresistance.^358^ Despite this difficulty, the application of immunotherapy has changed the treatment of clinical cancer patients in recent decades.^359^ In particular, the advancement of immune checkpoint blockade therapy provides hope for improving therapy outcomes and life quality in future cancer sufferers.^360,361^

From a historical perspective, the earliest application of immunotherapy for cancer treatment occurred more than a century ago. The first therapeutics applied, Coley’s toxins, were used by Dr. William Coley et al. to stimulate the immune systems of cancer patients using a complex of heat-killed bacteria.^362^ Based on this treatment, Dr. William Coley came be called the “Father of Immunotherapy”.^363^ Dr. William Coley reported that he was able to use these toxins successfully to induce cancer regression.^364^ At the time, his reports were highly controversial and received strong criticism from other researchers, who criticized the efficacy and safety of toxins used for cancer treatment.^365^ In 1909, Paul Ehrlich proposed the hypothesis that cancer cells can protect themselves through immune recognition.^366^ Almost 50 years later, Mithison et al. observed passive transfer of anticancer immunity in transplanted mice. A milestone in cancer immunotherapy development was the discovery of CTLA-4 in 1985.^367^ Walunas et al. found that CTLA-4 is associated with glycoproteins found in T cells. Its expression increases with the activation of T cells.^368^ In 1992, the United States FDA (Food and Drug Administration) approved high-dose IL-2 therapy for clinical use, which enables expansion of cultured lymphocytes.^369^ Soon thereafter, PD-1 (programmed death ligand 1) was discovered, also in 1992.^370^ Then, in 1994, Allison et al. reported cancer rejection following anti-CTLA-4 blockade.^371,372^ Since then, numerous advances have been achieved. In 2002, a very important hypothesis was proposed by Schreiber et al., known as the immunoediting hypothesis, which states that cancer cells play dual roles of promoting host protection against cancer and also escaping immune destruction.^373^ Then, CAR-T cells were found to elicit clinical responses in patients with B-cell lymphoma.^374^ The first vaccine to treat cancer was developed from dendritic cells by Dendreon Pharmaceuticals, called sipuleucel-T, and was approved by the FDA in 2010.^375^ In 2011, the FDA approved the CTLA-4 blockade agent, ipilimumab, a human IgG1l anto-CTLA-4 monoclonal antibody,^376^ which is the first FDA-approved immune checkpoint inhibitor.^377^ Following this approval, the PD-1/PD-L1 blockade agent nivolumab was approved by the FDA in 2014.^378^ Immunotherapy with nivolumab and ipilimumab is now the first-line therapy for patients with unresectable malignant pleural mesothelioma.^378,379^ In 2015, the first, and to date only, oncolytic virus, talimogene laherparepvec, was approved by the FDA for cancer immunotherapy.^380,381^ This virus has the ability of inducing oncolysis directly and activating adaptive anti-cancer immunity.^382,383^ Interestingly, in 2018, a study reported that the gut microbiome influences the efficacy of checkpoint blockade.^384^ In a study by Gopalakrishnan V et al., the gut microbiome modulated the tumor response to checkpoint blockade immunotherapy in mouse models.^385^ Meanwhile, a human clinical test showed that primary resistance to immune checkpoint inhibitor therapy could be attributed to dysregulation of gut microbiota composition and abundance.^386^ These are the major milestones in the history of cancer immunotherapy. Now, as we begin a new century, the main priority is exploitation of the mechanisms underlying immunotherapy, while further research should also be conducted to identify new checkpoint inhibitors.

#### DNA damage response deficiency is associated with activation of anticancer immunity

Compelling evidence has shown that DNA damage signals and endogenous DDR can activate the innate immune response.^387^ Some reports have noted an association between DDR deficiency and the activation of anticancer immunity. The cGAS-STING pathway is a canonical defense mechanism against viral infections, in which the cGAS (cyclic GMP-AMP synthase) can detect and sense the exogenous viral DNA entered into the cytosol, stimulates the adapter protein STING (Stimulator of interferon genes) to trigger interferon (IFN) signaling. Two research groups SM Harding, et al. and KJ Mackenzie, et al. simultaneously reported that cGAS can also sense the cytosolic micronuclei DNA originated from the nuclear chromosomal fragments following DNA double-strand breaks induced by ionizing radiation. Upon binding the cytosolic DNA, cGAS produces the second messenger cGAMP, which in turn activates STING- inflammatory signaling, and leading to regression of abscopal tumors.^388,389^ Moreover, a non-canonical cGAS-independent STING activation pathway has also been revealed to be triggered by the DNA damage signaling in nucleus.^390^ It was found that upon genomic DNA damage, the STING was activated by the DNA repair proteins ATM and PARP1,together with the DNA sensor IFI16 (interferon-inducible protein16).^391^ On the basis of summarizing the literatures regarding the activation of cGAS-STING pathway by radiation-induced DNA damage signaling, Storozynsky and Hitt concluded that the cGAS-STING signaling pathway is a bridge between the DNA damaging abilities of exogenous toxicants and the activation of CD8+ cytotoxic T cells, showing that this pathway can induce anticancer immune responses.^392^ Moreover, the DNA damage-induced alt-EJ pathway can also induce an innate immune response.^393^ As reported by Dunphyet al., ATM and PARP-1, which are DDR factors, can combine with the DNA-binding protein IFI16(TNF receptor associated factor 6) to activate STING through the alt-EJ pathway, leading to the assembly of an alternative STING signaling complex that contributes to the recruitment of the tumor suppressorp53 and the E3 ubiquitin ligase TRAF6 to STING.^391^ TRAF6catalyzes the K63-linkedubiquitination of STING, which promotes the activation of nuclear factor kappa B (NF-κB).These data indicate that DNA damage-mediated signaling pathway activation can promote anticancer immune responses.

Because of their intrinsic characteristic of genomic instability, cancer cells may trigger innate immunity through activation of a series of inflammatory pathways aimed at facilitating the targets of cytotoxic lymphocytes,^394^ such as the report showing that IFN-gamma is critical for cytotoxic T cell-dependent genome immunoediting of cancer cells.^395^ Insufficient genome integrity due to DNA repair and replication defects may also activate the innate immune response. Recently, concern has been raised about how cellular DNA is driven out of the cytoplasm, which causes a cascade effect. Some studies have indicated that EXO1, BLM and SAMHD1 can promote double-strand DNA damage and stalling of the replication fork, thereby releasing genomic fragments into cytoplasm.^396,397^ These fragments produced in response to exogenous insult such as radiation exposure can also activate the cGAS-STING signaling pathway, indicating that cGAS is linked to a subset of the induced micronuclei and downstream cascade events of IRF3 phosphorylation and increased IFN-α expression.^388^ A famous discovery is that the abrogation of BRCA1 or BRCA2, which are cancer suppressors, causes DSB accumulation and induces IFN signaling and anti-cancer immunity.^398^ These findings suggest that an effective therapeutic strategy can be obtained through combined usage of genotoxic agents and immune checkpoint blockade.^389^ Furthermore, these studies provide further evidence that cancer-causing genomic instability is typically associated with the induction of innate immunity.

#### Cancer cells have the capacity for immune escape

**Fig. 8:**
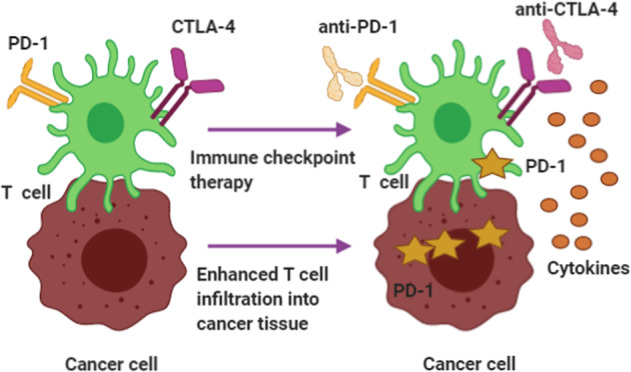
Immune checkpoint blockade promotes the T-cell response

Multiple studies have described the ability of cancer cells to escape from immune system detection and attack, which depends in part on expression of cell surface proteins that perform immunosuppressive functions.^358,399^ Vital proteins such as PD-L1^400^and cytotoxic T-lymphocyte-associated antigen 4(CTLA-4) are expressed on the surface of T cells. Their receptors may interact with B7 to ensure the inactivation of T cells.^401^ As a result, immune checkpoint blockade would promote adaptive immunity and inhibit cancer proliferation and growth.^402^ From the perspective of DDR, a hallmark of cancer, the immune system response can be initiated through induction of cell surface ligands to trigger disruption of cancer surveillance by a series of immune receptors, such as NKD2D.^403^ This finding indicates that DDR can act as a bridge between alteration of cancer cell surface immune-related receptor expression and the cancer cell immune response.^404^ Indeed, prior to elucidating how cancer cells develop the capacity for immune escape, it is essential to determine how immune sensors detect nuclear DNA damage and how nuclear DDR sensors sense immune signaling molecules in the cytoplasm.^405^ Nakad et al. noted that a series of sensors, including protein complex of replication protein A (RPA) and MRN, can detect DNA damage and activate the TKR9 signaling pathway, inducing further NF-κB expression in the nucleus, while in the cytosol, the Ku70 complex can sense DNA to activate the IFN-regulatory factor (IRF) response and the innate immune adapter CARD9 can trigger NF-κB through activation of the STING signaling pathway.^405^ DDR is not only able to induce inflammatory signaling factors as described above but also induces ligands to bind to the immune receptors NKG2D and DNAX Accessory Molecule-1 (DNAM-1).^406^ However, the upregulation of these ligands requires activation of DNA damage sensors such as ATM and ATR protein kinases, as well as checkpoint pathway-related proteins such as Chk1.^407,408^ In cancer cells, these mechanisms have been demonstrated to have significant associations with immune escape. In a study by Masahisa J et al., a DNA damage signal mediated by ATM facilitates cancer immune escape and increases cancer cell resistance to phagocytosis^409^ in an integrin αvβ3-dependent manner. A recent study found that DNA-PKcs, a well-known protein in the non-HEJR pathway that plays a pivotal role in genome stability, also has functions in immune escape.^410^ In addition, p53, a vital cancer suppressor, activates the innate immune response against cancer cells and can directly control immunosurveillance to achieve successful cancer therapy.^411^ Thus, DDR-related sensors and protein complexes as well as the immune escape mechanism are very important to cancer immune therapy. In Fig. 8,we illustrate how immune checkpoint blockade promotes the T cell response. As shown in Fig. 8, after T cells are activated, the expression of immune checkpoints including PD-1 and CTLA-4 increases, and then T cells are able to enter the cancer tissues, causing the cancer tissues to release large amounts of cytokines such as IFN-γ and ultimately resulting in increased expression of immune checkpoints. Anti-immune checkpoints can block immune checkpoint expression and in turn promote the T cell response.

#### Clinical application of immune checkpoint inhibitors based on the DNA damage response

The PD-1/PD-L1 axis is well recognized as a promising therapeutic target.^412^ The agents that regulate PD-1/PD-L1 axis expression can thus be considered possible clinical cancer immune therapies. To date, several immunomodulatory agents targeting the PD-1/PD-L1 axis have been approved by the US FDA. These agents include avelumab, durvalumab, pembrolizumab and ipilimumab. Table 1 lists the clinical trial outcomes of typical agents targeting the PD-1/PD-L1 axis and CLA-4. The anticancer activity, safety and tolerance of these targeting agents have been supported in a series of clinical trials (Table 1), but many challenges and limitations to their application remain. (i) Although the selection of predictive biomarkers from genomic targets for clinical cancer therapy is essential for successful treatment, for immunotherapy, the checkpoint blockade of immune cell responses to the treatment agents is more important.^413^ Unlike the genes that are mutated in cancer cells, such as p53, the immune system is significantly affected by stressors including complex environmental toxicants and endogenous materials. Based on this consideration, future research may shift its focus to immune response effectiveness rather than the identification of additional biomarkers for immune checkpoint-related mechanistic studies. The effectiveness of an evolving immune response indicates the major clinical benefits provided by immune therapy to cancer patients. These benefits should be further evaluated in the clinical setting. (ii) Among cancer cells, immune cells do not exist alone; instead, many cell types including fibroblasts and endothelial cells make the microenvironment around cancer cells highly complex, providing opportunities to escape immune detection and monitoring. Thus, the targeting of immunotherapy agents should focus on uncovering the underlying mechanism of immune escape and the relationship between the cancer cell microenvironment and immune cell responses in terms of therapy efficacy. (iii) The mechanism through which immune T cells provide the therapeutic response remains unclear. Because this response process is generally complex, involving numerous signaling pathways, its regulation may be either stimulatory or inhibitory. This complexity causes great difficulty in the transfer and application of clinical agents. Thus, an in-depth understanding of the regulation pathway of immune T cells in response to environmental insults and therapeutic agents is urgently needed. (iv) It has been suggested that the combination of multiple therapeutic agents may be more effective than a single application. However, which agents are best used in combination and their safety and efficacy in clinical settings remain unknown. More importantly, the molecular mechanisms underlying the effects of combination therapy on the immune T cell response require further investigation. Nevertheless, immune checkpoint therapies and combinations of multiple agents have provided robust evidence of their benefits to clinical patients and have the potential to elicit durable control, and even curing, of cancer.^413–415^ (v) Based on the current understanding of immune therapy causing checkpoint blockade, factors that can activate T cells and increase immune checkpoints, such as PD-1/PD-L1 and CLTA-4,are promising agents for future application. For example, new evidence has shown that human microbiota play roles in immunotherapy. According to Vanessa et al., *Bifidobacterium* spp. probiotics can improve the effects of PD-1 and CTLA-4.^416^ The authors suggested that in the era of immunotherapy, immunotherapies that are more selective and effective while being less toxic should be investigated. Thus, action should be taken immediately to reveal the mechanisms of the T cell activation and response processes under various environmental stresses and endogenous insults, which would identify potential effective agents for future clinical use.

**Table 1:** Immune checkpoint inhibitors and their association with DNA damage response

Target	Drug name	Cancer type	Trial	Outcomes	Reference
PD-L1	LY3300054	Advanced solid cancers	phaseI1/Ib trial	As of a new PD-L1 inhibitor, LY3300054 showed well tolerated without unexpected safety concerns	^549^
PD-L1	Atezolizumab	Non-small-cell lung cancer	Randomized control, phase III	Atezolizumab treatment resulted in significantly longer overall survival than platinum-based chemotherapy	^550^
PD-L1	Avelumab	Urothelial cancer	Randomized control, phase III	Avelumab also significantly prolonged overall survival in the PD-L1-positive population	^551^
PD-L1	Durvalumab	Urothelial cancer	Randomized control, phase III	Compared to the chemotherapy, Durvalumab treatment had a higher median overall survival time in PD-L1 high expression population	^552^
PD-L1	Nivolumab	Hodgkin lymphoma	Randomized control, phase II	Compared to the chemotherapy, Nivolumab treatment led to rapid near-complete reduction of cancer in early-stage unfavorable Hodgkin lymphoma	^553^
PD-L1	Cemiplimab	Cervical cancer	Phase I cohorts	Cemiplimab has anti-cancer activity for cervical squamous cell cancer	^554^
PD-1	Pembrolizumab	Colorectal cancer	Randomized control, phase III	Pembrolizumab led to significantly longer progression-free survival than chemotherapy	^555^
PD-1	Dostarlimab	Endometrial cancer	Non-randomized phase1	Dostarlimab has clinically meaningful and durable anticancer activity for endometrial cancer patients	^556^
PD-1	Nivolumab	Non-small-cell lung cancer	Retrospective cohort study	Advanced non-small-cell lung cancer patients treated with Nivolumab had a long term survival rates	^557^
CTLA-4	Tremelimumab	Malignant mesothelima	Randomized control, phase II	Tremelimumabhas clinically meaningful and durable anticancer activity with acceptable safety and tolerability	^558^
CTLA-4	Ipilimumab	Pancreatic cancer	Phase Ib study	Ipilimumab is a safe and tolerable agent for pancreatic ductal adenocarcinoma therapy	^559^

### Targeting DNA damage repair for cancer chemotherapy

Chemotherapy is currently the most common clinical option for cancer treatment.^417^ As discussed above, BER, NHEJ, alt-NHEJ and HR are the four major repair pathways of DNA damage repair. The key proteins in BER are PARP-1 (poly(ADP-ribose) polymerase), APE1(abasic endonuclease), XRCC1(X-ray cross-complementing protein 1) and DNA ligase III; those in the NHEJ pathway are KU70/Ku80, DNA-PK, Artemis, XRCC4, DNA ligase IV and XLF; the alt-NHEJ pathway includes PARP-1, XRCC1 and DNA ligase III; and HR involves RPA, BRCA1, PALB2, BRCA2 and RAD51.^418,419^ According to numerous studies, DNA repair pathway defects facilitate genomic instability, leading to increased cancer cell proliferation and survival time.^420^ Nonetheless, cancer cells depend on the residual DNA repair capacity to protect them against damage. In cancer chemotherapy, therapeutic agents applied in the clinical setting rely on mechanisms of inducing DNA lesions, resulting in cell death. Therefore, in this section, we focus on targeting these key proteins as potential cancer chemotherapy agents.

#### Targeting PARP-1

Numerous studies have shown that PARP-1 performs critical functions in many biological processes, including regulation of transcription, apoptosis, and DDR.^421^ In particular, once a single-strand break or DSB occurs, the expression of PARP-1 is activated, enhancing poly(ADP-ribose) (PAR) activity, which adds branched PAR chains to enhance recruitment of repair proteins to the damage location.^422,423^ This function was first identified in 2007 by Fisher et al.^424^ in an investigation of the DNA single-strand breakage mechanism. Since then, further evidence has shown that PARP-1 also has a repair function in the alt-EJ pathway.^425^ Inhibitors targeting PARP-1 have been used as anticancer therapies, with the characteristic mechanism of catalyzing the PARP active site through competitively binding with NAD^+^.^426^ PARP-1 inhibitors have demonstrated effectiveness for the treatment of cancers characterized by BER and alt-EJ defects. The first report of specific PARP activity inhibition was in 1971, based on the treatment of HeLa cells with thymidine and nicotinamide.^427^ Subsequent studies reported the identification of numerous compounds that inhibit PARP activity. In general, the efficacy of PARP inhibitors for treatment of cancers with BER and alt-EJ repair defects is due to such lethal synergistic interactions.^428,429^ In 2005, Helen EB et al. first indicated that knockout cells of BRCA2 showed defects in HE [Please define this abbreviation] pathway repair, which made the cells sensitive to PARP inhibitors and showed that PARP-1 activity is vital in HE-deficient BRCA2 mutant cells.^430^ Thus, in BRCA2 mutant cells, the replication fork lesion cannot be repaired and the cancer cells die. Over the past decade, the US FDA and European Medicines Agency (EMA) have approved several PARP inhibitors. These inhibitors include olaparib, which was approved in 2014 for advanced ovarian cancer therapy^431^ and for primary peritoneal cancer therapy in 2017,^432^ in 2018 for HER-2 negative breast cancer treatment,^433^ and in 2020 for metastatic castration-resistant prostate cancer;^434^ rucaparib, which was approved in 2016 for ovarian cancer therapy,^435^ in 2018 for treatment of recurring ovarian and primary peritoneal cancers, and in 2020 for treatment of metastatic castration-resistant prostate cancer; niraparib, which was approved in 2017 and 2020 for primary peritoneal cancer and advanced ovarian cancer, respectively;^436,437^ and talazoparib, which was approved in 2018 for treatment of advanced or metastatic HER2-negative breast cancer.^438^ In addition, veliparib (ABT-888) is currently in the clinical trial process,^439^ while another new inhibitor, fluzoparib, which was initially identified in 2017, is also in clinical trials for treatment of ovarian, breast and lung cancers.^440^

#### Targeting APE1

APE1, is a vital BER pathway protein that is known to be a necessary intermediate in the processing of potentially cytotoxic abasic DNA damage sites.^441^ Previous research has indicated that the biological functions of APE1 are complex. APE1 expression is ubiquitous in various normal and cancer tissues, but shows differences among tissues. Moreover, APE1 in the nucleus has been suggested to carry out a DNA repair function, whereas its presence in the cytoplasm is assumed to support regulation of mitochondrial DNA repair or regulation of transcription factors.^442^ CJ Herring et al. showed that APE1 expression was associated with intrinsic radiotherapy sensitivity in cervical cancer tissues.^443^ In lung cancer tissues, APE1 shows high expression levels, particularly in cisplatin-resistant cancers.^444^ Other studies have shown that the expression of APE1 is significantly associated with DNA repair capacity in cancer tissues such as seminomas and malignant teratomas.^445–447^ These data not only indicate that increased APE1 levels are associated with DNA damage but also that they show enhanced resistance to chemotherapy and radiotherapy. To support its use as an anticancer drug target, efforts have been made to develop drugs that inhibit APE1 activity or show strongly enhanced sensitivity to DNA base lesions. Over the past decade, a number of APE1 inhibitors have been developed through cell, animal and clinical trials. These inhibitors can be classified into the following types: APE nuclease activity inhibitors, including methoxyamine, CRT0044876, lucanthone and hycanthone; APE1 redox activity inhibitors, including resveratrol and E3330; and inhibitors of the APE1/nucleophosmin interaction, including gossypol.^448^ Michael SG et al. indicated that cancer patients treated with methoxyamine tolerated a maximum oral dose of 60mg/m^2^/d.^449^ CRT0044876, which plays a role in inhibition of apurinic/apyrimidinic (AP) endonuclease, as well as 3′-phosphodiesterase and 3′-phosphatase activities, can potentiate the cytotoxicity of DNA base-targeting compounds^450^ and is considered a potent and selective APE1 inhibitor.^451^ Lucanthone acts as an ionizing radiation enhancer as well as an APE1 endonuclease inhibitor,^452^ andis considered a novel inhibitor of autophagy that can induce cathepsin D-mediated apoptosis.^453^ Hycanthone is a derivative of lucanthone, which also shows promise as a cancer therapy.^454^ Lucanthone is a well-known topoisomerase II inhibitor.^455^ HeLa cells treated with lucanthone can exhibit AP site accumulation.^456^ Resveratrol is a natural phenol that enhances the cytotoxicity of chemotherapy in human melanoma cells.^457^ E3330 caused significant inhibition of cancer cell proliferation through upregulation of endogenous ROS levels.^458,459^

#### Targeting XRCC1

Almost 30 years ago, the gene encoding XRCC1 was cloned out and its known biological functions expanded greatly.^460^ Typically, XRCC1 is known as a molecular scaffold protein that can provide a platform for recruiting multiple enzymatic components, such as DNA kinase and DNA phosphatase, to improve the repair of DNA single-strand breaks.^461,462^ Among the functions of XRCC1, its interaction with its protein partner, PARP1, is particularly interesting. Such interactions are important for XRCC1 functions, especially for cancer cell resistance to chemotherapy.^460,463^ Accumulating evidence shows that XRCC1 mutations are strongly associated with various diseases, including neurological diseases^464^ and cancers.^465,466^ For example, the XRCC rs25487 AA genotype is linked to increased risk of severe radiation-induced lymphopenia during cancer treatment.^467^ Another study showed that XRCC-1 arg194trp polymorphism led to increased acute radiation-induced toxicity in patients with advanced laryngeal squamous cell cancer undergoing cisplatin-related chemotherapy.^468^ In a prospective cohort study, patients with head and neck squamous cell cancers with polymorphism variants had significantly increased acute radiation-related morbidities.^469^ A phase II study evaluated the effects of XRCC1 polymorphisms on the chemotherapy agents satraplatin and prednisone, monitoring therapeutic outcomes in patients with metastatic castration-resistant prostate cancer, and showed that, compared to XRCC1 polymorphisms, patients with the wild-type allele had longer progression-free survival.^470^ Currently, most XRCC1-related cancer therapy studies are focused on the effects of XRCC1 polymorphisms on chemotherapy and radiotherapy outcomes^471^ or the use of XRCC1 as a biomarker for cancer clinical outcome prediction.^472,473^ In addition, studies on the regulation of XRCC1 protein expression and its effect on chemotherapy outcomes are underway. Decreased XRCC1 expression due to gefitinib, a selective epidermal growth factor receptor-tyrosine kinase inhibitor and Hsp90 inhibitor led to synergistic cytotoxic effects on non-small cell lung cancer cells.^474^ We screened the clinical trials related to XRCC1 using the keyword “XRCC1” (https://clinicaltrials.gov/). A total of 15 trials were listed, of which most are associated with the effects of XRCC1 polymorphisms on chemotherapy outcomes.

#### Targeting DNA-PKcs

DNA-PKcs, a vital component of the NHEJ machinery, belongs to the phosphatidylinositol-3 kinase-like kinase family (PIKK). One of the important roles of DNA-PKcs is inducing ATM (ataxia-telangiectasia mutated) and ATR (Rad3-related protein) in response to DNA lesions. During NHEJ initiation, after the DNA DSB is recognized by Ku70/80, DNA-PKcs binds to Ku70/Ku80 to form a complex, leading to a conformational change in the structure of DNA-PKcs that allows the N-terminus region of DNA-PKcs to modulate the activation of enzymatic activity.^475^ However, the mechanisms underlying the interaction of DNA-PKcs with Ku70/Ku80 remain unclear. Furthermore, the reason why multiple sites of DNA-PKcs is frequently phosphorylated by a number of kinases including PIKK members ATM and ATR, but few phosphorylation events other than S2056 and T2609-T2647 cluster were clearly confirmed in the NHEJ process, has not been fully elucidated.^476,477^ In the final stage of the NHEJ pathway, break gaps are filled by a series of DNA synthases and DNA ligase IV. DNA ligase can be stabilized and triggered by XRCC1, forming XRCC4-DNA ligase IV complexes that further ligate non-cohesive ends of DNA in the final step of NHEJ. In addition to the DNA repair-related function of DNA-PKcs, it has other functions including roles in mitosis regulation, protection of telomeres, and the inflammatory and immune responses.^478,479^ These data indicate that the biological functions of DNA-PKcs are critical. Our lab team has extensively investigated the functions of DNA-PKcs over the past three decades. We have identified expression level changes in hepato- and cholangio-carcinomas,^480^ and observed that downregulated expression of DNA-PKcs alters the transcriptional levels of IL8, IL10RA and DAPK3.^481^ We also found that DNA-PKcs activity is necessary for c-myc protein expression, as DNA-PKcs deficiency suppressed c-myc expression.^482^ Recently, we reported that knockdown of HUWE1-dependent DNA-PKcs neddylation decreased the repair efficacy of the NHEJ pathway.^483^

In clinical cancer therapy, the use of inhibitors of retained DNA repair machinery components is a promising method for eradicating cancer cells. Inhibitors targeting DNA-PKcs include NU7441, nedisertib, AZD7648, VX-984, berzosertib, ceralasertib, VX-803, and BAY1895344.^40^ Among these inhibitors, some have reached the clinical setting. Nedisertib has been approved for phase II trials for various indications.^484^ NU7441, a very strong inhibitor of cancer cell proliferation and growth, shows synergy with enzalutamide against advanced prostate cancer in the clinical setting.^485^ Nedisertib, also known as M3814, can modulate ATP-binding cassette transporter family-mediated multiple resistance in lung cancer chemotherapy.^486^ AZD7648, which is now in phase I trial, has been described as a potent and selective DNA-PKcs inhibitor and is associated with good chemotherapy outcomes and enhanced olaparib activity.^487^ VX-984, also in phase I trial, showed potential for selective inhibition of NHEJ in transformed cells,^488^ and enhanced the sensitivity of glioblastoma cells to radiation therapy.^489^ Berzosertib, which is an ATR inhibitor currently in phase I trial, is considered a safe and effective inhibitor of advanced solid cancers.^490^ In some studies, berzosertib has synergized with gemcitabine to enhance therapeutic outcomes in some serous ovarian cancers including platinum-resistant high-grade serous ovarian cancer. Ceralasertib, also known as AZD6738, showed synergistic activity in combination with olaparib in ATM-deficient cancer cells, and was found to be tolerable and effective for prolonging progression-free survival of patients with platinum-sensitive relapsed epithelial ovarian cancer.^491^ VX-803 and BAY1895344 are also selective ATR inhibitors currently in phase I trials.^492^ Although numerous studies have indicated that inhibition of DNA-PKcs is deleterious to cancer cells, with very promising results, further pre-clinical and clinical trials should be conducted to obtain more substantial data. Meanwhile, the upstream and downstream targets of DNA-PKcs should be explored to support development of additional targeted therapeutic agents for cancer control.

#### Targeting ligases

In 2008, three types of ligases were cloned and their structures, comprised of a catalytic region, nucleotidyltransferase domain and oligonucleotide domain, were identified.^493^ Subsequent functional investigation showed that DNA ligases I and IV have regulatory functions in the nucleus, whereas DNA ligase III is active in both the nucleus and mitochondrion. Ligase I can interact with proliferating cell nuclear antigen to function in the NER, BER and alt-EJ pathways. Ligase III can interact with other proteins including UNG2, APE1, APTX, PCNA, PNKP and Pol β, providing a specific role for ligase II in DNA replication and repair.^494,495^ Notably, DNA ligase III is the sole enzyme active in the mitochondrion. DNA ligase IV can form a complex with XRCC4 during the early stage of DNA DSB repair via NHEJ through binding with Ku70/Ku80 at a DNA end.

In 2019, a new DNA ligase I inhibitor designated S012-1332 was developed that showed in vivo activity against breast cancer.^496^ Previously, the small-molecule inhibitors L82, a ligase I selective inhibitor, and L67, a selective inhibitor of ligases I and III, were reported to function in cancer cells.^497^ Another ligase I inhibitor, benzocoumarin-stilbene hybrid, can reduce cancer cell proliferation and growth, suggesting that it may be a promising chemotherapy agent for cancer through regulation of DNA damage repair pathways.^498^ L189 is an inhibitor of three DNA ligases characterized in 2008, which targets DNA replication and repair.^499^ SCR7, targeting NHEJ, blocks ligase IV-mediated joining rather than that by DNA ligases I and III.^500^ Recently, Ujjayinee R et al. developed a new ligase IV-specific inhibitor, SCR130, which could inhibit NHEJ and induce cancer regression in mice while having minimal or no effect on joining mediated by ligases I and III.^501^ Unexpectedly, the effects of these inhibitors on cancer cell therapy provide evidence to support further development of effective therapeutic strategies.

The rapid progress in understanding the cellular machinery associated with DDR pathways and its roles in various cancers has led to the discovery of new potential targets, including selective and specific DNA repair-related kinase inhibitors. Among these inhibitors, some have been approved for clinical application, while others are currently in the clinical trial stage or being investigated in cell or animal models. In addition, targets on other DNA damage repair kinases, which are not discussed in detail in this section but described in our previously published review, such as ATR, CHK1 and WEE1, are being investigated for clinical application in cancers, making them potential new cancer chemotherapy agents in the future. In addition to the cytotoxicity to cancer cells of these inhibitors, their safety and tolerance should be evaluated carefully for dose-response effects, which may limit their therapeutic application. Notably, combination therapy methods with two or more inhibitors applied together should also be evaluated in terms of potential efficacy, safety and tolerance. Chemotherapy patients often experience off-target toxicity and detrimental side effects due to the impacts of chemotherapy on healthy tissue. Importantly, chemotherapy programs should focus on precision therapy, meaning that therapy with inhibitors associated with DNA damage repair defects should be employed with consideration of the cancer patient’s traits, such as genetic background, environment, lifestyle, diet, culture and race, which are important factors that can affect therapy results.^502^ Finally, chemotherapy resistance is a serious threat to the fight against cancer, and the mechanisms underlying resistance, such as that reported for PARP-1 inhibitor resistance, require further elucidation.

#### Targeting PI-3 kinase (Phosphatidylinositol 3-kinase)

The PI3K family of kinase consists of I, II and III classes. Each class has its specific substrates and potential roles.^503^ Previous studies found that activation of PI3K pathway would initiate a cascade of downstream signals to regulate biological roles including cell proliferation or survival. In brief, PI3K class I can catalyze the conversion of phosphatidylinositol 4,5-bisphosphate(PIP2) to PIP3, leading to the activation of AKT. If the PI3K was activated in an aberrant way, either amplifications or mutations would occur in this signaling pathways-related genes,^504^ resulting in many serious diseases. Approximately 50% breast cancer patients have PI3K pathway-related genes’ mutations.^505^

As of the frequent involvement of the PI3K pathway in various cancers, inhibitors have been developed and are being under surveyed in clinical trials over the past a few decades. These inhibitors include PI3K inhibitors, isoform-specific PI3K inhibitors and dual PI3K/mTOR inhibitors. In terms of the PI3K inhibitors’ anticancer effects, for instance, in breast cancer therapy, most of PI3K inhibitors were reported the modest therapeutic index such as buparlisib. Additional, these inhibitors have been found with mood adverse effects, raising the concerns about the safety and need further investigative efforts in breast cancer therapy.^506^ Till now, several PI3K pathway inhibitors are reported in the clinical trials for various cancers. Copanlisib, an intravenous pan-class I PI3K inhibitor, illustrated improved progression-free survival in patients with relapsed or refractory indolent non-Hodgkin lymphoma compared with placebo plus rituximab in a Phase III trial.^507^ Alpelisib, the first PI3K inhibitor approved by FDA showed had effect on treatment of PIK3CA-mutated, hormone receptor-positive breast cancer.^508^ Buparlisib, a pan-class I PI3K inhibitor had a clinical benefit rate of 12% in a Phase II clinical trial.^509^ Some trials evaluated the combination anticancer effects of multiple drugs, such as combination of buparlisib, taselisib and alpelisib or combination of AZD8186(PI3Kβ/δinhibitor) in treatment of breast cancer^510^ or serabelisib(PI3Kα inhibitor) and copanlisib(PI3Kα and PI3Kδinhibitor) in treatment of relapsed follicular lymphoma.^511^ Furthermore, the safety data from PI3K inhibitor studies should be assessed to reduce potential adverse effects in cancer patients and therefore help maximize cancer therapy outcomes.

### Targeting DNA damage repair pathways to improve cancer radiotherapy responses

Radiotherapy, in which certain doses of ionizing radiation are applied to induce cancer cell death or control growth and proliferation through triggering of DNA DSBs, is a major treatment strategy used for patients with various cancers. Currently, approximately half of cancers require radiotherapy for clinical treatment.^512^ In addition, radiotherapy is often used to shrink the cancer prior to surgery or used following surgery to kill residual cancer cells. Ionizing radiation penetrates into cancer cells and tissues, with direct and indirect destructive functions. Direct function refers to the induction of single-strand breaks and DSBs of DNA, whereas indirect functions include the activation and formation of ROS or free radicals that subsequently damage DNA or alter cellular DNA damage repair pathways. Radiation often destroys DDR pathways in cancer cells, but cancer cells have evolved rescue pathways that allow cancer cells to survive radiotherapy. Therefore, targeting these rescue pathways is expected to be an effective therapeutic approach for reducing cancer recurrence or improving radiotherapy sensitivity.^513,514^ However, radio therapy still faces many challenges. One challenge is that damage to normal tissues and cells and adverse effects due to radiotherapy often occur during radiotherapy treatment. Another challenge is radiotherapy resistance. At present, no radio sensitizers for use in combination neo-chemo- and radiotherapy have been approved for clinical application. Typically, most chemotherapeutic drugs are used in combination with radiotherapy to enhance the cancer’s sensitivity to treatment. In a recently reported phase III trial, avelumab and cetuximab in combination with radiation were tolerated by patients with advanced squamous-cell cancer.^515^ A clinical trial conducted by Charles AK et al. showed that the combination of cisplatin and radiotherapy improved progression-free survival time by three years.^516^ As currently available radiotherapies cause toxicity in normal tissues with adverse effects, it is essential to improve radiotherapy sensitivity through the discovery of new targeted agents, especially these based on the mechanism of DDR pathway regulation. In other words, there is urgent need for improvement of radio-sensitivity to develop and exploit highly effective targeted inhibitors with low toxicity. The review by Amy MB et al. summarized a few key hallmarks of cancer that can be targeted for further improvement of radiotherapy outcomes. These hallmarks of cancer cells include enhanced DDR, inflammation, altered mitochondrial and energy metabolism, apoptosis evasion, repopulation by cancer stem cells, hypoxia, expanded cancer subclones, immune evasion, and alteration of the cell cycle.^512^ Regarding these cancer hallmarks, many issues require elucidation, such as (i) why cancer cells possess so many mechanisms against radiation insult; (ii) which hallmarks have the predominant position or most important functions; (iii) whether the activation of these pathways in cancer cells after irradiation occurs simultaneously or sequentially; (iv) the relationships among these hallmarks; and (v) methods for targeting two or more hallmarks at the same time, In this section, we focus on the targeting of DNA repair pathways as a viable therapeutic method to improve the efficacy of cancer radiotherapy.

#### Targeting non-homologous end joining

Previous studies have been conducted to determine the effects on cancer radiotherapy outcomes of employing NHEJ-related proteins as efficacy targets. DNA-PKcs, a key factor in the NHEJ pathway, was found to improve radiotherapy sensitivity in numerous cell and animal studies investigating the effects of its defect. Using NU7441 to knockdown DNA-PKcs expression enhanced the sensitivity of various cancers to radiotherapy.^517^ For example, in post-irradiation colon cancer cells, an inhibitory peptide BTW3, targeting DNA-PKcs T2647 phosphorylation, enhanced sensitivity to radiation treatment.^518^ In breast cancer, inhibition of DNA-PK with a histone deacetylase inhibitor, TMU-35435, which can degrade DNA-PKcs with proteasomes, enhances radiotherapy sensitivity.^519^ M3814, a pharmacological inhibitor of DNA-PKcs, can potentiate radiotherapy in ovarian cancer animal models and non-small cell lung cancer models.^520^ Thus, among the scientific community, the leading perspective is that DNA-PKcs is a promising therapeutic target for human cancer treatment. However, challenges remain for the treatment of some cancers, such as esophageal cancer, for which potential targeted DNA-PKcs inhibitors have not yet been discovered. In addition to DNA-PKcs inhibitors, recent research has indicated that the expression of APLF, a key protein regulating DNA end excision in NHEJ, increased in radiation-resistant glioblastoma cells, suggesting that it may be a useful novel target for glioblastoma radiotherapy.^521^ Another study found that thymine DNA glycosylase (TDG) can regulate the NHEJ pathway through demethylation of cytosine-guanine (CpG) islands in the TAZ gene, thereby enhancing esophageal cancer cell proliferation and radiation resistance.^522^

#### Targeting homologous recombination

To improve radiotherapy efficacy, it is essential to understand radiotherapy resistance in HR-defect cancers. Compared to the NHEJ repair pathway, cancer cells with rapid replication capacity tend to employ the HR-mediated DSB repair pathway. Thus, understanding how cancer cells respond to radiation through the HR pathway would benefit the development of novel approaches to exploring radiotherapy resistance. Notably, HR repair pathway deficiency has been observed in some cancers.^523,524^ HR-deficiency caused cancer cells to become sensitized to chemo-and radio-therapeutic agents, suggesting that HR deficiency is a valuable pathway to improve radiotherapy resistance.^525^ Some factors influence the formation of HR deficiency including promoter methylation, BRCA1/2 mutations and somatic HR mutations.^526^ Telomeric allelic imbalance (TAI) with breakage sites can result in HR deficiency, and is part of the “genomic scar” induced by HR deficiency.^527^ Mutations of BRCA1/2 are the most common source of HR deficiency. When hyperthermia is used to inhibit the HR pathway, BRCA2-deficient cancer cells become more sensitive to radiation treatment. Combining a PARP inhibitor with hyperthermia showed good treatment efficacy for cancers with HR deficiency.^528^ Gregory et al. showed that YU238259, an inhibitor of DNA DSB repair, has synergistic effects with ionizing radiation and PARP inhibition, and that this synergism is enhanced with BRCA2 deficiency. Thus, this inhibitor has strong potential as either a monotherapy or an adjuvant for radiotherapy. Treatment with the combination of AZD6738, an inhibitor of ATR, and olaparib significantly improved ionizing radiation-induced resistance, suggesting that combination therapy with two or more agents might be an effective approach for treating cancers intrinsically resistant to radiation.^529^

#### Double targeting of HR and NHEJ

A number of inhibitors have been developed to improve radiotherapy resistance, which target both the HR and NHEJ pathways. The MEK1/2 inhibitor, GSK212, also known as trametinib, has shown promising anticancer efficacy and confers radio sensitization to pancreatic adenocarcinoma cells through double suppression of HR and NHEJ.^530^ Additional studies have reported the use of this double targeting approach in other cancers. Cells of squamous cell cancer of the head and neck that were treated with valproic acid, a histone deacetylase inhibitor, enhanced the radiosensitizing effect by increasing radiation-induced DNA DSBs through double targeting of HR and NHEJ.^531,532^ A clinical trial conducted by Mary et al. showed that the combined use of the MEK1/2 inhibitor, selumetinib, with 5-fluorouracil (5-FU) significantly reduced the clonogenic survival time of colorectal cancer cells and increased their radiation sensitivity.^533^ In a phase I study, Christina W et al. reported that the combination of trametinib, 5-FU and chemoradiation treatment was safe and well tolerated in patients with stage II or III rectal cancer.^534^ Meanwhile, Chang et al. found that BEZ235, a dual inhibitor of PI3K and mTOR, greatly improved radiation sensitivity through inactivation of HR and NHEJ proteins in radioresistant prostate cancer cells.^535^ Using a high-throughput cell-based small molecule screening method, Alexander et al. identified a collection of novel compounds that can selectively modulate both the HR and NHEJ repair pathways. Among these compounds, some have been approved by the FDA, such as the calcium channel blocker mibefradil dihydrochloride, and have predicted activities as HR and NHEJ repair inhibitors and radiosensitizers. Moreover, they found that helenine, an antiviral drug, can reduce NHEJ and HR repair activity to 20% and 6%, respectively, suggesting that it could be of great benefit for future clinical use.^536^ With the development of high-throughput technologies, numerous studies have aimed to discover potential inhibitors with activities against both HR and NHEJ, through which many novel compounds have been identified, some of which have reached phase I trials, such as the radiosensitizer mibefradil (Trial# NCT02202993). However, a few major questions remain about combination treatment with two or more therapies or the discovery of targets with dual functions in both the HR and NHEJ pathways. (i) Off-target effects are possible, and their underlying mechanisms are highly complex. Such effects may be associated with mitochondrial alteration or immune responses.^537^ As off-target effects are a major obstacle to the clinical application of combinations of inhibitors, it is essential to reveal the molecular mechanisms of off-target effects. (ii) To effectively enhance radiosensitivity, more attention should be paid to investigations of the safety and tolerance of combinations of inhibitors. (iii) The inherent DNA repair ability of cancer cells is a key factor affecting the efficacy of combination treatment with multiple inhibitors. Thus, identification of potential biomarkers associated with various cancer types or cancer stages, as well as with gender or environmental factors, would aid in the development of optimal radio sensitizers and personalized cancer therapies.

## Clinical therapeutic drugs for DNA damage and repair

Most of cancer therapeutic drugs have been applied in clinic for a few decades with the enough evidence of efficacy in killing proliferating cancer cells(Table 2). Typically, these agents comprise of antimetabolites such as 5-fluorouracil, radiotherapy and radiomimetics such as Bleomycin, Monofunctional alkylators such as Temozolomide, Bifunctional alkylators such as Cisplatin, Topoisomerase inhibitors such as VP16 and Replication inhibitor such as Aphidicolin based on the induced cancer cells’ toxic DNA lesions18256616. In general, the effects of these agents on cancer therapy are affected by a few following factors. (i) DNA replication is one of the targeted period by some of cancer chemotherapy agents. These agents may produce excessive DNA damage resulting in cell death following DNA replication; (ii) cell repair ability can influence the efficacy of cancer therapy agents. For instance, some DNA repair inhibitors have been identified the efficacy in preclinical models (Table 2); (iii) tumor survival microenvironment and development are linked with perturbed DNA damage response and DNA damage repair pathways. Due to this perturbation effect, the DNA repair ability is reduced, leading to the cancer cells’ genomic instability. However, one DNA repair pathway destroyed by agents may be compensated by other alternative pathways. These alternative other compensated pathways can be identified to treat the cancer with DNA repair-defective. Currently, in clinic, the advantages and disadvantages by using single DNA repair agents have been reported. The advantages consist of that single agents could exploit cancer-specific defects in checkpoint signaling and DNA repair. This advantage can convert endogenous DNA lesions into fatal replication lesions in cancer cells resulting in death. Another advantage is the side effects of single DNA repair inhibitors would be minimized through cross-talk among normal cells. However, the most limitation of single agents in treatment of cancer is the acquisition of resistance in cancers. This limitation may be caused by the mechanism of cross-talk among DNA repair pathways.

**Table 2:** Possible therapeutic strategies with DNA damaging agents and DNA damage repair (DDR) inhibitors in cancer therapy

Cancer type	Drug name	Targeted gene	Trial	Therapeutic effects	Reference
Breast cancer	Olaparib	PARP inhibitor	Phase II trial	Olaparib yielded a high clinical response rate for therapy of triple negative breast cancer with HR deficiency	^560^
	Cyclophosphamide	BRCA1/2, PALB2, BLM	Phase II trial	Over a 3-year follow-up, all patients were alive and disease-free survival was 87.1%	^561^
	Carboplatin	platinum compound	Phase II trial	The combination of bevacizumab and carboplatin results in a high rate of durable objective response in patients with brain metastases from breast cancer	^562^
	Talazoparib	PARP inhibitor	Randomized control, phase III	Compared to the physician’s choice of chemotherapy, Talazoparib didn’t improve final overall survival in BRCA-mutated HER2-negative advanced breast cancer	^563^
	Veliparib (ABT-888)		Randomized control, phase III	Veliparib yielded to a higher free survival time than placebo group in BRCA-mutated HER2-negative advanced breast cancer	^564^
	Niraparib	PARP inhibitor	Open-Label Clinical Trial	Combination niraparib plus pembrolizumab provides promising antitumor activity in patients with advanced or metastatic TNBC, with numerically higher response rates in those with tumor BRCA mutations	^565^
	Iniparib	PARP inhibitor	Phase II trial	The addition of iniparib to gemcitabine and carboplatin improved the rate of clinical benefit from 34% to 56% and the rate of overall response from 32% to 52%	^566^
	AZD0156	ATM inhibitor	Phase I study	AZD0156 enhances the tumor growth inhibitory effects of radiation treatment in vivo	^567^
	Prexasertib(LY2606368)	CHK1/2 inhibitor	Phase II trial	Prexasertib monotherapy had modest clinical efficacy in BRCAwt TNBC	^568^
Ovarian cancer	Rucaparib		Randomized control, phase III	Rucaparib yielded a high clinical response rate for therapy of recurrent ovarian cancer with BRCA-mutant cohort, HR deficient cohort	^569^
	Niraparib		Randomized control, phase III	Niraparib yielded a long-term safety for maintenance therapy in patients with recurrent ovarian cancer	^570^
	Cyclophosphamide	BRCA1/2, PALB2, BLM	Phase II nonrandomized trial	Oral cyclophosphamide was well tolerated and demonstrated clinical benefit in 25.0% of patients with recurrent ovarian cancer	^571^
	Olaparib	PARP inhibitor	Randomized control, phase III	Mean quality-adjusted progression-free survival (olaparib 29.75 months [95% CI 28.20–31.63] vs placebo 17.58 [15.05–20.18]	^572^
	Cisplatin	Alkylating agent	phase III	Cisplatin + Doxorubicin does not induce significant hepatic and renal toxicity and can be considered a valid therapeutic option in patients with advanced peritoneal disease	^573^
	Ceralasertib		Randomized control, phase II	Trial registration:NCT04239014	^491^
Bladder cancer	Cisplatin	Alkylating agent	Phase II	Gemcitabine and cisplatin is an active, well-tolerated neoadjuvant regimen for the treatment of patients withmuscle-invasive bladder cancer	^574^
	Mitomycin	Alkylating agent,	Phase II	Intravesical mitomycin C of a short-term schedule is safe and without additional toxicity compared with the weekly regimen	^575^
	Doxorubicin	topoisomerase II inhibitor	Phase III	Treatment with dose-dense methotrexate, vinblastine, doxorubicin, and cisplatin results in a high response rate, less toxicity, and few dosing delays	^576^
	KU55933	ATM inhibitor	Phase I	The ATM inhibitor KU55933 sensitizes radioresistant bladder cancer cells with DAB2IP gene defect	^577^
	CM-272	DNMT1 inhibitor	Phase I	The antitumor effect is significantly improved when CM-272 is combined with anti-programmed cell death ligand 1, even in the absence of cisplatin	^578^
Colon cancer	5-fluorouracil	DSBs inductor	Phase II	Fluorouracil monotherapy combined with panitumumab was safely	^579^
	Irinotecan	Topoisomerase I inhibitor	Phase II	S-1 plus irinotecan and oxaliplatin is feasible and efficacious for refractory mCRC patients	^580^
	Oxaliplatin	Alkylating agent	Phase II	S-1 plus oxaliplatinhas decreased the incidence of grade 2 neuropathyfrom 8.1 to 2.7% after 3 years of the therapyand survival rate was 73.9%	^581^
	Olaparib	PARP inhibitor		The lack of anti-tumor efficacy observed in this trial makes this combination of olaparib and irinotecanlittle interest for further clinical development	^582^
Glioblastoma	Temozolomide	Alkylating agent	Phase 1	Mebendazole at doses up to 200 mg/kg demonstrated long-term safety and acceptable toxicity	^583^
Lung cancer	Gossypol		Randomized control	Gossypol yielded to a better median progression-free survival and median overall survival rate than control placebo group	^584^
	Cisplatin	Alkylating agent	Phase II	Metronomic oral vinorelbine and tri-weekly cisplatin has a 66.2% overall response rate in non-small cell lung cancer	^585^
	Talazoparib	PARP inhibitor	Phase II	Median progression-free survival and overall survival were 2.4 months (95% CI, 1.5–2.8) and 5.2 months (95% CI, 4.0–10), respectively	^586^
	Olaparib	PARP inhibitor	Phase I/II trial	Combination of olaparib and temozolomide has median overall survival was 8.5 months (95% CI, 5.1–11.3)	^587^
Leukemia	Doxorubicin	DSBs inductor	Phase II	Bortezomib, dexamethasone plus doxorubicin has a overall survival of 36.3 (95% CI, 25.6; -) months	^588^
	Vorinostat	HDAC inhibitor	Phase II	Best overall response rate in phase II was 22%	^589^
	Talazoparib	PARP inhibitor		Talazoparib combined with NL101 had a strong synergistic effect in treating AML	^590^
	Prexasertib	CHK1/2 inhibitor		Prexasertibincreases the effectiveness of conventional therapy in B-/T- cell progenitor acute lymphoblastic leukemia	^591^
Liver cancer	Cisplatin	Alkylating agent	Phase I/II	The overall median survival time of combination of 5-fluorouracil, mitoxantrone and cisplatin, is 11.3 months	^592^
	Doxorubicin	Topoisomerase II inhibitor	Phase II	The overall median survival time was 8.6 months	^593^
	Cetuximab		Phase III	Median overall survival was 55·4 months (43.5–71.5) in the chemotherapy plus cetuximab group (HR 1.45, 1.02–2.05; *p* = 0·036) in patients with resectable colorectal liver metastasis	^594^
	Capacitabine	Pro-drug of 5-fluorouracil/TS inhibitor	Phase III	Median overall survival was 51.1 months (95% CI 34.6–59.1) in the capecitabine group compared with 36.4 months (29.7–44.5) in the observation group	^595^
	Mitoxantrone	Topoisomerase II inhibitor	Phase I/II	The overall median survival time of combination of 5-fluorouracil, mitoxantrone and cisplatin, is 11.3 months	^592^
	Veliparib	PARP inhibitor	Phase II	The combination of temozolomide and veliparib is well tolerated in patients with advanced HCC	^596^
	Palbociclib	ATM inhibitor	Phase I/II	Antitumor activity was observed (phase 1). In phase 2, objective responses were achieved in 20% patients	^597^
Non-Hodgkin Lymphoma	Cyclophosphamide	Alkylating agent	Phase II	The overall survival rate was 64% at 10 years	^598^
	Doxorubicin	Topoisomerase inhibitor	Phase II	With a median follow-up of 48·7 months, the 4-year progression-free and overall survivals were 83% and 93%	^599^
	Olaparib	PARP inhibitor		Olaparib is highly radiosensitizing agents when used before external beam radiation and ¹³¹I-tositumomab.	^600^
Prostate Cancer	Carboplatin	Platinum compound	Phase II	Combination of dexamethasone, calcitriol, and carboplatin for patients with HRPC produced a PSA response in 13 of 34 patients and had an acceptable side-effect profile	^601^
	Cisplatin	Alkylating agents	Phase II	Cisplatin plus prednisone appears to represent an active regimen in docetaxel-refractory castration-resistant prostate cancer with an acceptable toxicity profile	^602^
	Nedisertib	DNA-PKcs	Randomized control, phase II	No Study Results Posted on ClinicalTrials.gov for this Study	https://clinicaltrials.gov/
Pancreas cancer	5-fluorouracil	DSBs inductor	Phase I/II	The combination of veliparib and Oxaliplatin was tolerable, the objective response rate overall was 26%	^603^
	Oxaliplatin	Platinum compound	Phase II	Median overall survival, progression-free survival, and response rates were 7.6 months (95% CI 6.0–10.7), 4.0 months (95% CI 2.0–4.6), and 26.7% (95% CI 14.6–41.9), respectively	^604^
	Irinotecan	Topoisomerase I inhibitor		Estimated one-year overall survival rates were 26% with Liposomal irinotecan plus 5-fluorouracil and leucovorin	^605^
	Olaparib	PARP inhibitor	Phase I	Olaparib 100 mg b.i.d. (intermittent dosing; capsules) plus gemcitabine 600 mg/m(2) is tolerated in advanced solid tumor patients, with no unmanageable/unexpected toxicities	^606^

These influence factors make the strategies using DNA repair inhibitors in cancer treatment had some specific properties. First, in clinic, these inhibitors can be combined with DNA damage anticancer drugs to increase the cancer therapy outcomes because combination usage can inhibit DNA-repair-associated removal of toxic DNA lesions. Second, DNA repair inhibitors could be adopted to kill tumor cells in a way of monotherapy or selectively either in DNA damage-defective response or DNArepair. Furthermore, the synthetic lethal interactions within the defective cancer and DNA repair pathway could be served as identification of new therapy strategies. Third, we can further consider that the DNA repair inhibitors would be served to promote cancer-associated replication lesions to kill them in a selective way.

Theoretically, DNA repair inhibitors are used to kill the cancer cells with replication lesions and convert them into fatal replication lesions, thus the cancer cells present to be killed specifically. Thus, in the future, it can be proposed that DNArepair inhibitors should be developed to make replication lesions more toxic, leading to more fatal replication lesions selectively killing oncogene-expressing cancer cells. Actually, our understanding regarding a few replication repair pathways remains limited, in particular, their complicated interplay reaction. Thus, more extensive basic experiments and study are needed to explore more and more new anticancer targets in this field.

## Hypothesis: “DNA damage baseline drift”-mediated repair may be magnified in cancer therapy

Based on the interpretation of DNA damage, response and repair, and their associations with cancer therapies described above, we formulated a new hypothesis to provide new insights into the functions of DDR in cancer therapy. This hypothesis states that “DNA damage baseline drift”-mediated repair may be magnified in cancer therapy.

First, we must define “DNA damage baseline drift”. Compared to normal cells, cancer cells undergo a process known as carcinogenesis, in which DNA damage leads to a series of genetic mutations and finally to formation of a mass of cells, finally grows into tumors known as tumorigenesis.^538^ This process, in which normal cells become cancer cells, includes two critical periods: the benign accumulation period and malignant period.^539,540^ Because DNA can be repaired after damage, during the initial period of environmental hazards or endogenous insults, the DNA repair system maintains genomic stability; however, as exposure to insults continues, long-term exposure may cause cancer, a state of abnormal and uncontrollably rapid growth. During these two vital periods, the cells experience the following stages of cancer cell transformation: exposure to a carcinogen, initiation of DNA damage, enhancement of the mutated cell, growth, and replication. This process of DNA damage occurs in normal cells after insult from carcinogens, and has shown properties of dose-response and time-response in numerous studies.^541,542^ In accordance with this background, we formulated the new concept of a baseline level of DNA damage repair ability in normal cells that become tumor cells. Below this baseline level, normal cells can repair their DNA damage and maintain genomic stability, whereas beyond this baseline level, DNA damage repair becomes unable to reverse the carcinogenetic effects effectively, or DNA damage repair shifts to supporting the proliferation and growth of tumor cells. As shown in Fig. 8, under the effects of continuous exogenous and endogenous carcinogen insults, one or a few normal cells cannot tolerate the stress, leading to DNA damage baseline drift, which results in the hallmarks of cancer cells, such as genomic instability, genetic mutations, enhanced growth, altered cellular energy metabolism, inflammation, and promotion of invasion and metastasis. There could be an exceptional situation, i.e., the repair pathway is chosen inappropriately, leading to error-repair or “non-fidelity repair”, which may also have the potential of carcinogenesis even at the baseline level.

The second significant aspect of this hypothesis is that “DNA damage baseline drift”-mediated repair could be magnified in cancer therapy. Here, the term magnification refers to the multiple possible roles of DNA damage repair. During the early stage of cancer immuno-, chemo-, and radio-therapy, DNA damage repair-related proteins or complexes could be targets for improving therapy efficacy; however, as time goes on, tumor cells may initiate specific DNA damage repair machinery and thus develop resistance to therapy. For this reason, therapy-resistant cancer cells can carry out many new functions, as discussed in the previous section. These therapy-resistant functions and phenotypes include evasion of immune monitoring, alteration of the cell cycle, activation of inflammation-related cytokines, dysregulation of mitochondrial and energy metabolism and evasion of apoptosis. This magnification effect acts like a lever mechanism (Fig. 8), meaning that DNA damage repair can follow specific repair pathways to shift the phenotype of cancer cells toward treatment resistance. Evidence of this magnification effect has been provided in some published studies. Matthew JS et al. demonstrated that PARP-1, a key protein for DNA damage repair, plays dual roles in the regulation of cancer growth and progression.^543^ The review by Carol B et al. summarized a number of DNA damage repair-related proteins with dual roles, including BRCA1, ATM, ATR and p53. p53 is a classical example of a gene with dual roles in cancer development. p53 is the most frequently mutated gene in human cancers.^544,545^ Furthermore, in some cell types, p53 can enhance apoptosis, whereas in other cell types, p53 intensifies cell cycle progression.^546^ p53 inactivation leads to intratumoral heterogeneity and, compared to wild-typep53, mutant p53 may exhibit gain-of-function mutations related to apoptosis, anti-oxidant activity or the epithelial-to-mesenchymal transition (EMT) in addition to its DNA damage repair functions. According to Qing S et al., the effects of combination therapy with a DNA-PK inhibitor, M3814, and radiation were more dependent on p53 regulation of cell-cycle blockade and senescence, whereas knockout of p53 expression led cancer cells to initiate hallmarks events such as mitotic catastrophe and apoptotic cell death.^547^ In cells of human head and neck squamous cell cancer exhibiting downregulation of the MRN complex, the accumulation of lethal DNA damage in the form of DSBs and telomere length shortening contributed to the death of BRCA-proficient cancer cells.^548^ Theoretically, DNA damage baseline drift may suggest the reason why cancer cells gain so many functions, as doing so would avoid further damage from cancer treatment. Cancer therapy-induced resistance may also be subject to magnification effects caused by the continuous accumulation of DNA damage repair sites.

The purpose and significance of raising this hypothesis relates to the following aspects of cancer biology and treatment: i) Addressing DNA damage caused by environmental hazards and endogenous toxicant insults, which induce accumulation of errors in a time- and dose-dependent manner. DNA damage repair capacity depends on numerous factors including cell background, exposure time and dose, as well as genetic and epigenetic factors. These factors indicate that DNA damage in excess of the exposure repair threshold, or in other words, DNA damage accumulation at a level that cannot be repaired, leads to a cascade of changes in cancer- and cancer therapy-related phenotypes. ii) Providing a novel perspective for clarifying further DNA damage repair functions during cancer development and their impacts on cancer therapy outcomes. This novel perspective may pave the way for establishment of new study fields or new technologies to explore DNA damage repair in a systematic manner. iii) Providing new prospective cancer therapeutics for future clinical application, as a small investment in understanding DNA damage repair could yield enormous returns. Better understanding of this process would improve the future prospects of personalized precise cancer therapy.

## Current challenges and future perspectives

**Fig. 9:**
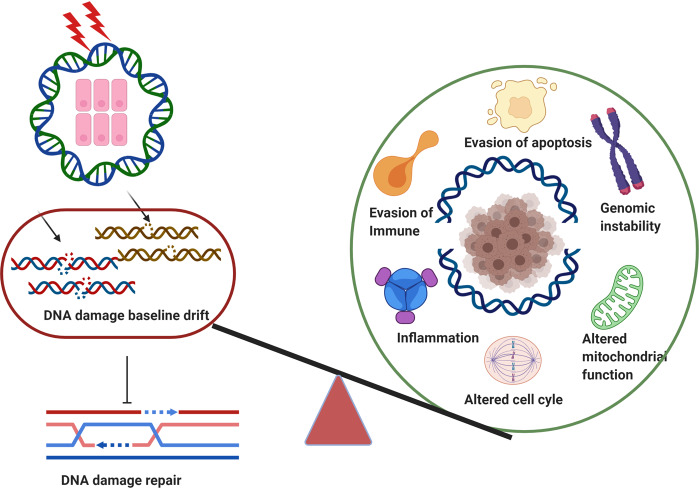
Hypothesis of “DNA damage baseline drift”. Compared to normal cells, cancer cells undergo a process known as carcinogenesis, in which DNA damage leads to a series of genetic mutations and finally to formation of a mass of cells, finally grows into tumors known as tumorigenesis

DNA damage, response and repair have garnered the attention of cancer researchers, physicians and surgeons, while intensive research has produced new fundamental insights into the mechanisms underlying cancer development and cancer therapy-induced resistance. In addition, the development of new potential inhibitors to improve cancer therapy would benefit from research on DNA damage, response and repair. This review highlights the roles of DNA damage, response and repair in cancer development and cancer therapy from perspectives of the historical research timeline and clinical applications. Based on this background, we suggest two hypotheses, namely “environmental gear selection” to describe DNA damage repair pathway evolution, and “DNA damage baseline drift”, which may play a magnified role in mediating repair during cancer treatment (Fig. 9). This “DNA damage baseline drift” hypothesis may provide a novel holistic concept at the genetic level in the area of DNA damage repair and cancer therapy and care for serious conditions such as chemotherapy resistance and radiotherapy resistance, etc.

Genomic stability is important for cellular survival and evolution, and cells respond to environmental hazards and endogenous stresses through complex interactions between DNA damage-related sensors, activators, repair pathways, and protein complexes, with additional effects from cellular context and status. While this complex network underlies the molecular mechanisms of DDR and repair-mediated cancer therapy, it also provides a resource for identifying potential inhibitors in a systematic manner. However, the plethora of DNA damage, response and repair processes, along with their profound and complex interactions, have not yet been fully elucidated. Similarly, the advent of personalized cancer therapy and high-throughput sequencing technologies provide hope for future prediction of many gene alterations associated with specific conditions. Thus, despite decades of extensive research and countless discoveries, much more work is needed to appreciate fully the roles of DNA damage, response, repair and their regulation in cancer and cancer treatment.

Challenge I: Considering that DNA damage, response, and repair are critical to cancer therapy, it is reasonable to recategorize cancers from the perspective of their DNA repair deficiency status. This new categorization could provide a new perspective for the development of personalized cancer therapies. For example, cancer patients with DNA repair defect in the NHEJ pathway could be treated with therapy inhibitors specific to the HR pathway. This process is achievable due to the rapid development of whole-genome cancer sequence detection, and supports exploitation of all relevant alterations and mutations for cancer therapy.

Challenge II: Early biomarkers for identifying DNA damage, response, and repair defects should be identified and used for cancer therapy selection. Although many relevant biomarkers have been reported in recent decades, most require improvement. For example, deficiency of RAD51 foci has been used as a biomarker for the detection of DNA repair ability via immunohistochemistry. However, as the test method is complex and the results may be affected by many factors, this test has not been widely used in clinics. Moreover, in the era of personalized cancer therapy, a greater number of potential functional markers and those that reflect early changes should be identified for the DNA damage, response and repair process, along with the development of more precise experimental methods. In addition, an ethical issue remains to be addressed. In the real world, to test patient responses to cancer therapy, patients should be tested for potential DNA damage, response and repair defects using these early changing biomarkers, but these biomarkers must be activated using activators such as radiation, chemotherapy or immunotherapy, which would be very difficult for ethical reasons. Therefore, prior to the clinical application of biomarkers, ethical issues should be addressed.

Challenge III: The mechanism underlying the activity of DNA damage repair inhibitors in cancer therapy remains unclear, although a number of potential inhibitors have been approved for clinical trials. Because their mechanisms have not been fully revealed, targeted therapies for cancer may have off-target effects. If we can solve this problem, targeted cancer therapy based on exploitation of DNA damage repair can be expected to improve therapeutic outcomes in the future. Recently, the combined usage of two or more inhibitors or therapy methods has increased in popularity. Combined treatment may increase the efficacy of a therapy, but can also enhance toxicity or adverse effects, as its molecular mechanisms are much more complex and difficult to elucidate than those of single treatments. Therefore, the creation of usage criteria and principles for combination treatment to ensure that such therapies are more effective against cancer and less harmful to health is urgently needed.

Challenge IV: Cancer resistance and normal tissue severe side-effects are the major obstacles to cancer therapy, the goal of personalized therapy strategy is to overcome these obstacles. Many resistance mechanisms have been reported to chemo-, radio-, and immuno-therapy. For example, cancer cells with mutated BRCA1 and BRCA2 may be sensitive to chemotherapy using a PARP inhibitor, while the normal tissue cells if without the germling mutation of BRCA1/2 are relatively resistance as compared to the mutated cancer cells. Obviously, in the clinical setting, testing for gene mutation seven the secondary mutations in the recurrence tumors should be popularized and, most importantly, resistance mechanisms should be explored through cell, animal and clinical experiments in the near future.

Challenge V: Distilling the convergent findings obtained from the enormous amount of complicated research conducted on the relationships of DNA damage, response, and repair processes with cancer therapy remains a challenge, as does translating these basic research outcomes into clinical applications. At present, a plethora of inhibitorsare in clinical trials or approved for clinical use that originated from basic cell and animal experiments. Additional biomarkers and agents show promise at the preclinical level, but their translation to the clinical setting has failed for many reasons, such as not providing superior therapeutic outcomes and serious adverse effects. To achieve the purpose of translation study in the clinic in the future, more basic molecular mechanism of DNA damage and repair in cancer therapy should be extensive studied.

In conclusion, we believe that comprehensive research into the basic biology of DNA damage, response, and repair, accompanied by rapid development of new technologies and further progress in targeted cancer therapy, will drive significant advances in the near future. Hopefully, more robust clinical trial results will also be achieved.

## References

[1] Pederson T (2020). The double helix: "Photo 51" revisited. FASEB J..

[2] Watson JD, Crick FH (1953). Molecular structure of nucleic acids; a structure for deoxyribose nucleic acid. Nature.

[3] da Costa, A. & Baiocchi, G. Genomic profiling of platinum-resistant ovarian cancer: The road into druggable targets. *Semin. Cancer Biol*. **5**, 30221–30222 (2020). 10.1016/j.semcancer.2020.10.016 33161141

[4] Sengupta D, Mukhopadhyay A, Sengupta K (2020). Emerging roles of lamins and DNA damage repair mechanisms in ovarian cancer. Biochem Soc. Trans..

[5] Parekh, V. J. et al. Crucial Role of the C-Terminal Domain of Hfq Protein in Genomic Instability. *Microorganisms*. **8**, 1598 (2020). 10.3390/microorganisms8101598 PMC7603069 33080799

[6] Huang RX, Zhou PK (2020). DNA damage response signaling pathways and targets for radiotherapy sensitization in cancer. Signal Transduct. Target Ther..

[7] Gager CS, Blakeslee AF (1927). Chromosome and gene mutations in datura following exposure to radium rays. Proc. Natl Acad. Sci. USA.

[8] Vasil IK (2008). A history of plant biotechnology: from the Cell Theory of Schleiden and Schwann to biotech crops. Plant Cell Rep..

[9] Muller HJ (1928). The production of mutations by X-Rays. Proc. Natl Acad. Sci. Usa..

[10] Muller HJ (1927). Artificial transmutation of the gene. Science.

[11] Portin P (2014). The birth and development of the DNA theory of inheritance: sixty years since the discovery of the structure of DNA. J. Genet..

[12] Pettijohn D, Hanawalt P (1964). Evidence for repair-replication of ultraviolet damaged DNA in bacteria. J. Mol. Biol..

[13] Schuster RC (1964). Dark repair of ultraviolet injury in E. Coli during deprivation of thymine. Nature.

[14] Boyce RP, Howard-Flanders P (1964). Release of ultraviolet light-induced thymine dimers from DNA in E. Coli K-12. Proc. Natl Acad. Sci. USA.

[15] Setlow RB, Carrier WL (1964). The disappearance of thymine dimers from DNA: an error-correcting mechanism. Proc. Natl Acad. Sci. USA.

[16] Setlow RB, Regan JD, German J, Carrier WL (1969). Evidence that xeroderma pigmentosum cells do not perform the first step in the repair of ultraviolet damage to their DNA. Proc. Natl Acad. Sci. USA.

[17] Cleaver JE (1968). Defective repair replication of DNA in xeroderma pigmentosum. Nature.

[18] Chiche JD, Cariou A, Mira JP (2002). Bench-to-bedside review: fulfilling promises of the Human Genome Project. Crit. Care.

[19] Kelavkar U, Shah K (1998). Advances in the human genome project. A review. Mol. Biol. Rep..

[20] Harrow J (2012). GENCODE: the reference human genome annotation for The ENCODE Project. Genome Res..

[21] Guigo R (2006). EGASP: the human ENCODE genome annotation assessment project. Genome Biol..

[22] Dolinnaya NG, Ogloblina AM, Yakubovskaya MG (2016). Structure, properties, and biological relevance of the DNA and RNA G-quadruplexes: overview 50 years after their discovery. Biochem. (Mosc.).

[23] Silverman M (2003). The discovery of DNA structure: 50 years on. Clin. Invest Med..

[24] Fu H, Baris A, Aladjem MI (2018). Replication timing and nuclear structure. Curr. Opin. Cell Biol..

[25] Kraus WL (2015). PARPs and ADP -Ribosylation: 50 Years and Counting. Mol. Cell..

[26] Doll R (1975). Pott and the path to prevention. Arch. Geschwulstforsch..

[27] Harris H (2008). Concerning the origin of malignant tumours by Theodor Boveri. Translated and annotated by Henry Harris. Preface. J. Cell Sci..

[28] Wunderlich V (2011). ["He corrects my view and develops it further." Comments by David von Hansemann on the monograph by Theodor Boveri Concerning the origin of malignant tumors (1914)]. Ber. Wiss..

[29] Svoboda J (2008). [Rediscovered Theodor Boveri and his dateless reflections on the development of malignancies]. Cas. Lek. Cesk..

[30] Freitas, M. O., Gartner, J., Rangel-Pozzo, A. & Mai, S. Genomic Instability in Circulating Tumor Cells. *Cancers (Basel)*. **12**, 3001 (2020). 10.3390/cancers12103001 PMC7602879 33081135

[31] Oster, S. & Aqeilan, R. I. Programmed DNA damage and physiological DSBs: mapping, biological significance and perturbations in disease states. *Cells*. **9**, 1870 (2020). 10.3390/cells9081870 PMC7463922 32785139

[32] Matsuda S (2020). Role of tumor suppressor molecules in genomic perturbations and damaged DNA repair involved in the pathogenesis of cancer and neurodegeneration (Review). Biomed. Rep..

[33] Young RF (1945). A clinical review of cancer of the breast and antecedent chronic conditions. Edinb. Med J..

[34] On the Formation and Extension of Cancer-Cells in the Neighbourhood of Cancer, and Their Importance in the Performance of an Operation. *Br Foreign Med Chir Rev*. **15**, 390–400, (1855). PMC5184367 30164369

[35] Bolzan AD (2020). Using telomeric chromosomal aberrations to evaluate clastogen-induced genomic instability in mammalian cells. Chromosome Res..

[36] Luijten M (2020). Utility of a next generation framework for assessment of genomic damage: A case study using the industrial chemical benzene. Environ. Mol. Mutagen..

[37] Owiti, N. A., Nagel, Z. D. & Engelward, B. P. Fluorescence Sheds Light on DNA Damage, DNA Repair, and Mutations. *Trends Cancer*, 7, 240–248 (2020). 10.1016/j.trecan.2020.10.006 PMC7889721 33203608

[38] Legator MS, Flamm WG (1973). Environmental mutagenesis and repair. Annu. Rev. Biochem.

[39] Popescu NC (2000). Comprehensive genetic analysis of cancer cells. J. Cell Mol. Med..

[40] Wengner, A. M., Scholz, A. & Haendler, B. Targeting DNA damage response in prostate and breast cancer. *Int. J. Mol. Sci*. **21**, 8273 (2020). 10.3390/ijms21218273 PMC7663807 33158305

[41] Schneeweis, C. et al. The SUMO pathway in pancreatic cancer: insights and inhibition. *Br J Cancer*, 124, 531–538 (2020). 10.1038/s41416-020-01119-6 PMC7851129 33071285

[42] Dang, F., Nie, L. & Wei, W. Ubiquitin signaling in cell cycle control and tumorigenesis. *Cell Death Differ*. **28**, 427–438 (2020). 10.1038/s41418-020-00648-0 PMC7862229 33130827

[43] Hartwell LH, Kastan MB (1994). Cell cycle control and cancer. Science.

[44] Mota MBS, Carvalho MA, Monteiro ANA, Mesquita RD (2019). DNA damage response and repair in perspective: Aedes aegypti, Drosophila melanogaster and Homo sapiens. Parasit. Vectors.

[45] Jing X (2019). Role of hypoxia in cancer therapy by regulating the tumor microenvironment. Mol. Cancer.

[46] Stewart BW (1994). Mechanisms of apoptosis: integration of genetic, biochemical, and cellular indicators. J. Natl Cancer Inst..

[47] Mani C, Reddy PH, Palle K (2020). DNA repair fidelity in stem cell maintenance, health, and disease. Biochim Biophys. Acta Mol. Basis Dis..

[48] O’Connor PM, Kohn KW (1992). A fundamental role for cell cycle regulation in the chemosensitivity of cancer cells?. Semin Cancer Biol..

[49] Lee CY (2012). High-throughput screening for genes that prevent excess DNA replication in human cells and for molecules that inhibit them. Methods.

[50] Charcosset JY, Soues S, Laval F (1993). [Poisons of DNA topoisomerases I and II]. Bull. Cancer.

[51] Cummings J, Smyth JF (1993). DNA topoisomerase I and II as targets for rational design of new anticancer drugs. Ann. Oncol..

[52] Lawley PD, Brookes P (1968). Cytotoxicity of alkylating agents towards sensitive and resistant strains of Escherichia coli in relation to extent and mode of alkylation of cellular macromolecules and repair of alkylation lesions in deoxyribonucleic acids. Biochem J..

[53] Lawley PD, Brookes P (1967). Interstrand cross-linking of DNA by difunctional alkylating agents. J. Mol. Biol..

[54] Brookes P, Lawley PD (1961). The reaction of mono- and di-functional alkylating agents with nucleic acids. Biochem J..

[55] Brookes P, Lawley PD (1960). The reaction of mustard gas with nucleic acids in vitro and in vivo. Biochem J..

[56] Valles GJ, Bezsonova I, Woodgate R, Ashton NW (2020). USP7 is a master regulator of genome stability. Front Cell Dev. Biol..

[57] Burgess JT (2020). The therapeutic potential of DNA damage repair pathways and genomic stability in lung cancer. Front Oncol..

[58] Lasolle H (2020). Chromosomal instability in the prediction of pituitary neuroendocrine tumors prognosis. Acta Neuropathol. Commun..

[59] Ma Q (2020). Increased chromosomal instability characterizes metastatic renal cell carcinoma. Transl. Oncol..

[60] Varella-Garcia M (2010). Chromosomal and genomic changes in lung cancer. Cell Adh Migr..

[61] Tosato V (2012). Warburg effect and translocation-induced genomic instability: two yeast models for cancer cells. Front Oncol..

[62] Huang R (2020). Integrated analysis of transcriptomic and metabolomic profiling reveal the p53 associated pathways underlying the response to ionizing radiation in HBE cells. Cell Biosci..

[63] Dai X (2020). A novel miR-0308-3p revealed by miRNA-seq of HBV-positive hepatocellular carcinoma suppresses cell proliferation and promotes G1/S arrest by targeting double CDK6/Cyclin D1 genes. Cell Biosci..

[64] Polo SE, Jackson SP (2011). Dynamics of DNA damage response proteins at DNA breaks: a focus on protein modifications. Genes Dev..

[65] Hoeijmakers JH (2001). Genome maintenance mechanisms for preventing cancer. Nature.

[66] Blackford AN, Jackson SP (2017). ATM, ATR, and DNA-PK: the trinity at the heart of the DNA damage response. Mol. Cell.

[67] Mulder MPC, Witting KF, Ovaa H (2020). Cracking the ubiquitin code: the ubiquitin toolbox. Curr. Issues Mol. Biol..

[68] Negrini S, Gorgoulis VG, Halazonetis TD (2010). Genomic instability-an evolving hallmark of cancer. Nat. Rev. Mol. Cell Biol..

[69] Yoon DS (2002). Variable levels of chromosomal instability and mitotic spindle checkpoint defects in breast cancer. Am. J. Pathol..

[70] Brockelmann, P. J., de Jong, M. R. W. & Jachimowicz, R. D. Targeting DNA repair, cell cycle, and tumor microenvironment in B cell lymphoma. *Cells*. **9**, 2287 (2020). 10.3390/cells9102287 PMC7602196 33066395

[71] Kottemann MC, Smogorzewska A (2013). Fanconi anaemia and the repair of Watson and Crick DNA crosslinks. Nature.

[72] Mareckova A (2019). ATM and TP53 mutations show mutual exclusivity but distinct clinical impact in mantle cell lymphoma patients. Leuk. Lymphoma.

[73] Choi M, Kipps T, Kurzrock R (2016). ATM mutations in cancer: therapeutic implications. Mol. Cancer Ther..

[74] Jiang H (2005). Ubiquitylation of RAG-2 by Skp2-SCF links destruction of the V(D)J recombinase to the cell cycle. Mol. Cell..

[75] Huang R (2020). BECN1 promotes radiation-induced G2/M arrest through regulation CDK1 activity: a potential role for autophagy in G2/M checkpoint. Cell Death Discov..

[76] Zhang J (2020). Suppression of LINC00460 mediated the sensitization of HCT116 cells to ionizing radiation by inhibiting epithelial-mesenchymal transition. Toxicol. Res (Camb.)..

[77] Basak, D., Uddin, M. N. & Hancock, J. The role of oxidative stress and its counteractive utility in colorectal cancer (CRC). *Cancers (Basel)*. **12**, 3336 (2020). 10.3390/cancers12113336 PMC7698080 33187272

[78] Canto-Santos, J., Grau-Junyent, J. M. & Garrabou, G. The impact of mitochondrial deficiencies in neuromuscular diseases. *Antioxidants (Basel)*. **9**, 964 (2020). 10.3390/antiox9100964 PMC7600520 33050147

[79] Tubbs A, Nussenzweig A (2017). Endogenous DNA damage as a source of genomic instability in cancer. Cell.

[80] Lin L, Cheng X, Yin D (2020). Aberrant DNA methylation in esophageal squamous cell carcinoma: biological and clinical implications. Front Oncol..

[81] Patel, S. M., Dash, R. C. & Hadden, M. K. Translesion synthesis inhibitors as a new class of cancer chemotherapeutics. *Expert Opin. Investig. Drugs* **30**, 1–12, (2020). 10.1080/13543784.2021.1850692 PMC7832080 33179552

[82] Casati, P. & Gomez, M. S. Chromatin dynamics during DNA damage and repair in plants: new roles for old players. *J. Exp. Bot.* **72**, 4119–4131 (2020). 10.1093/jxb/eraa551 33206978

[83] Klintman, J. et al. Genomic and transcriptomic correlates of Richter’s transformation in Chronic Lymphocytic Leukemia. *Blood*. **137**, 2800–2816 (2020). 10.1182/blood.2020005650 PMC8163497 33206936

[84] Ragunathan, K., Upfold, N. L. E. & Oksenych, V. Interaction between fibroblasts and immune cells following DNA damage induced by ionizing radiation. *Int. J. Mol. Sci*. **21**, (2020). 10.3390/ijms21228635 PMC7696681 33207781

[85] Marshall, C. J. & Santangelo, T. J. Archaeal DNA repair mechanisms. *Biomolecules*. **10**, 8635 (2020). 10.3390/biom10111472 PMC7690668 33113933

[86] Maremonti E (2020). Ionizing radiation, genotoxic stress, and mitochondrial DNA copy-number variation in Caenorhabditis elegans: droplet digital PCR analysis. Mutat. Res..

[87] Pariset, E., Malkani, S., Cekanaviciute, E. & Costes, S. V. Ionizing radiation-induced risks to the central nervous system and countermeasures in cellular and rodent models. *Int. J. Radiat. Biol.* **20**, 1–19, (2020). 10.1080/09553002.2020.1820598 32946305

[88] Wu R (2020). Crystalline silica particles cause rapid NLRP3-dependent mitochondrial depolarization and DNA damage in airway epithelial cells. Part Fibre Toxicol..

[89] Dussert, F. et al. Toxicity to RAW264.7 macrophages of silica nanoparticles and the E551 food additive, in combination with genotoxic agents. *Nanomaterials (Basel)*. **10**, 1418 (2020). 10.3390/nano10071418 PMC7408573 32708108

[90] Huang R, Yu T, Li Y, Hu J (2018). Upregulated has-miR-4516 as a potential biomarker for early diagnosis of dust-induced pulmonary fibrosis in patients with pneumoconiosis. Toxicol. Res (Camb.).

[91] Gupta N, Khetan D, Chaudhary R, Shukla JS (2020). Prospective cohort study to assess the effect of storage duration, leuko-filtration, and gamma irradiation on cell-free DNA in red cell components. Transfus. Med. Hemother.

[92] Fu, J. et al. Epigenetic modification and a role for the E3 ligase RNF40 in cancer development and metastasis. *Oncogene* **40**, 465–474 (2020). 10.1038/s41388-020-01556-w PMC7819849 33199825

[93] Tirman, S. et al. PRIMPOL ready, set, reprime! *Crit. Rev. Biochem. Mol. Biol.* **56**, 1–14 (2020). 10.1080/10409238.2020.1841089 PMC7906090 33179522

[94] Sharma D (2020). DNA binding and antiradical potential of ethyl pyruvate: Key to the DNA radioprotection. Chem. Biol. Interact..

[95] Verhoven BM (2020). Significant improvement in rat kidney cold storage using UW organ preservation solution supplemented with the immediate-acting PrC-210 free radical scavenger. Transpl. Direct.

[96] Chatterjee N, Walker GC (2017). Mechanisms of DNA damage, repair, and mutagenesis. Environ. Mol. Mutagen.

[97] Ceccaldi R, Rondinelli B, D’Andrea AD (2016). Repair pathway choices and consequences at the double-strand break. Trends Cell Biol..

[98] Frankenberg-Schwager M (1990). Induction, repair and biological relevance of radiation-induced DNA lesions in eukaryotic cells. Radiat. Environ. Biophys..

[99] Li J (2019). Pathways and assays for DNA double-strand break repair by homologous recombination. Acta Biochim Biophys. Sin. (Shanghai)..

[100] Lindahl T, Barnes DE (2000). Repair of endogenous DNA damage. Cold Spring Harb. Symp. Quant. Biol..

[101] Evans MD, Dizdaroglu M, Cooke MS (2004). Oxidative DNA damage and disease: induction, repair and significance. Mutat. Res.

[102] Slupphaug G, Kavli B, Krokan HE (2003). The interacting pathways for prevention and repair of oxidative DNA damage. Mutat. Res..

[103] Dizdaroglu M, Jaruga P (2012). Mechanisms of free radical-induced damage to DNA. Free Radic. Res.

[104] Rao KS (2009). Free radical induced oxidative damage to DNA: relation to brain aging and neurological disorders. Indian J. Biochem Biophys..

[105] Ide H, Nakano T, Salem AMH, Shoulkamy MI (2018). DNA-protein cross-links: formidable challenges to maintaining genome integrity. DNA Repair (Amst.).

[106] Tretyakova NY, Groehler AT, Ji S (2015). DNA-protein cross-links: formation, structural identities, and biological outcomes. Acc. Chem. Res..

[107] Stingele J, Bellelli R, Boulton SJ (2017). Mechanisms of DNA-protein crosslink repair. Nat. Rev. Mol. Cell Biol..

[108] Nakano T (2017). Radiation-induced DNA-protein cross-links: Mechanisms and biological significance. Free Radic. Biol. Med..

[109] Zheng, Y. & Sanche, L. Clustered DNA Damages induced by 0.5 to 30 eV Electrons. *Int. J. Mol. Sci*. **20**, 3749 (2019). 10.3390/ijms20153749 PMC6695612 31370253

[110] Eccles LJ, O’Neill P, Lomax ME (2011). Delayed repair of radiation induced clustered DNA damage: friend or foe?. Mutat. Res..

[111] Sage E, Harrison L (2011). Clustered DNA lesion repair in eukaryotes: relevance to mutagenesis and cell survival. Mutat. Res.

[112] Nickoloff, J. A., Sharma, N. & Taylor, L. Clustered DNA double-strand breaks: biological effects and relevance to cancer radiotherapy. *Genes (Basel)*. **11**, JanPMC7017136 (2020). 10.3390/genes11010099 PMC7017136 31952359

[113] De Bont R, van Larebeke N (2004). Endogenous DNA damage in humans: a review of quantitative data. Mutagenesis.

[114] Sangaletti S (2020). Intra-tumour heterogeneity of diffuse large B-cell lymphoma involves the induction of diversified stroma-tumour interfaces. EBioMedicine.

[115] Naval-Sanchez M (2020). Selection signatures in tropical cattle are enriched for promoter and coding regions and reveal missense mutations in the damage response gene HELB. Genet Sel. Evol..

[116] Alhmoud, J. F., Woolley, J. F., Al Moustafa, A. E. & Malki, M. I. DNA damage/repair management in cancers. *Cancers (Basel)*. **12**, 1050 (2020). 10.3390/cancers12041050 PMC7226105 32340362

[117] Pilie PG, Tang C, Mills GB, Yap TA (2019). State-of-the-art strategies for targeting the DNA damage response in cancer. Nat. Rev. Clin. Oncol..

[118] Kitagishi Y, Kobayashi M, Matsuda S (2013). Defective DNA repair systems and the development of breast and prostate cancer (review). Int J. Oncol..

[119] Ijsselsteijn R, Jansen JG, de Wind N (2020). DNA mismatch repair-dependent DNA damage responses and cancer. DNA Repair (Amst.).

[120] Gupta D, Heinen CD (2019). The mismatch repair-dependent DNA damage response: mechanisms and implications. DNA Repair (Amst.).

[121] Nik-Zainal S (2016). Landscape of somatic mutations in 560 breast cancer whole-genome sequences. Nature.

[122] Okoyo C (2020). Prevalence, intensity and associated risk factors of soil-transmitted helminth and schistosome infections in Kenya: Impact assessment after five rounds of mass drug administration in Kenya. PLoS Negl. Trop. Dis..

[123] Collins PL (2020). DNA double-strand breaks induce H2Ax phosphorylation domains in a contact-dependent manner. Nat. Commun..

[124] Podhorecka, M., Skladanowski, A. & Bozko, P. H2AX phosphorylation: its role in DNA damage response and cancer therapy. *J. Nucleic Acids*. **2010**, 920161 (2010). 10.4061/2010/920161 PMC2929501 20811597

[125] Chen KH (2019). Analysis of DNA damage responses after boric acid-mediated boron neutron capture therapy in hepatocellular carcinoma. Anticancer Res..

[126] Monteiro FL (2014). Expression and functionality of histone H2A variants in cancer. Oncotarget.

[127] Baptistella AR (2019). Rab5C enhances resistance to ionizing radiation in rectal cancer. J. Mol. Med (Berl.)..

[128] Pucci S (2017). Ku70, Ku80, and sClusterin: a cluster of predicting factors for response to neoadjuvant chemoradiation therapy in patients with locally advanced rectal cancer. Int J. Radiat. Oncol. Biol. Phys..

[129] O’Connor MJ (2015). Targeting the DNA damage response in cancer. Mol. Cell..

[130] Pott, P. [The first description of an occupational cancer in 1777 (scrotal cancer, cancer of chimney sweeps)]. *Bull. Soc. Liban. Hist. Med*. **11**, 98–101, (1993). 11619233

[131] Kaufmann BP, Gay H (1946). Frequency of recessive lethals induced in Drosophila by near infra-red rays and X-rays. Anat. Rec..

[132] Herskowitz IH (1946). The relationship of x-ray induced recessive lethals to chromosomal breakage. Am. Nat..

[133] Boveri T (2008). Concerning the origin of malignant tumours by Theodor Boveri. Translated and annotated by Henry Harris. J. Cell Sci..

[134] Burdette WJ (1955). The significance of mutation in relation to the origin of tumors: a review. Cancer Res..

[135] Dean CJ, Feldschreiber P, Lett JT (1966). Repair of x-ray damage to the deoxyribonucleic acid in Micrococcus radiodurans. Nature.

[136] Brunk CF, Hanawalt PC (1967). Repair of damaged DNA in a eucaryotic cell: Tetrahymena pyriformis. Science.

[137] Chen Z, Xu XS, Yang J, Wang G (2003). Defining the function of XPC protein in psoralen and cisplatin-mediated DNA repair and mutagenesis. Carcinogenesis.

[138] Cleaver JE (1969). Xeroderma pigmentosum: a human disease in which an initial stage of DNA repair is defective. Proc. Natl Acad. Sci. Usa..

[139] Fadeel B, Orrenius S (2005). Apoptosis: a basic biological phenomenon with wide-ranging implications in human disease. J. Intern Med.

[140] Kerr JF, Wyllie AH, Currie AR (1972). Apoptosis: a basic biological phenomenon with wide-ranging implications in tissue kinetics. Br. J. Cancer.

[141] Bishop JM (1981). Enemies within: the genesis of retrovirus oncogenes. Cell.

[142] Murphree AL, Benedict WF (1984). Retinoblastoma: clues to human oncogenesis. Science.

[143] Chen PL, Chen YM, Bookstein R, Lee WH (1990). Genetic mechanisms of tumor suppression by the human p53 gene. Science.

[144] Kinzler KW, Vogelstein B (1997). Cancer-susceptibility genes. Gatekeepers caretakers. Nat..

[145] Sharan SK (1997). Embryonic lethality and radiation hypersensitivity mediated by Rad51 in mice lacking Brca2. Nature.

[146] Milner J (1997). Transcriptional activation functions in BRCA2. Nature.

[147] Karlsson A (2003). Defective double-strand DNA break repair and chromosomal translocations by MYC overexpression. Proc. Natl Acad. Sci. Usa..

[148] Vafa O (2002). c-Myc can induce DNA damage, increase reactive oxygen species, and mitigate p53 function: a mechanism for oncogene-induced genetic instability. Mol. Cell..

[149] Bartkova J (2005). DNA damage response as a candidate anti-cancer barrier in early human tumorigenesis. Nature.

[150] Pearl LH (2015). Therapeutic opportunities within the DNA damage response. Nat. Rev. Cancer.

[151] Lord CJ, Ashworth A (2012). The DNA damage response and cancer therapy. Nature.

[152] Lee TH, Choi JY, Park JM, Kang TH (2020). Posttranscriptional control of the replication stress response via TTP-mediated Claspin mRNA stabilization. Oncogene.

[153] Glineburg MR, Johns E, Johnson FB (2019). Deletion of ULS1 confers damage tolerance in sgs1 mutants through a Top3-dependent D-loop mediated fork restart pathway. DNA Repair (Amst.).

[154] Joshi RR, Ali SI, Ashley AK (2019). DNA Ligase IV prevents replication fork stalling and promotes cellular proliferation in triple negative breast cancer. J. Nucleic Acids.

[155] Beecher M (2020). Expanding molecular roles of UV-DDB: Shining light on genome stability and cancer. DNA Repair (Amst.)..

[156] Pospiech K, Pluciennik E, Bednarek AK (2018). WWOX tumor suppressor gene in breast cancer, a historical perspective and future directions. Front Oncol..

[157] Jeggo PA, Pearl LH, Carr AM (2016). DNA repair, genome stability and cancer: a historical perspective. Nat. Rev. Cancer.

[158] Bilusic, M. et al. Molecular profiling of exceptional responders to cancer therapy. *Oncologist*, **26**, 186–195 (2020). 10.1002/onco.13600 PMC7930427 33210795

[159] Gotting, I., Jendrossek, V. & Matschke, J. A new twist in protein kinase B/Akt signaling: role of altered cancer cell metabolism in Akt-mediated therapy resistance. *Int J Mol Sci*. **21**, 8563 (2020). 10.3390/ijms21228563 PMC7697684 33202866

[160] Sheth, V. S. & Gauthier, J. Taming the beast: CRS and ICANS after CAR T-cell therapy for ALL. *Bone Marrow Transpl.* **56**, 552–566 (2020). 10.1038/s41409-020-01134-4 PMC8592274 33230186

[161] Luo, G. F., Chen, W. H., Zeng, X. & Zhang, X. Z. Cell primitive-based biomimetic functional materials for enhanced cancer therapy. *Chem. Soc. Rev*. (2020). 10.1039/d0cs00152j 33226037

[162] Pal, D. et al. TGF-beta reduces DNA ds-break repair mechanisms to heighten genetic diversity and adaptability of CD44+/CD24- cancer cells. *Elife*. **6**, (2017). 10.7554/eLife.21615 PMC5345931 28092266

[163] Rossi F (2020). Differences and similarities between cancer and somatic stem cells: therapeutic implications. Stem Cell Res Ther..

[164] Beheshtirouy, S., Mirzaei, F., Eyvazi, S. & Tarhriz, V. Recent advances on therapeutic peptides for breast cancer treatment. *Curr. Protein Pept. Sci*. (2020). 10.2174/1389203721999201117123616 33208071

[165] Wang S, Shi Y, Han X (2020). [Advances in drug resistance mechanisms and prognostic markers of targeted therapy in ALK-positive non-small cell lung cancer]. Zhongguo Fei Ai Za Zhi.

[166] Kumar, V., Yadavilli, S. & Kannan, R. A review on RNAi therapy for NSCLC: Opportunities and challenges. Wiley Interdiscip. Rev. Nanomed. Nanobiotechnol. **13**, e1677, (2020). 10.1002/wnan.1677 33174364

[167] Jiang H, Chen H, Chen N (2020). Construction and validation of a seven-gene signature for predicting overall survival in patients with kidney renal clear cell carcinoma via an integrated bioinformatics analysis. Anim. Cells Syst. (Seoul.).

[168] Caravagna G, Sanguinetti G, Graham TA, Sottoriva A (2020). The MOBSTER R package for tumour subclonal deconvolution from bulk DNA whole-genome sequencing data. BMC Bioinform..

[169] Nie YH (2019). Analysis of mRNA expression patterns in peripheral blood cells of 3 patients with cancer after the first fraction of 2 Gy irradiation: an integrated case report and systematic review. Dose Response.

[170] Liu XD (2019). Integrated analysis of lncRNA-mRNA co-expression networks in the alpha-particle induced carcinogenesis of human branchial epithelial cells. Int J. Radiat. Biol..

[171] Nastasi, C., Mannarino, L. & D’Incalci, M. DNA damage response and immune defense. *Int. J. Mol. Sci*. **21**, 7504 (2020). 10.3390/ijms21207504 PMC7588887 33053746

[172] Roger, E. et al. Maintenance therapy for ATM-deficient pancreatic cancer by multiple DNA damage response interferences after platinum-based chemotherapy. *Cells*. **9**, 2110 (2020). 10.3390/cells9092110 PMC7563330 32948057

[173] Perkhofer, L. et al. DNA damage repair as a target in pancreatic cancer: state-of-the-art and future perspectives. *Gut*, **70**, 606–61 (2020). 10.1136/gutjnl-2019-319984 PMC7873425 32855305

[174] Hanawalt PC (2015). Historical perspective on the DNA damage response. DNA Repair (Amst.)..

[175] Brown EJ, Baltimore D (2000). ATR disruption leads to chromosomal fragmentation and early embryonic lethality. Genes Dev..

[176] Leach FS (1993). Mutations of a mutS homolog in hereditary nonpolyposis colorectal cancer. Cell.

[177] Friedberg EC (2015). A history of the DNA repair and mutagenesis field: I. The discovery of enzymatic photoreactivation. DNA Repair (Amst.)..

[178] Friedberg EC (2011). Nucleotide excision repair of DNA: The very early history. DNA Repair (Amst.)..

[179] Setlow RB, Carrier WL (2003). The disappearance of thymine dimers from DNA: an error-correcting mechanism. 1963. DNA Repair (Amst.).

[180] Kabat S, Visser DW (1964). The incorporation of aminodeoxyuridine into deoxyribonucleic acid of escherichia coli 15t. Biochim Biophys. Acta.

[181] Sachsenmaier C (1994). Damage to DNA by UV light and activation of transcription factors. Biochem Pharmacol..

[182] Cleaver JR, Painter RB (1968). Evidence for repair replication of HeLa cell DNA damaged by ultraviolet light. Biochim Biophys. Acta.

[183] Gellert M (1967). Formation of covalent circles of lambda DNA by E. coli extracts. Proc. Natl Acad. Sci. USA.

[184] Lindahl T (1974). An N-glycosidase from Escherichia coli that releases free uracil from DNA containing deaminated cytosine residues. Proc. Natl Acad. Sci. Usa..

[185] Wagner R, Meselson M (1976). Repair tracts in mismatched DNA heteroduplexes. Proc. Natl Acad. Sci. USA.

[186] Radman M (1975). SOS repair hypothesis: phenomenology of an inducible DNA repair which is accompanied by mutagenesis. Basic Life Sci..

[187] Crowley DJ, Hanawalt PC (1998). Induction of the SOS response increases the efficiency of global nucleotide excision repair of cyclobutane pyrimidine dimers, but not 6-4 photoproducts, in UV-irradiated Escherichia coli. J. Bacteriol..

[188] Hanawalt PC, Spivak G (2008). Transcription-coupled DNA repair: two decades of progress and surprises. Nat. Rev. Mol. Cell Biol..

[189] Ford JM, Hanawalt PC (1995). Li-Fraumeni syndrome fibroblasts homozygous for p53 mutations are deficient in global DNA repair but exhibit normal transcription-coupled repair and enhanced UV resistance. Proc. Natl Acad. Sci. USA.

[190] Deger N (2019). Drosophila, which lacks canonical transcription-coupled repair proteins, performs transcription-coupled repair. J. Biol. Chem..

[191] Kobaisi F (2019). Signaling pathways, chemical and biological modulators of nucleotide excision repair: the faithful shield against UV genotoxicity. Oxid. Med Cell Longev..

[192] Baddock HT (2020). TheSNM1A DNA repair nuclease. DNA Repair (Amst.)..

[193] Sale JE, Translesion DNA (2013). synthesis and mutagenesis in eukaryotes. Cold Spring Harb. Perspect. Biol..

[194] Cortez D (2015). Preventing replication fork collapse to maintain genome integrity. DNA Repair (Amst.)..

[195] Bailey R, Priego Moreno S, Gambus A (2015). Termination of DNA replication forks: "Breaking up is hard to do". Nucleus.

[196] Friedberg EC (2008). A brief history of the DNA repair field. Cell Res..

[197] Lai Y, Beaver JM, Laverde E, Liu Y (2020). Trinucleotide repeat instability via DNA base excision repair. DNA Repair (Amst.).

[198] Sassa A, Odagiri M (2020). Understanding the sequence and structural context effects in oxidative DNA damage repair. DNA Repair (Amst.).

[199] Szewczuk M, Boguszewska K, Kazmierczak-Baranska J, Karwowski BT (2020). The role of AMPK in metabolism and its influence on DNA damage repair. Mol. Biol. Rep..

[200] Kajitani, G. S. et al. Transcription blockage by DNA damage in nucleotide excision repair-related neurological dysfunctions. *Semin. Cell Dev. Biol.*10.1016/j.semcdb.2020.10.009 (2020). 10.1016/j.semcdb.2020.10.009 33229217

[201] Sena, L. A. et al. Tumor frameshift mutation proportion predicts response to immunotherapy in mismatch repair-deficient prostate cancer. *Oncologist.* **26**, e270–e278 (2020). 10.1002/onco.13601 PMC7873327 33215787

[202] Latham, A. et al. Characterization and clinical outcomes of DNA mismatch repair deficient (MMR-D) small bowel adenocarcinoma. *Clin. Cancer Res*, **27**, 1429–1437 (2020). 10.1158/1078-0432.CCR-20-2892 PMC7925361 33199489

[203] Gachechiladze M (2020). Predictive and prognostic value of DNA damage response associated kinases in solid tumors. Front Oncol..

[204] Yoshioka, K. I. & Matsuno, Y. Genomic destabilization and its associated mutagenesis increase with senescence-associated phenotype expression. *Cancer Sci*. **112**, 515–522 (2020). 10.1111/cas.14746 PMC7893996 33222327

[205] Rzeszutek, I. & Betlej, G. The role of small noncoding RNA in DNA double-strand break repair. *Int. J. Mol. Sci*. **21**, 8039 (2020). 10.3390/ijms21218039 PMC7663326 33126669

[206] Couve, S. et al. Direct DNA lesion reversal and excision repair in escherichia coli. *EcoSal Plus*. **5**, **26442931** (2013). 10.1128/ecosalplus.7.2.4 26442931

[207] Dalhus B, Laerdahl JK, Backe PH, Bjoras M (2009). DNA base repair-recognition and initiation of catalysis. FEMS Microbiol. Rev..

[208] Dinant C, Houtsmuller AB, Vermeulen W (2008). Chromatin structure and DNA damage repair. Epigenetics Chromatin.

[209] Park HW, Kim ST, Sancar A, Deisenhofer J (1995). Crystal structure of DNA photolyase from Escherichia coli. Science.

[210] Huang Y (2006). Crystal structure of cryptochrome 3 from Arabidopsis thaliana and its implications for photolyase activity. Proc. Natl Acad. Sci. USA..

[211] Hearst JE (1995). The structure of photolyase: using photon energy for DNA repair. Science.

[212] Eker AP, Quayle C, Chaves I, van der Horst GT (2009). DNA repair in mammalian cells: Direct DNA damage reversal: elegant solutions for nasty problems. Cell Mol. Life Sci..

[213] Ragg S (2000). Direct reversal of DNA damage by mutant methyltransferase protein protects mice against dose-intensified chemotherapy and leads to in vivo selection of hematopoietic stem cells. Cancer Res..

[214] Yi C, He C (2013). DNA repair by reversal of DNA damage. Cold Spring Harb. Perspect. Biol..

[215] Coyne GO (2020). Phase I trial of TRC102 (methoxyamine HCl) in combination with temozolomide in patients with relapsed solid tumors and lymphomas. Oncotarget.

[216] Sinitsky MY (2020). Mitomycin C induced genotoxic stress in endothelial cells is associated with differential expression of proinflammatory cytokines. Mutat. Res..

[217] Klawitter J (2019). Cyclophilin D knockout protects the mouse kidney against cyclosporin A-induced oxidative stress. Am. J. Physiol. Ren. Physiol..

[218] Flitton M (2019). Interaction of nutrition and genetics via DNMT3L-mediated DNA methylation determines cognitive decline. Neurobiol. Aging.

[219] Yang Z (2019). C8-substituted imidazotetrazine analogs overcome temozolomide resistance by inducing DNA adducts and DNA damage. Front Oncol..

[220] Chu CW (2018). GSK3betamediated Ser156 phosphorylation modulates a BH3like domain in BCL2L12 during TMZinduced apoptosis and autophagy in glioma cells. Int J. Mol. Med..

[221] Chen F (2016). Adaptive Response Enzyme AlkB Preferentially Repairs 1-Methylguanine and 3-Methylthymine Adducts in Double-Stranded DNA. Chem. Res Toxicol..

[222] Schoonhoven, N. M. et al. Altering residue 134 confers an increased substrate range of alkylated nucleosides to the E. coli OGT protein. *Molecules*. **22**, 1948 (2017). 10.3390/molecules22111948 PMC6150290 29137116

[223] Denisov AY (2017). Structural basis of interstrand cross-link repair by O(6)-alkylguanine DNA alkyltransferase. Org. Biomol. Chem..

[224] Taira K (2013). Distinct pathways for repairing mutagenic lesions induced by methylating and ethylating agents. Mutagenesis.

[225] Pegg AE (2011). Multifaceted roles of alkyltransferase and related proteins in DNA repair, DNA damage, resistance to chemotherapy, and research tools. Chem. Res Toxicol..

[226] Wibley JE, Pegg AE, Moody PC (2000). Crystal structure of the human O(6)-alkylguanine-DNA alkyltransferase. Nucleic Acids Res.

[227] Vechtomova YL, Telegina TA, Kritsky MS (2020). Evolution of proteins of the DNA photolyase/cryptochrome family. Biochem. (Mosc.).

[228] Sancar A (2003). Structure and function of DNA photolyase and cryptochrome blue-light photoreceptors. Chem. Rev..

[229] Zhong D (2015). Electron transfer mechanisms of DNA repair by photolyase. Annu. Rev. Phys. Chem..

[230] Essen LO, Klar T (2006). Light-driven DNA repair by photolyases. Cell Mol. Life Sci..

[231] Schleicher E (2007). Electron nuclear double resonance differentiates complementary roles for active site histidines in (6-4) photolyase. J. Biol. Chem..

[232] Yamamoto J (2017). Loss of fourth electron-transferring tryptophan in animal (6-4) Photolyase Impairs DNA Repair Activity in Bacterial Cells. Biochemistry.

[233] Zhang M, Wang L, Zhong D (2017). Photolyase: dynamics and electron-transfer mechanisms of DNA repair. Arch. Biochem Biophys..

[234] Kim ST (1994). Characterization of (6-4) photoproduct DNA photolyase. J. Biol. Chem..

[235] Guo X (2015). Dynamics and mechanism of UV-damaged DNA repair in indole-thymine dimer adduct: molecular origin of low repair quantum efficiency. J. Phys. Chem. B..

[236] Liu Z (2012). Electron tunneling pathways and role of adenine in repair of cyclobutane pyrimidine dimer by DNA photolyase. J. Am. Chem. Soc..

[237] Benjdia A (2012). DNA photolyases and SP lyase: structure and mechanism of light-dependent and independent DNA lyases. Curr. Opin. Struct. Biol..

[238] Benjdia A (2012). Structural insights into recognition and repair of UV-DNA damage by Spore Photoproduct Lyase, a radical SAM enzyme. Nucleic Acids Res..

[239] Kiontke S (2011). Crystal structures of an archaeal class II DNA photolyase and its complex with UV-damaged duplex DNA. EMBO J..

[240] Falnes PO, Johansen RF, Seeberg E (2002). AlkB-mediated oxidative demethylation reverses DNA damage in Escherichia coli. Nature.

[241] Trewick SC (2002). Oxidative demethylation by Escherichia coli AlkB directly reverts DNA base damage. Nature.

[242] Zhan, G. et al. Radioprotective effects on late third-instar bactrocera dorsalis (Diptera: Tephritidae) larvae in low-oxygen atmospheres. *Insects*. **11**, 526 (2020). 10.3390/insects11080526 PMC7469153 32806714

[243] Wityk, P., Piatek, R., Nowak, R. & Kostrzewa-Nowak, D. Generation and characterization of a DNA-GCN4 oligonucleotide-peptide conjugate: the impact DNA/protein interactions on the sensitization of DNA. *Molecules*. **25**, 3630 (2020). 10.3390/molecules25163630 PMC7466028 32784992

[244] Forster JC, Douglass MJJ, Phillips WM, Bezak E (2019). Stochastic multicellular modeling of x-ray irradiation, DNA damage induction, DNA free-end misrejoining and cell death. Sci. Rep..

[245] Piekna-Przybylska D (2019). Reporter assays for BER pathway. Methods Mol. Biol..

[246] Wang, K., Maayah, M., Sweasy, J. B. & Alnajjar, K. S. The role of cysteines in the structure and function of OGG1. *J. Biol. Chem*. **296,** 100093 (2020). 10.1074/jbc.RA120.016126 PMC7948458 33203705

[247] Kimura Y, Kajimoto S, Yamamoto Y, Tanaka N (2020). Enzymatic characteristics of Nudix hydrolase 2 (Nud2), an 8-oxo-dGTP hydrolase from Myxococcus xanthus. J. Gen. Appl Microbiol..

[248] Whitaker AM, Freudenthal BD (2018). APE1: A skilled nucleic acid surgeon. DNA Repair (Amst.).

[249] Hendershot JM, O’Brien PJ (2017). Search for DNA damage by human alkyladenine DNA glycosylase involves early intercalation by an aromatic residue. J. Biol. Chem..

[250] Owiti N (2017). Def1 and Dst1 play distinct roles in repair of AP lesions in highly transcribed genomic regions. DNA Repair (Amst.)..

[251] Parsons JL, Dianov GL (2013). Co-ordination of base excision repair and genome stability. DNA Repair (Amst.).

[252] Dianov GL, Hubscher U (2013). Mammalian base excision repair: the forgotten archangel. Nucleic Acids Res..

[253] Liu C (2020). Individualized genetic network analysis reveals new therapeutic vulnerabilities in 6,700 cancer genomes. PLoS Comput Biol..

[254] Cai Y, Geacintov NE, Broyde S (2020). Variable impact of conformationally distinct DNA lesions on nucleosome structure and dynamics: Implications for nucleotide excision repair. DNA Repair (Amst.).

[255] Yudkina AV, Dvornikova AP, Zharkov DO (2018). Variable termination sites of DNA polymerases encountering a DNA-protein cross-link. PLoS One.

[256] Spivak G (2015). Nucleotide excision repair in humans. DNA Repair (Amst.)..

[257] Brevik A (2011). Both base excision repair and nucleotide excision repair in humans are influenced by nutritional factors. Cell Biochem Funct..

[258] Ye N, Bianchi MS, Bianchi NO, Holmquist GP (1999). Adaptive enhancement and kinetics of nucleotide excision repair in humans. Mutat. Res..

[259] Guillotin D, Martin SA (2014). Exploiting DNA mismatch repair deficiency as a therapeutic strategy. Exp. Cell Res..

[260] Huang Y, Li GM (2020). DNA mismatch repair in the chromatin context: Mechanisms and therapeutic potential. DNA Repair (Amst.).

[261] Chakraborty U, Dinh TA, Alani E (2018). Genomic instability promoted by overexpression of mismatch repair factors in yeast: a model for understanding cancer progression. Genetics.

[262] Chakraborty, U. & Alani, E. Understanding how mismatch repair proteins participate in the repair/anti-recombination decision. *FEMS Yeast Res*. **16**, fow071 (2016). 10.1093/femsyr/fow071 PMC5976031 27573382

[263] He D, Li T, Sheng M, Yang B (2020). Exonuclease 1 (Exo1) participates in mammalian non-homologous end joining and contributes to drug resistance in ovarian cancer. Med. Sci. Monit..

[264] Bowen N, Kolodner RD (2017). Reconstitution of Saccharomyces cerevisiae DNA polymerase epsilon-dependent mismatch repair with purified proteins. Proc. Natl Acad. Sci. USA.

[265] Guan J (2021). MLH1 deficiency-triggered DNA hyperexcision by exonuclease 1 activates the cGAS-STING pathway. Cancer Cell..

[266] Motegi A, Masutani M, Yoshioka KI, Bessho T (2019). Aberrations in DNA repair pathways in cancer and therapeutic significances. Semin Cancer Biol..

[267] Boyle KM, Barton JK (2016). Targeting DNA mismatches with rhodium metalloinsertors. Inorg. Chim. Acta.

[268] Dieckman, L. Something’s gotta give: How PCNA alters its structure in response to mutations and the implications on cellular processes. *Prog. Biophys. Mol. Biol.* **163**, 46–59 (2020). 10.1016/j.pbiomolbio.2020.10.008 33161058

[269] Wang H (2020). Mechanisms used by DNA MMR system to cope with Cadmium-induced DNA damage in plants. Chemosphere.

[270] Bradford KC (2020). Dynamic human MutSalpha-MutLalpha complexes compact mismatched DNA. Proc. Natl Acad. Sci. Usa..

[271] Sharma R, Lewis S, Wlodarski MW (2020). DNA repair syndromes and cancer: insights into genetics and phenotype patterns. Front Pediatr..

[272] Nicolas E, Golemis EA, Arora S (2016). POLD1: central mediator of DNA replication and repair, and implication in cancer and other pathologies. Gene.

[273] Li Z, Pearlman AH, Hsieh P (2016). DNA mismatch repair and the DNA damage response. DNA Repair (Amst.).

[274] Xiong J, Zhang J, Li H (2020). Identification of G2 and S phase-expressed-1 as a potential biomarker in patients with prostate cancer. Cancer Manag Res.

[275] Liu A, Yoshioka K, Salerno V, Hsieh P (2008). The mismatch repair-mediated cell cycle checkpoint response to fluorodeoxyuridine. J. Cell Biochem.

[276] Allmann S (2020). Benzo[a]pyrene represses DNA repair through altered E2F1/E2F4 function marking an early event in DNA damage-induced cellular senescence. Nucleic Acids Res..

[277] Suwala, A. K. et al. Primary mismatch repair deficient IDH-mutant astrocytoma (PMMRDIA) is a distinct type with a poor prognosis. *Acta Neuropathol*. **141**, 85–100 (2020). 10.1007/s00401-020-02243-6 PMC7785563 33216206

[278] Brown JS, O’Carrigan B, Jackson SP, Yap TA (2017). Targeting DNA repair in cancer: beyond PARP inhibitors. Cancer Discov..

[279] Mei C (2020). The role of single strand break repair pathways in cellular responses to camptothecin induced DNA damage. Biomed. Pharmacother..

[280] Gaziev AI (1999). [DNA damage in cells exposed to ionizing radiation]. Radiats Biol. Radioecol..

[281] Richardson C, Moynahan ME, Jasin M (1999). Homologous recombination between heterologs during repair of a double-strand break. Suppression of translocations in normal cells. Ann. N. Y Acad. Sci..

[282] Yao Y (2020). ATM promotes RAD51-mediated meiotic DSB repair by inter-sister-chromatid recombination in arabidopsis. Front Plant Sci..

[283] Kumar A, Purohit S, Sharma NK (2016). Aberrant DNA double-strand break repair threads in breast carcinoma: orchestrating genomic insult survival. J. Cancer Prev..

[284] Trenner A, Sartori AA (2019). Harnessing DNA double-strand break repair for cancer treatment. Front Oncol..

[285] Gomez-Mejiba SE, Ramirez DC (2019). Trapping of DNA radicals with the nitrone spin trap 5,5-dimethyl-1-pyrroline N-oxide and genotoxic damage: Recent advances using the immuno-spin trapping technology. Mutat. Res..

[286] Dasika GK (1999). DNA damage-induced cell cycle checkpoints and DNA strand break repair in development and tumorigenesis. Oncogene.

[287] Lai TH (2016). HDAC inhibition induces MicroRNA-182, which targets RAD51 and impairs HR repair to sensitize cells to sapacitabine in acute myelogenous leukemia. Clin. Cancer Res..

[288] Sinha, A. et al. RAD51-mediated DNA homologous recombination is independent of PTEN mutational status. *Cancers (Basel)*. **12**, 3178 (2020). 10.3390/cancers12113178 PMC7693555 33138032

[289] Allera-Moreau C (2012). DNA replication stress response involving PLK1, CDC6, POLQ, RAD51 and CLASPIN upregulation prognoses the outcome of early/mid-stage non-small cell lung cancer patients. Oncogenesis.

[290] Atwell S (2012). Probing Rad51-DNA interactions by changing DNA twist. Nucleic Acids Res..

[291] Dhingra N, Wei L, Zhao X (2019). Replication protein A (RPA) sumoylation positively influences the DNA damage checkpoint response in yeast. J. Biol. Chem..

[292] Gavande NS (2020). Structure-guided optimization of replication protein A (RPA)-DNA Interaction Inhibitors. ACS Med Chem. Lett..

[293] Lyu K, Kumagai A, Dunphy WG (2019). RPA-coated single-stranded DNA promotes the ETAA1-dependent activation of ATR. Cell Cycle.

[294] Prados-Carvajal, R., Rodriguez-Real, G., Gutierrez-Pozo, G. & Huertas, P. CtIP-mediated alternative mRNA splicing finetunes the DNA damage response. *RNA*, (2020). 10.1261/rna.078519.120 PMC7901839 33298529

[295] Mozaffari, N. L., Pagliarulo, F. & Sartori, A. A. Human CtIP: A ’double agent’ in DNA repair and tumorigenesis. *Semin Cell Dev. Biol.* **113**, 47–56 (2020). 10.1016/j.semcdb.2020.09.001 32950401

[296] Batenburg NL (2019). CSB interacts with BRCA1 in late S/G2 to promote MRN- and CtIP-mediated DNA end resection. Nucleic Acids Res..

[297] Soria-Bretones I (2017). DNA end resection requires constitutive sumoylation of CtIP by CBX4. Nat. Commun..

[298] Caston, R. A. et al. The multifunctional APE1 DNA repair-redox signaling protein as a drug target in human disease. *Drug Discov. Today* **26**, 218–228 (2020). 10.1016/j.drudis.2020.10.015 PMC7855940 33148489

[299] Koike M, Koike A (2005). The Ku70-binding site of Ku80 is required for the stabilization of Ku70 in the cytoplasm, for the nuclear translocation of Ku80, and for Ku80-dependent DNA repair. Exp. Cell Res..

[300] Inagawa, T. et al. C-terminal extensions of Ku70 and Ku80 differentially influence DNA end binding properties. *Int. J. Mol. Sci*. **21**, 6725 (2020). 10.3390/ijms21186725 PMC7555691 32937838

[301] Jin S, Weaver DT (1997). Double-strand break repair by Ku70 requires heterodimerization with Ku80 and DNA binding functions. EMBO J..

[302] Shibata A, Jeggo PA (2020). Roles for the DNA-PK complex and 53BP1 in protecting ends from resection during DNA double-strand break repair. J. Radiat. Res.

[303] Hammel, M. et al. Visualizing functional dynamicity in the DNA-dependent protein kinase holoenzyme DNA-PK complex by integrating SAXS with cryo-EM. *Prog. Biophys. Mol. Biol*. **20**, S0079–6107 (2020). 10.1016/j.pbiomolbio.2020.09.003 PMC8896507 32966823

[304] Medunjanin S (2020). DNA-PK: gatekeeper for IKKgamma/NEMO nucleocytoplasmic shuttling in genotoxic stress-induced NF-kappaB activation. Cell Mol. Life Sci..

[305] Su Y (2015). Association of LIG4 and XRCC4 gene polymorphisms with the risk of human glioma in a Chinese population. Int J. Clin. Exp. Pathol..

[306] Gomes BC (2010). The role of common variants of non-homologous end-joining repair genes XRCC4, LIG4 and Ku80 in thyroid cancer risk. Oncol. Rep..

[307] Liu Y (2008). Polymorphisms of LIG4 and XRCC4 involved in the NHEJ pathway interact to modify risk of glioma. Hum. Mutat..

[308] Piccinno R, Minneker V, Roukos V (2019). 53BP1-DNA repair enters a new liquid phase. EMBO J..

[309] Hwang JW (2020). PRMT5 promotes DNA repair through methylation of 53BP1 and is regulated by Src-mediated phosphorylation. Commun. Biol..

[310] Spies J (2019). 53BP1 nuclear bodies enforce replication timing at under-replicated DNA to limit heritable DNA damage. Nat. Cell Biol..

[311] Shibata A, Jeggo PA (2020). Roles for 53BP1 in the repair of radiation-induced DNA double strand breaks. DNA Repair (Amst.).

[312] Zhao L (2020). The determinant of DNA repair pathway choices in ionising radiation-induced DNA double-strand breaks. Biomed Res Int.

[313] Scully R, Panday A, Elango R, Willis NA (2019). DNA double-strand break repair-pathway choice in somatic mammalian cells. Nat. Rev. Mol. Cell Biol..

[314] Truong LN (2013). Microhomology-mediated end joining and homologous recombination share the initial end resection step to repair DNA double-strand breaks in mammalian cells. Proc. Natl Acad. Sci. USA..

[315] Ahrabi S (2016). A role for human homologous recombination factors in suppressing microhomology-mediated end joining. Nucleic Acids Res..

[316] Sallmyr A, Tomkinson AE (2018). Repair of DNA double-strand breaks by mammalian alternative end-joining pathways. J. Biol. Chem..

[317] Huang, Y. et al. Poly(ADP-ribose) polymerase-1 promotes recruitment of meiotic recombination-11 to chromatin and DNA double-strand break repair in Ku70-deficient breast cancer cells. *FASEB J*. **6**, fj201800092R, (2018). 10.1096/fj.201800092R 29874127

[318] Soni A (2014). Requirement for Parp-1 and DNA ligases 1 or 3 but not of Xrcc1 in chromosomal translocation formation by backup end joining. Nucleic Acids Res..

[319] Ghezraoui H (2014). Chromosomal translocations in human cells are generated by canonical nonhomologous end-joining. Mol. Cell..

[320] Mateos-Gomez PA (2015). Mammalian polymerase theta promotes alternative NHEJ and suppresses recombination. Nature.

[321] Iliakis, G., Mladenov, E. & Mladenova, V. Necessities in the processing of DNA double strand breaks and their effects on genomic instability and cancer. *Cancers (Basel)*. **11**, 1671 (2019). 10.3390/cancers11111671 PMC6896103 31661831

[322] Haince JF (2008). PARP1-dependent kinetics of recruitment of MRE11 and NBS1 proteins to multiple DNA damage sites. J. Biol. Chem..

[323] Wang M (2006). PARP-1 and Ku compete for repair of DNA double strand breaks by distinct NHEJ pathways. Nucleic Acids Res..

[324] Wang H (2005). DNA ligase III as a candidate component of backup pathways of nonhomologous end joining. Cancer Res..

[325] Malaby AW, Martin SK, Wood RD, Doublie S (2017). Expression and structural analyses of human DNA polymerase theta (POLQ). Methods Enzymol..

[326] Chan SH, Yu AM, McVey M (2010). Dual roles for DNA polymerase theta in alternative end-joining repair of double-strand breaks in Drosophila. PLoS Genet.

[327] Decottignies A (2013). Alternative end-joining mechanisms: a historical perspective. Front Genet..

[328] Zhao F, Kim W, Kloeber JA, Lou Z (2020). DNA end resection and its role in DNA replication and DSB repair choice in mammalian cells. Exp. Mol. Med.

[329] Averbeck NB (2014). DNA end resection is needed for the repair of complex lesions in G1-phase human cells. Cell Cycle.

[330] Peng H, Zhang S, Chen X (2021). Monitoring 5'-end resection at site-specific double-strand breaks by southern blot analysis. Methods Mol. Biol..

[331] Symington LS, Gautier J (2011). Double-strand break end resection and repair pathway choice. Annu. Rev. Genet.

[332] Tomimatsu N (2014). Phosphorylation of EXO1 by CDKs 1 and 2 regulates DNA end resection and repair pathway choice. Nat. Commun..

[333] Cremona CA (2012). Extensive DNA damage-induced sumoylation contributes to replication and repair and acts in addition to the mec1 checkpoint. Mol. Cell..

[334] Robert T (2011). HDACs link the DNA damage response, processing of double-strand breaks and autophagy. Nature.

[335] Jimeno S (2015). Neddylation inhibits CtIP-mediated resection and regulates DNA double strand break repair pathway choice. Nucleic Acids Res..

[336] Bunting SF (2010). 53BP1 inhibits homologous recombination in Brca1-deficient cells by blocking resection of DNA breaks. Cell.

[337] Callen E (2020). 53BP1 enforces distinct pre- and post-resection blocks on homologous recombination. Mol. Cell..

[338] Wang H (2013). The interaction of CtIP and Nbs1 connects CDK and ATM to regulate HR-mediated double-strand break repair. PLoS Genet..

[339] Jachimowicz RD, Reinhardt HC (2019). UBQLN4 promotes non-homologous end joining by repressing DNA end-resection. Mol. Cell Oncol..

[340] Ceccaldi R (2015). Homologous-recombination-deficient tumours are dependent on Poltheta-mediated repair. Nature.

[341] Howard SM, Yanez DA, Stark JM (2015). DNA damage response factors from diverse pathways, including DNA crosslink repair, mediate alternative end joining. PLoS Genet.

[342] Unno J (2014). FANCD2 binds CtIP and regulates DNA-end resection during DNA interstrand crosslink repair. Cell Rep..

[343] Iliakis G, Murmann T, Soni A (2015). Alternative end-joining repair pathways are the ultimate backup for abrogated classical non-homologous end-joining and homologous recombination repair: Implications for the formation of chromosome translocations. Mutat. Res Genet Toxicol. Environ. Mutagen.

[344] Janssen A (2016). A single double-strand break system reveals repair dynamics and mechanisms in heterochromatin and euchromatin. Genes Dev..

[345] Liao S, Tammaro M, Yan H (2016). The structure of ends determines the pathway choice and Mre11 nuclease dependency of DNA double-strand break repair. Nucleic Acids Res.

[346] Azenha D, Lopes MC, Martins TC (2019). Claspin: From replication stress and DNA damage responses to cancer therapy. Adv. Protein Chem. Struct. Biol..

[347] Tian H (2015). DNA damage response-a double-edged sword in cancer prevention and cancer therapy. Cancer Lett..

[348] Neizer-Ashun F, Bhattacharya R (2021). Reality CHEK: Understanding the biology and clinical potential of CHK1. Cancer Lett..

[349] Zhang Y, Hunter T (2014). Roles of Chk1 in cell biology and cancer therapy. Int J. Cancer.

[350] Peddibhotla S, Lam MH, Gonzalez-Rimbau M, Rosen JM (2009). The DNA-damage effector checkpoint kinase 1 is essential for chromosome segregation and cytokinesis. Proc. Natl Acad. Sci. USA.

[351] Carrassa L, Colombo I, Damia G, Bertoni F (2020). Targeting the DNA damage response for patients with lymphoma: Preclinical and clinical evidences. Cancer Treat. Rev..

[352] Khanna A (2015). DNA damage in cancer therapeutics: a boon or a curse?. Cancer Res..

[353] Kaemmerer, E., Loessner, D. & Avery, V. M. Addressing the tumour microenvironment in early drug discovery: a strategy to overcome drug resistance and identify novel targets for cancer therapy. *Drug Discov. Today*, **26**, 663–676 (2020). 10.1016/j.drudis.2020.11.030 33278601

[354] Karanika S (2015). DNA damage response and prostate cancer: defects, regulation and therapeutic implications. Oncogene.

[355] Cazaux C (2010). [Genetic instability as a driver for oncogenesis]. Bull. Cancer.

[356] Contreras HR, Lopez-Moncada F, Castellon EA (2020). Cancer stem cell and mesenchymal cell cooperative actions in metastasis progression and hormone resistance in prostate cancer: Potential role of androgen and gonadotropinreleasing hormone receptors (Review). Int J. Oncol..

[357] Zeng X (2020). Breast cancer stem cells, heterogeneity, targeting therapies and therapeutic implications. Pharm. Res..

[358] Anichini, A., Perotti, V. E., Sgambelluri, F. & Mortarini, R. Immune escape mechanisms in non small cell lung cancer. *Cancers (Basel)*. **12**, 3605 (2020). 10.3390/cancers12123605 PMC7761620 33276569

[359] Xia WY (2020). Radiotherapy for non-small cell lung cancer in the immunotherapy era: the opportunity and challenge-a narrative review. Transl. Lung Cancer Res..

[360] Chakravarty D, Huang L, Kahn M, Tewari AK (2020). Immunotherapy for metastatic prostate cancer: current and emerging treatment options. Urol. Clin. North Am..

[361] Keam S (2020). Enhancing the efficacy of immunotherapy using radiotherapy. Clin. Transl. Immunol..

[362] Carlson, R. D., Flickinger, J. C., Jr & Snook, A. E. Talkin’ toxins: from Coley’s to modern cancer immunotherapy. *Toxins (Basel)*. **12**, 241 (2020). 10.3390/toxins12040241 PMC7232517 32283684

[363] Coley WBII (1891). Contribution to the Knowledge of Sarcoma. Ann. Surg..

[364] Coley WB (1910). The treatment of inoperable sarcoma by bacterial toxins (the mixed toxins of the streptococcus erysipelas and the bacillus prodigiosus). Proc. R. Soc. Med..

[365] Johnston BJ, Novales ET (1962). Clinical effect of Coley’s toxin. II. A seven-year study. Cancer Chemother. Rep..

[366] Bonnichon P (2014). [History of cancer and chemotherapy before chemotherapy]. Hist. Sci. Med..

[367] Walunas TL (1994). CTLA-4 can function as a negative regulator of T cell activation. Immunity.

[368] Walunas TL, Bakker CY, Bluestone JA (1996). CTLA-4 ligation blocks CD28-dependent T cell activation. J. Exp. Med.

[369] Kim MT (2015). Enhancing dendritic cell-based immunotherapy with IL-2/monoclonal antibody complexes for control of established tumors. J. Immunol..

[370] Kasagi S, Kawano S, Kumagai S (2011). PD-1 and autoimmunity. Crit. Rev. Immunol..

[371] Kwon ED (1997). Manipulation of T cell costimulatory and inhibitory signals for immunotherapy of prostate cancer. Proc. Natl Acad. Sci. Usa..

[372] Hurwitz AA (1997). Specific blockade of CTLA-4/B7 interactions results in exacerbated clinical and histologic disease in an actively-induced model of experimental allergic encephalomyelitis. J. Neuroimmunol..

[373] Dunn GP (2002). Cancer immunoediting: from immunosurveillance to tumor escape. Nat. Immunol..

[374] Hsieh EM, Rouce RH (2020). Chimeric antigen receptor T cells for mature B-cell lymphoma and Burkitt lymphoma. Hematol. Am. Soc. Hematol. Educ. Program.

[375] Baxevanis, C. N., Fortis, S. P., Ardavanis, A. & Perez, S. A. Exploring essential issues for improving therapeutic cancer vaccine trial design. *Cancers (Basel)*. **12**, 2908 (2020). 10.3390/cancers12102908 PMC7600460 33050520

[376] Hwang, J. K., Hong, J. & Yun, C. O. Oncolytic viruses and immune checkpoint inhibitors: preclinical developments to clinical trials. *Int J Mol Sci*. **21**, 8627 (2020). 10.3390/ijms21228627 PMC7697902 33207653

[377] Hargadon, K. M., Gyorffy, B. & McGee, T. J. Genomic and transcriptional changes in IFNgamma pathway genes are putative biomarkers of response to ipilimumab immunotherapy in melanoma patients. *Expert Rev Clin Immunol.*10.1080/1744666X.2021.1847644 (2020). 10.1080/1744666X.2021.1847644 33151785

[378] Wright K (2020). FDA approves nivolumab plus ipilimumab for previously untreated unresectable malignant pleural mesothelioma. Oncol. (Williston Park)..

[379] Fessas, P. et al. Post-registration experience of nivolumab in advanced hepatocellular carcinoma: an international study. *J Immunother Cancer*. **8**, e001033 (2020). 10.1136/jitc-2020-001033 PMC7462152 32868393

[380] Koch, M. S., Lawler, S. E. & Chiocca, E. A. HSV-1 oncolytic viruses from bench to bedside: an overview of current clinical trials. *Cancers (Basel)*. **12**, 3514(2020). 10.3390/cancers12123514 PMC7760226 33255871

[381] Muller, L. et al. Past, present and future of oncolytic reovirus. *Cancers (Basel)*. **12**, 3219 (2020). 10.3390/cancers12113219 PMC7693452 33142841

[382] Hamid O, Ismail R, Puzanov I (2020). Intratumoral Immunotherapy-Update 2019. Oncologist.

[383] Broman KK, Zager JS (2020). An evaluation of talimogene laherparepvec for the treatment of melanoma. Expert Opin. Biol. Ther..

[384] Mulbauer GD, Matthew HWT (2019). Biomimetic scaffolds in skeletal muscle regeneration. Discoveries (Craiova).

[385] Gopalakrishnan V (2018). Gut microbiome modulates response to anti-PD-1 immunotherapy in melanoma patients. Science.

[386] Routy B (2018). Gut microbiome influences efficacy of PD-1-based immunotherapy against epithelial tumors. Science.

[387] Reislander T, Groelly FJ, Tarsounas M (2020). DNA Damage and Cancer Immunotherapy: A STING in the Tale. Mol. Cell.

[388] Harding SM (2017). Mitotic progression following DNA damage enables pattern recognition within micronuclei. Nature.

[389] Mackenzie KJ (2017). cGAS surveillance of micronuclei links genome instability to innate immunity. Nature.

[390] Unterholzner L, Dunphy G (2019). cGAS-independent STING activation in response to DNA damage. Mol. Cell Oncol..

[391] Dunphy G (2018). Non-canonical Activation of the DNA Sensing Adaptor STING by ATM and IFI16 Mediates NF-kappaB Signaling after Nuclear DNA Damage. Mol. Cell..

[392] Storozynsky, Q. & Hitt, M. M. The impact of radiation-induced DNA damage on cGAS-STING-mediated immune responses to cancer. *Int. J. Mol. Sci*. **21**, 8877 (2020). 10.3390/ijms21228877 PMC7700321 33238631

[393] Almine JF (2017). IFI16 and cGAS cooperate in the activation of STING during DNA sensing in human keratinocytes. Nat. Commun..

[394] Kikuchi T (2019). A subset of patients with MSS/MSI-low-colorectal cancer showed increased CD8(+) TILs together with up-regulated IFN-gamma. Oncol. Lett..

[395] Takeda K (2017). IFN-gamma is required for cytotoxic T cell-dependent cancer genome immunoediting. Nat. Commun..

[396] Coquel F (2020). [SAMHD1 acts at stalled replication forks to prevent interferon induction]. C. R. Biol..

[397] Erdal E (2017). A prosurvival DNA damage-induced cytoplasmic interferon response is mediated by end resection factors and is limited by Trex1. Genes Dev..

[398] Tarsounas M, Sung P (2020). The antitumorigenic roles of BRCA1-BARD1 in DNA repair and replication. Nat. Rev. Mol. Cell Biol..

[399] Fu J, Mao J, Wang C (2020). The microRNA-152/human leukocyte antigen-G axis affects proliferation and immune escape of non-small cell lung cancer cells. J. Int Med Res.

[400] Wei SC, Duffy CR, Allison JP (2018). Fundamental mechanisms of immune checkpoint blockade therapy. Cancer Discov..

[401] Buchbinder EI, Desai A (2016). CTLA-4 and PD-1 pathways: similarities, differences, and implications of their inhibition. Am. J. Clin. Oncol..

[402] Havel JJ, Chowell D, Chan TA (2019). The evolving landscape of biomarkers for checkpoint inhibitor immunotherapy. Nat. Rev. Cancer.

[403] Gasser S, Raulet D (2006). The DNA damage response, immunity and cancer. Semin Cancer Biol..

[404] Gasser S, Raulet DH (2006). The DNA damage response arouses the immune system. Cancer Res..

[405] Nakad R, Schumacher B (2016). DNA damage response and immune defense: links and mechanisms. Front Genet.

[406] Cerboni C (2014). The DNA damage response: a common pathway in the regulation of NKG2D and DNAM-1 ligand expression in normal, infected, and cancer cells. Front Immunol..

[407] Raulet DH, Marcus A, Coscoy L (2017). Dysregulated cellular functions and cell stress pathways provide critical cues for activating and targeting natural killer cells to transformed and infected cells. Immunol. Rev..

[408] Gasser S, Orsulic S, Brown EJ, Raulet DH (2005). The DNA damage pathway regulates innate immune system ligands of the NKG2D receptor. Nature.

[409] Jinushi M (2012). ATM-mediated DNA damage signals mediate immune escape through integrin-alphavbeta3-dependent mechanisms. Cancer Res..

[410] Yang, H. et al. Beyond DNA Repair: DNA-PKcs in tumor metastasis, metabolism and immunity. *Cancers (Basel)*. **12**, 3389 (2020). 10.3390/cancers12113389 PMC7698146 33207636

[411] Li H (2011). Pharmacological activation of p53 triggers anticancer innate immune response through induction of ULBP2. Cell Cycle.

[412] Wang Y, Shi H, Meng H, Xu J (2020). Editorial: targeting the PD-1/PD-L1 cancer immune evasion axis: challenges and emerging strategies. Front Pharm..

[413] Sharma P, Allison JP (2015). The future of immune checkpoint therapy. Science.

[414] Facchini G (2020). Advanced/metastatic bladder cancer: current status and future directions. Eur. Rev. Med Pharm. Sci..

[415] Hu Z (2017). The future of immune checkpoint blockade immunotherapy: towards personalized therapy or towards combination therapy. J. Thorac. Dis..

[416] Innao, V., Allegra, A. G., Musolino, C. & Allegra, A. New frontiers about the role of human microbiota in immunotherapy: the immune checkpoint inhibitors and CAR T-Cell Therapy Era. *Int. J. Mol. Sci.* **21**, 8902 (2020). 10.3390/ijms21238902 PMC7727716 33255336

[417] Ponnusamy L, Mahalingaiah PKS, Singh KP (2020). Epigenetic reprogramming and potential application of epigenetic-modifying drugs in acquired chemotherapeutic resistance. Adv. Clin. Chem..

[418] Tazzite A, Jouhadi H, Benider A, Nadifi S (2020). BRCA mutational status is a promising predictive biomarker for platinum- based chemotherapy in triple-negative breast cancer. Curr. Drug Targets.

[419] Crusz SM, Miller RE (2020). Targeted therapies in gynaecological cancers. Histopathology.

[420] Klinakis A, Karagiannis D, Rampias T (2020). Targeting DNA repair in cancer: current state and novel approaches. Cell Mol. Life Sci..

[421] Rose M (2020). PARP inhibitors: clinical relevance, mechanisms of action and tumor resistance. Front Cell Dev. Biol..

[422] Harrision D, Gravells P, Thompson R, Bryant HE (2020). Poly(ADP-Ribose) Glycohydrolase (PARG) vs. Poly(ADP-Ribose) Polymerase (PARP) - Function in Genome Maintenance and Relevance of Inhibitors for Anti-cancer Therapy. Front Mol. Biosci..

[423] Cerrato A, Morra F, Celetti A (2016). Use of poly ADP-ribose polymerase [PARP] inhibitors in cancer cells bearing DDR defects: the rationale for their inclusion in the clinic. J. Exp. Clin. Cancer Res.

[424] Fisher AE, Hochegger H, Takeda S, Caldecott KW (2007). Poly(ADP-ribose) polymerase 1 accelerates single-strand break repair in concert with poly(ADP-ribose) glycohydrolase. Mol. Cell Biol..

[425] Sugimura K (2008). PARP-1 ensures regulation of replication fork progression by homologous recombination on damaged DNA. J. Cell Biol..

[426] Dockery LE, Gunderson CC, Moore KN (2017). Rucaparib: the past, present, and future of a newly approved PARP inhibitor for ovarian cancer. Onco Targets Ther..

[427] Preiss J, Schlaeger R, Hilz H (1971). Specific inhibition of poly adpribose polymerase by thymidine and nicotinamide in HeLa cells. FEBS Lett..

[428] Lemjabbar-Alaoui H, Peto CJ, Yang YW, Jablons DM (2020). AMXI-5001, a novel dual parp1/2 and microtubule polymerization inhibitor for the treatment of human cancers. Am. J. Cancer Res.

[429] Geenen JJJ, Linn SC, Beijnen JH, Schellens JHM (2018). PARP Inhibitors in the Treatment of Triple-Negative Breast Cancer. Clin. Pharmacokinet..

[430] Bryant HE (2005). Specific killing of BRCA2-deficient tumours with inhibitors of poly(ADP-ribose) polymerase. Nature.

[431] Kaufman B (2015). Olaparib monotherapy in patients with advanced cancer and a germline BRCA1/2 mutation. J. Clin. Oncol..

[432] Pujade-Lauraine E (2017). Olaparib tablets as maintenance therapy in patients with platinum-sensitive, relapsed ovarian cancer and a BRCA1/2 mutation (SOLO2/ENGOT-Ov21): a double-blind, randomised, placebo-controlled, phase 3 trial. Lancet Oncol..

[433] Olaparib for Metastatic Breast Cancer in Patients with a Germline BRCA Mutation. *N. Engl. J. Med*. **377**, 1700, (2017). 10.1056/NEJMx170012 28792849

[434] Jang, A., Sartor, O., Barata, P. C. & Paller, C. J. Therapeutic potential of PARP inhibitors in the treatment of metastatic castration-resistant prostate cancer. *Cancers (Basel)*. **12**, 3467 (2020). 10.3390/cancers12113467 PMC7700539 33233320

[435] Oza AM (2017). Antitumor activity and safety of the PARP inhibitor rucaparib in patients with high-grade ovarian carcinoma and a germline or somatic BRCA1 or BRCA2 mutation: Integrated analysis of data from Study 10 and ARIEL2. Gynecol. Oncol..

[436] Gonzalez-Martin A (2019). Niraparib in patients with newly diagnosed advanced ovarian cancer. N. Engl. J. Med..

[437] Mirza MR (2016). Niraparib maintenance therapy in platinum-sensitive, recurrent ovarian cancer. N. Engl. J. Med..

[438] Ettl J (2018). Quality of life with talazoparib versus physician’s choice of chemotherapy in patients with advanced breast cancer and germline BRCA1/2 mutation: patient-reported outcomes from the EMBRACA phase III trial. Ann. Oncol..

[439] Baxter PA (2020). A phase I/II study of veliparib (ABT-888) with radiation and temozolomide in newly diagnosed diffuse pontine glioma: a Pediatric Brain Tumor Consortium study. Neuro Oncol..

[440] Luo J (2020). Fluzoparib increases radiation sensitivity of non-small cell lung cancer (NSCLC) cells without BRCA1/2 mutation, a novel PARP1 inhibitor undergoing clinical trials. J. Cancer Res Clin. Oncol..

[441] Abbotts R, Madhusudan S (2010). Human AP endonuclease 1 (APE1): from mechanistic insights to druggable target in cancer. Cancer Treat. Rev..

[442] Tell G, Damante G, Caldwell D, Kelley MR (2005). The intracellular localization of APE1/Ref-1: more than a passive phenomenon?. Antioxid. Redox Signal.

[443] Herring CJ (1998). Levels of the DNA repair enzyme human apurinic/apyrimidinic endonuclease (APE1, APEX, Ref-1) are associated with the intrinsic radiosensitivity of cervical cancers. Br. J. Cancer.

[444] Wang D (2009). APE1 overexpression is associated with cisplatin resistance in non-small cell lung cancer and targeted inhibition of APE1 enhances the activity of cisplatin in A549 cells. Lung Cancer.

[445] Felix FA (2021). DNA base excision repair and nucleotide excision repair proteins in malignant salivary gland tumors. Arch. Oral. Biol..

[446] Manoel-Caetano FS (2020). Hydrogen peroxide and Helicobacter pylori extract treatment combined with APE1 knockdown induce DNA damage, G2/M arrest and cell death in gastric cancer cell line. DNA Repair (Amst.)..

[447] Kabzinski J, Walczak A, Mik M, Majsterek I (2019). Sirt3 regulates the level of mitochondrial DNA repair activity through deacetylation of NEIL1, NEIL2, OGG1, MUTYH, APE1 and LIG3 in colorectal cancer. Pol. Przegl Chir..

[448] Laev SS, Salakhutdinov NF, Lavrik OI (2017). Inhibitors of nuclease and redox activity of apurinic/apyrimidinic endonuclease 1/redox effector factor 1 (APE1/Ref-1). Bioorg. Med. Chem..

[449] Gordon MS (2013). A phase 1 study of TRC102, an inhibitor of base excision repair, and pemetrexed in patients with advanced solid tumors. Invest N. Drugs.

[450] Franchi LP, de Freitas Lima JEB, Piva HL, Tedesco AC (2020). The redox function of apurinic/apyrimidinic endonuclease 1 as key modulator in photodynamic therapy. J. Photochem Photobio. B.

[451] Madhusudan S (2005). Isolation of a small molecule inhibitor of DNA base excision repair. Nucleic Acids Res..

[452] Nobler MP, Scher AJ (1978). Lucanthone as a radiosensitizing agent in the treatment of carcinoma of the cervix. Int J. Radiat. Oncol. Biol. Phys..

[453] Carew JS (2011). Lucanthone is a novel inhibitor of autophagy that induces cathepsin D-mediated apoptosis. J. Biol. Chem..

[454] Cook JA, Jordan P, Woodstock L, Pilgrim V (1977). A controlled trial of hycanthone and placebo in schistosomiasis mansoni in St. Lucia. Ann. Trop. Med Parasitol..

[455] Del Rowe JD (1999). Accelerated regression of brain metastases in patients receiving whole brain radiation and the topoisomerase II inhibitor, lucanthone. Int J. Radiat. Oncol. Biol. Phys..

[456] Mendez F, Goldman JD, Bases RE (2002). Abasic sites in DNA of HeLa cells induced by lucanthone. Cancer Invest.

[457] Fishel ML, Kelley MR (2007). The DNA base excision repair protein Ape1/Ref-1 as a therapeutic and chemopreventive target. Mol. Asp. Med.

[458] Ding J (2017). Ref-1/APE1 as a transcriptional regulator and novel therapeutic target in pediatric t-cell leukemia. Mol. Cancer Ther..

[459] Guerreiro PS (2017). The APE1 redox inhibitor E3330 reduces collective cell migration of human breast cancer cells and decreases chemoinvasion and colony formation when combined with docetaxel. Chem. Biol. Drug Des..

[460] London RE (2020). XRCC1 - Strategies for coordinating and assembling a versatile DNA damage response. DNA Repair (Amst.)..

[461] Abbotts R, Wilson DM (2017). Coordination of DNA single strand break repair. Free Radic. Biol. Med.

[462] Iyama T, Wilson DM (2013). DNA repair mechanisms in dividing and non-dividing cells. DNA Repair (Amst.).

[463] Ferri, A., Stagni, V. & Barila, D. Targeting the DNA damage response to overcome cancer drug resistance in glioblastoma. *Int. J. Mol. Sci*. **21**, 4910 (2020). 10.3390/ijms21144910 PMC7402284 32664581

[464] O’Connor E (2018). Mutations in XRCC1 cause cerebellar ataxia and peripheral neuropathy. J. Neurol. Neurosurg. Psychiatry.

[465] Zhang N (2020). Pharmacogenetic association between XRCC1 polymorphisms and response to platinum-based chemotherapy in asian patients with NSCLC: a meta-analysis. Biomed. Res Int..

[466] Mani RS (2019). Domain analysis of PNKP-XRCC1 interactions: Influence of genetic variants of XRCC1. J. Biol. Chem..

[467] Xie X (2020). Radiation-induced lymphopenia during chemoradiation therapy for non-small cell lung cancer is linked with age, lung V5, and XRCC1 rs25487 genotypes in lymphocytes. Radiother. Oncol..

[468] Raturi V (2020). Prospective evaluation of XRCC-1 Arg194Trp polymorphism as bio-predictor for clinical outcome in locally advanced laryngeal cancer undergoing cisplatin-based chemoradiation. Head. Neck..

[469] Nanda SS (2018). Evaluation of XRCC1 gene polymorphism as a biomarker in head and neck cancer patients undergoing chemoradiation therapy. Int J. Radiat. Oncol. Biol. Phys..

[470] Figg WD (2013). Phase II study of satraplatin and prednisone in patients with metastatic castration-resistant prostate cancer: a pharmacogenetic assessment of outcome and toxicity. Clin. Genitourin. Cancer.

[471] Ott K (2011). DNA repair gene and MTHFR gene polymorphisms as prognostic markers in locally advanced adenocarcinoma of the esophagus or stomach treated with cisplatin and 5-fluorouracil-based neoadjuvant chemotherapy. Ann. Surg. Oncol..

[472] Vaezi A, Feldman CH, Niedernhofer LJ (2011). ERCC1 and XRCC1 as biomarkers for lung and head and neck cancer. Pharmgenomics Pers. Med.

[473] Huang MY (2011). Multiple genetic polymorphisms in the prediction of clinical outcome of metastatic colorectal cancer patients treated with first-line FOLFOX-4 chemotherapy. Pharmacogenet Genomics..

[474] Tung CL (2015). Down-regulation of ERK1/2 and AKT-mediated X-ray repair cross-complement group 1 protein (XRCC1) expression by Hsp90 inhibition enhances the gefitinib-induced cytotoxicity in human lung cancer cells. Exp. Cell Res..

[475] Davis AJ, Lee KJ, Chen DJ (2013). The N-terminal region of the DNA-dependent protein kinase catalytic subunit is required for its DNA double-stranded break-mediated activation. J. Biol. Chem..

[476] Neal JA (2014). Unraveling the complexities of DNA-dependent protein kinase autophosphorylation. Mol. Cell Biol..

[477] Dobbs TA, Tainer JA, Lees-Miller SP (2010). A structural model for regulation of NHEJ by DNA-PKcs autophosphorylation. DNA Repair (Amst.).

[478] Sun X (2020). DNA-PK deficiency potentiates cGAS-mediated antiviral innate immunity. Nat. Commun..

[479] Li Y, Goronzy JJ, Weyand CM (2018). DNA damage, metabolism and aging in pro-inflammatory T cells: Rheumatoid arthritis as a model system. Exp. Gerontol..

[480] Yu ZJ (2004). [Expression of DNA-PK in hepato- and cholangio-neoplasms and its significance]. Zhonghua Gan Zang Bing Za Zhi.

[481] An J (2005). Silencing of DNA-PKcs alters the transcriptional profile of certain signal transduction genes related to proliferation and differentiation in HeLa cells. Int J. Mol. Med..

[482] An J (2005). Downregulation of c-myc protein by siRNA-mediated silencing of DNA-PKcs in HeLa cells. Int J. Cancer.

[483] Guo Z (2020). HUWE1-dependent DNA-PKcs neddylation modulates its autophosphorylation in DNA damage response. Cell Death Dis..

[484] Goldberg FW (2020). The Discovery of 7-Methyl-2-[(7-methyl[1,2,4]triazolo[1,5-a]pyridin-6-yl)amino]−9-(tetrahydro-2H-p yran-4-yl)−7,9-dihydro-8H-purin-8-one (AZD7648), a Potent and Selective DNA-Dependent Protein Kinase (DNA-PK) Inhibitor. J Med Chem.

[485] Dylgjeri E (2019). Pleiotropic impact of DNA-PK in cancer and implications for therapeutic strategies. Clin. Cancer Res..

[486] Wu ZX (2020). M3814, a DNA-PK inhibitor, modulates ABCG2-mediated multidrug resistance in lung cancer cells. Front Oncol..

[487] Fok JHL (2019). AZD7648 is a potent and selective DNA-PK inhibitor that enhances radiation, chemotherapy and olaparib activity. Nat. Commun..

[488] Khan AJ (2018). VX-984 is a selective inhibitor of non-homologous end joining, with possible preferential activity in transformed cells. Oncotarget.

[489] Timme CR (2018). The DNA-PK inhibitor VX-984 enhances the radiosensitivity of glioblastoma cells grown in vitro and as orthotopic xenografts. Mol. Cancer Ther..

[490] Berzosertib Is Safe, with Signs of Efficacy against Advanced Solid Tumors. *Cancer Discov*. **10**, 1250, (2020).

[491] McMullen M (2020). DUETTE: a phase II randomized, multicenter study to investigate the efficacy and tolerability of a second maintenance treatment in patients with platinum-sensitive relapsed epithelial ovarian cancer, who have previously received poly(ADP-ribose) polymerase (PARP) inhibitor maintenance treatment. Int J. Gynecol. Cancer.

[492] Bradbury A, Hall S, Curtin N, Drew Y (2020). Targeting ATR as cancer therapy: a new era for synthetic lethality and synergistic combinations?. Pharm. Ther..

[493] Ellenberger T, Tomkinson AE (2008). Eukaryotic DNA ligases: structural and functional insights. Annu. Rev. Biochem.

[494] Britton S (2020). ATM antagonizes NHEJ proteins assembly and DNA-ends synapsis at single-ended DNA double strand breaks. Nucleic Acids Res..

[495] Tomkinson AE, Naila T (2020). Altered DNA ligase activity in human disease. Mutagenesis.

[496] Riyazuddin M (2019). Elucidation of pharmacokinetics of novel DNA ligase I inhibitor, S012-1332 in rats: Integration of in vitro and in vivo findings. J. Pharm. Biomed. Anal..

[497] Howes TRL (2017). Structure-activity relationships among DNA ligase inhibitors: Characterization of a selective uncompetitive DNA ligase I inhibitor. DNA Repair (Amst.)..

[498] Hussain MK (2017). A novel benzocoumarin-stilbene hybrid as a DNA ligase I inhibitor with in vitro and in vivo anti-tumor activity in breast cancer models. Sci. Rep..

[499] Chen X (2008). Rational design of human DNA ligase inhibitors that target cellular DNA replication and repair. Cancer Res..

[500] Srivastava M (2012). An inhibitor of nonhomologous end-joining abrogates double-strand break repair and impedes cancer progression. Cell.

[501] Ray U (2020). Identification and characterization of novel SCR7-based small-molecule inhibitor of DNA end-joining, SCR130 and its relevance in cancer therapeutics. Mol. Carcinog..

[502] Huang R, Xiang J, Zhou P (2019). Vitamin D, gut microbiota, and radiation-related resistance: a love-hate triangle. J. Exp. Clin. Cancer Res.

[503] Drullinsky PR, Hurvitz SA (2020). Mechanistic basis for PI3K inhibitor antitumor activity and adverse reactions in advanced breast cancer. Breast Cancer Res Treat..

[504] Tan AC (2020). Targeting the PI3K/Akt/mTOR pathway in non-small cell lung cancer (NSCLC). Thorac. Cancer.

[505] Dent S (2021). Phase III randomized study of taselisib or placebo with fulvestrant in estrogen receptor-positive, PIK3CA-mutant, HER2-negative, advanced breast cancer: the SANDPIPER trial. Ann. Oncol..

[506] Bertho M (2021). A pharmacokinetic evaluation of alpelisib for the treatment of HR+, HER2-negative, PIK3CA-mutated advanced or metastatic breast cancer. Expert Opin. Drug Metab. Toxicol..

[507] Matasar, M. J. et al. Copanlisib plus rituximab versus placebo plus rituximab in patients with relapsed indolent non-Hodgkin lymphoma (CHRONOS-3): a double-blind, randomised, placebo-controlled, phase 3 trial. *Lancet Oncol.*10.1016/S1470-2045(21)00145-5 (2021). 10.1016/S1470-2045(21)00145-5 33848462

[508] Leenhardt F, Alexandre M, Jacot W (2021). Alpelisib for the treatment of PIK3CA-mutated, hormone receptor-positive, HER2-negative metastatic breast cancer. Expert Opin. Pharmacother..

[509] Garrido-Castro AC (2020). Phase 2 study of buparlisib (BKM120), a pan-class I PI3K inhibitor, in patients with metastatic triple-negative breast cancer. Breast Cancer Res..

[510] Kaklamani VG, Richardson AL, Arteaga CL (2019). Exploring biomarkers of phosphoinositide 3-kinase pathway activation in the treatment of hormone receptor positive, human epidermal growth receptor 2 negative advanced breast cancer. Oncologist.

[511] Dreyling M (2017). Phosphatidylinositol 3-kinase inhibition by copanlisib in relapsed or refractory indolent lymphoma. J. Clin. Oncol..

[512] Buckley AM, Lynam-Lennon N, O’Neill H, O'Sullivan J (2020). Targeting hallmarks of cancer to enhance radiosensitivity in gastrointestinal cancers. Nat. Rev. Gastroenterol. Hepatol..

[513] Verma N, Tiku AB (2017). Significance and nature of bystander responses induced by various agents. Mutat. Res.

[514] Hendijani F, Javanmard SH (2015). Dual protective and cytotoxic benefits of mesenchymal stem cell therapy in combination with chemotherapy/radiotherapy for cancer patients. Crit. Rev. Eukaryot. Gene Expr..

[515] Tao Y (2020). Avelumab-cetuximab-radiotherapy versus standards of care in locally advanced squamous-cell carcinoma of the head and neck: The safety phase of a randomised phase III trial GORTEC 2017-01 (REACH). Eur. J. Cancer.

[516] Kunos CA (2019). Randomized phase II trial of triapine-cisplatin-radiotherapy for locally advanced stage uterine cervix or vaginal cancers. Front Oncol..

[517] Ciszewski WM, Tavecchio M, Dastych J, Curtin NJ (2014). DNA-PK inhibition by NU7441 sensitizes breast cancer cells to ionizing radiation and doxorubicin. Breast Cancer Res. Treat..

[518] Sun X (2012). Identification and characterization of a small inhibitory peptide that can target DNA-PKcs autophosphorylation and increase tumor radiosensitivity. Int J. Radiat. Oncol. Biol. Phys..

[519] Wu YH (2017). A novel histone deacetylase inhibitor TMU-35435 enhances etoposide cytotoxicity through the proteasomal degradation of DNA-PKcs in triple-negative breast cancer. Cancer Lett..

[520] Klein C (2017). Overcoming hypoxia-induced tumor radioresistance in non-small cell lung cancer by targeting DNA-dependent protein kinase in combination with carbon ion irradiation. Radiat. Oncol..

[521] Dong W (2020). End processing factor APLF promotes NHEJ efficiency and contributes to TMZ- and ionizing radiation-resistance in glioblastoma cells. Onco Targets Ther..

[522] Zhou W (2020). Thymine DNA glycosylase-regulated TAZ promotes radioresistance by targeting nonhomologous end joining and tumor progression in esophageal cancer. Cancer Sci..

[523] Cleary JM, Aguirre AJ, Shapiro GI, D’Andrea AD (2020). Biomarker-guided development of DNA repair inhibitors. Mol. Cell.

[524] Helleday T (2008). DNA repair pathways as targets for cancer therapy. Nat. Rev. Cancer.

[525] Jin J (2020). Methylation-associated silencing of miR-193b improves the radiotherapy sensitivity of esophageal cancer cells by targeting cyclin D1 in areas with zinc deficiency. Radiother. Oncol..

[526] Ali, R. M. M., McIntosh, S. A. & Savage, K. I. Homologous recombination deficiency in breast cancer: Implications for risk, cancer development, and therapy. *Genes Chromosomes Cancer* **60**, 358–372 (2020). 10.1002/gcc.22921 33247475

[527] Belli C, Duso BA, Ferraro E, Curigliano G (2019). Homologous recombination deficiency in triple negative breast cancer. Breast.

[528] Oei AL (2017). Enhancing synthetic lethality of PARP-inhibitor and cisplatin in BRCA-proficient tumour cells with hyperthermia. Oncotarget.

[529] Parsels, L. A. et al. Combinatorial efficacy of olaparib with radiation and ATR inhibitor requires PARP1 protein in homologous recombination proficient pancreatic cancer. *Mol. Cancer Ther.* **20**, 0365 (2020). 10.1158/1535-7163.MCT-20-0365 PMC7867626 33268569

[530] Estrada-Bernal A (2015). MEK inhibitor GSK1120212-mediated radiosensitization of pancreatic cancer cells involves inhibition of DNA double-strand break repair pathways. Cell Cycle.

[531] Makita N (2015). Inhibitory effects of valproic acid in DNA double-strand break repair after irradiation in esophageal squamous carcinoma cells. Oncol. Rep..

[532] Shoji M (2012). Valproic acid, a histone deacetylase inhibitor, enhances radiosensitivity in esophageal squamous cell carcinoma. Int J. Oncol..

[533] Urick ME (2011). Enhancement of 5-fluorouracil-induced in vitro and in vivo radiosensitization with MEK inhibition. Clin. Cancer Res..

[534] Wu C (2020). Phase I Trial of trametinib with neoadjuvant chemoradiation in patients with locally advanced rectal cancer. Clin. Cancer Res..

[535] Chang L (2014). PI3K/Akt/mTOR pathway inhibitors enhance radiosensitivity in radioresistant prostate cancer cells through inducing apoptosis, reducing autophagy, suppressing NHEJ and HR repair pathways. Cell Death Dis..

[536] Goglia AG (2015). Identification of novel radiosensitizers in a high-throughput, cell-based screen for DSB repair inhibitors. Mol. Cancer Ther..

[537] Young MJ (2017). Off-target effects of drugs that disrupt human mitochondrial DNA maintenance. Front Mol. Biosci..

[538] Muresanu, C. et al. Updated understanding of cancer as a metabolic and telomere-driven disease, and proposal for complex personalized treatment, a hypothesis. *Int. J. Mol. Sci*. **21**, 6521 (2020). 10.3390/ijms21186521 PMC7555410 32906638

[539] Saini N, Gordenin DA (2018). Somatic mutation load and spectra: A record of DNA damage and repair in healthy human cells. Environ. Mol. Mutagen.

[540] Hadwiger LA, Tanaka K (2018). DNA damage and chromatin conformation changes confer nonhost resistance: a hypothesis based on effects of anti-cancer agents on plant defense responses. Front Plant Sci..

[541] Sharma A, Sharma KL, Bansal C, Kumar A (2020). Updates on "Cancer Genomics and Epigenomics". World J. Clin. Oncol..

[542] Hasanvand M (2020). Dose-response meta-analysis of arsenic exposure in drinking water and intelligence quotient. J. Environ. Health Sci. Eng..

[543] Schiewer MJ (2012). Dual roles of PARP-1 promote cancer growth and progression. Cancer Discov..

[544] Mills KD (2013). Tumor suppression: putting p53 in context. Cell Cycle.

[545] Kraljevic S (2006). Casting light on molecular events underlying anti-cancer drug treatment: what can be seen from the proteomics point of view?. Cancer Treat. Rev..

[546] Papaleo EInvestigating (2021). Conformational dynamics and allostery in the p53 DNA-binding domain using molecular simulations. Methods Mol. Biol..

[547] Sun Q (2019). Therapeutic Implications of p53 Status on Cancer Cell Fate Following Exposure to Ionizing Radiation and the DNA-PK Inhibitor M3814. Mol. Cancer Res.

[548] Lajud SA (2014). Dual disruption of DNA repair and telomere maintenance for the treatment of head and neck cancer. Clin. Cancer Res..

[549] Patnaik, A. et al. Safety and clinical activity of a new anti-PD-L1 antibody as monotherapy or combined with targeted therapy in advanced solid tumors: the PACT phase Ia/Ib trial. *Clin Cancer Res.* **27**, 1267–1277 (2020). 10.1158/1078-0432.CCR-20-2821 33229456

[550] Herbst RS (2020). Atezolizumab for first-line treatment of PD-L1-selected patients with NSCLC. N. Engl. J. Med..

[551] Powles T (2020). Avelumab maintenance therapy for advanced or metastatic urothelial carcinoma. N. Engl. J. Med..

[552] Powles T (2020). Durvalumab alone and durvalumab plus tremelimumab versus chemotherapy in previously untreated patients with unresectable, locally advanced or metastatic urothelial carcinoma (DANUBE): a randomised, open-label, multicentre, phase 3 trial. Lancet Oncol..

[553] Voltin, C. A. et al. Early response to first-line anti-PD-1 treatment in hodgkin lymphoma: a PET-based analysis from the prospective, randomized phase II NIVAHL trial. *Clin. Cancer Res.* **27**, 402–407 (2020). 10.1158/1078-0432.CCR-20-3303 33122344

[554] Rischin D (2020). PD-1 blockade in recurrent or metastatic cervical cancer: Data from cemiplimab phase I expansion cohorts and characterization of PD-L1 expression in cervical cancer. Gynecol. Oncol..

[555] Andre T (2020). Pembrolizumab in microsatellite-instability-high advanced colorectal cancer. N. Engl. J. Med..

[556] Oaknin, A. et al. Clinical activity and safety of the anti-programmed death 1 monoclonal antibody dostarlimab for patients with recurrent or advanced mismatch repair-deficient endometrial cancer: a nonrandomized phase 1 clinical trial. *JAMA Oncol*. **6**, 1766–1722 (2020). 10.1001/jamaoncol.2020.4515 PMC7530821 33001143

[557] Schouten RD (2020). Nivolumab in pre-treated advanced non-small cell lung cancer: long term follow up data from the Dutch expanded access program and routine clinical care. Transl. Lung Cancer Res..

[558] Calabro L (2013). Tremelimumab for patients with chemotherapy-resistant advanced malignant mesothelioma: an open-label, single-arm, phase 2 trial. Lancet Oncol..

[559] Kamath SD (2020). Ipilimumab and gemcitabine for advanced pancreatic cancer: a phase Ib study. Oncologist.

[560] Eikesdal, H. P. et al. Olaparib monotherapy as primary treatment in unselected triple negative breast cancer. *Ann. Oncol.*10.1016/j.annonc.2020.11.009 (2020). 10.1016/j.annonc.2020.11.009 33242536

[561] Tampaki EC (2018). Efficacy and safety of neoadjuvant treatment with bevacizumab, liposomal doxorubicin, cyclophosphamide and paclitaxel combination in locally/regionally advanced, HER2-negative, grade III at premenopausal status breast cancer: a phase II study. Clin. Drug Investig..

[562] Leone JP (2020). Phase II trial of carboplatin and bevacizumab in patients with breast cancer brain metastases. Breast Cancer Res..

[563] Litton JK (2020). Talazoparib versus chemotherapy in patients with germline BRCA1/2-mutated HER2-negative advanced breast cancer: final overall survival results from the EMBRACA trial. Ann. Oncol..

[564] Dieras V (2020). Veliparib with carboplatin and paclitaxel in BRCA-mutated advanced breast cancer (BROCADE3): a randomised, double-blind, placebo-controlled, phase 3 trial. Lancet Oncol..

[565] Vinayak, S. et al. Open-label clinical trial of niraparib combined with pembrolizumab for treatment of advanced or metastatic triple-negative breast cancer. *JAMA Oncol.* **5**, 1132–1140 (2019). 10.1001/jamaoncol.2019.1029 PMC6567845 31194225

[566] O’Shaughnessy J (2011). Iniparib plus chemotherapy in metastatic triple-negative breast cancer. N. Engl. J. Med..

[567] Riches LC (2020). Pharmacology of the ATM Inhibitor AZD0156: potentiation of irradiation and olaparib responses preclinically. Mol. Cancer Ther..

[568] Gatti-Mays ME (2020). A phase II single arm pilot study of the CHK1 inhibitor prexasertib (LY2606368) in BRCA wild-type, advanced triple-negative breast cancer. Oncologist.

[569] Oza AM (2020). Patient-centered outcomes in ARIEL3, a Phase III, randomized, placebo-controlled trial of rucaparib maintenance treatment in patients with recurrent ovarian carcinoma. J. Clin. Oncol..

[570] Mirza MR (2020). Long-term safety in patients with recurrent ovarian cancer treated with niraparib versus placebo: Results from the phase III ENGOT-OV16/NOVA trial. Gynecol. Oncol..

[571] Zsiros E (2021). Efficacy and safety of pembrolizumab in combination with bevacizumab and oral metronomic cyclophosphamide in the treatment of recurrent ovarian cancer: a phase 2 nonrandomized clinical trial. JAMA Oncol..

[572] Friedlander, M. et al. Patient-centred outcomes and effect of disease progression on health status in patients with newly diagnosed advanced ovarian cancer and a BRCA mutation receiving maintenance olaparib or placebo (SOLO1): a randomised, phase 3 trial. *Lancet Oncol*. 10.1016/S1470-2045(21)00098-X (2021). 10.1016/S1470-2045(21)00098-X 33862001

[573] De Simone, M. et al. Pressurized intraperitoneal aerosol chemotherapy (PIPAC) with oxaliplatin, cisplatin, and doxorubicin in patients with peritoneal carcinomatosis: an open-label, single-arm, phase ii clinical trial. *Biomedicines*. **8**, 102 (2020). 10.3390/biomedicines8050102 PMC7277495 32365877

[574] Iyer G (2018). Multicenter prospective phase II trial of neoadjuvant dose-dense gemcitabine plus cisplatin in patients with muscle-invasive bladder cancer. J. Clin. Oncol..

[575] Colombo R (2012). Neoadjuvant short-term intensive intravesical mitomycin C regimen compared with weekly schedule for low-grade recurrent non-muscle-invasive bladder cancer: preliminary results of a randomised phase 2 study. Eur. Urol..

[576] Choueiri TK (2014). Neoadjuvant dose-dense methotrexate, vinblastine, doxorubicin, and cisplatin with pegfilgrastim support in muscle-invasive urothelial cancer: pathologic, radiologic, and biomarker correlates. J. Clin. Oncol..

[577] Zhang T (2015). The ATM inhibitor KU55933 sensitizes radioresistant bladder cancer cells with DAB2IP gene defect. Int J. Radiat. Biol..

[578] Segovia C (2019). Inhibition of a G9a/DNMT network triggers immune-mediated bladder cancer regression. Nat. Med..

[579] Munemoto Y (2018). A phase II trial to evaluate the efficacy of panitumumab combined with fluorouracil-based chemotherapy for metastatic colorectal cancer: the PF trial. Cancer Chemother. Pharmacol..

[580] Kim SY (2009). A phase II study of S-1 plus irinotecan and oxaliplatin in heavily-treated patients with metastatic colorectal cancer. Invest N. Drugs.

[581] Ando K (2020). Efficacy and feasibility of S-1 plus oxaliplatin (C-SOX) for treating patients with stage III colon cancer (KSCC1303): final analysis of 3-year disease-free survival. Int J. Clin. Oncol..

[582] Chen EX (2016). A Phase I study of olaparib and irinotecan in patients with colorectal cancer: Canadian Cancer Trials Group IND 187. Invest N. Drugs.

[583] Gallia GL (2021). Mebendazole and temozolomide in patients with newly diagnosed high-grade gliomas: results of a phase 1 clinical trial. Neurooncol Adv..

[584] Wang Y (2020). A randomized, double-blind, placebo-controlled study of B-cell lymphoma 2 homology 3 mimetic gossypol combined with docetaxel and cisplatin for advanced non-small cell lung cancer with high expression of apurinic/apyrimidinic endonuclease 1. Invest N. Drugs.

[585] Provencio M (2021). Phase II clinical trial with metronomic oral vinorelbine and tri-weekly cisplatin as induction therapy, subsequently concomitant with radiotherapy (RT) in patients with locally advanced, unresectable, non-small cell lung cancer (NSCLC). Analysis of survival and value of ctDNA for patient selection. Lung Cancer.

[586] Owonikoko, T. K. et al. Phase 2 study of talazoparib in patients with homologous recombination repair-deficient squamous cell lung cancer: Lung-MAP substudy S1400G. *Clin. Lung Cancer* **10***,* S1525-7304(21)00004-8(2021). 10.1016/j.cllc.2021.01.001 PMC8637652 33583720

[587] Farago AF (2019). Combination olaparib and temozolomide in relapsed small-cell. Lung Cancer Cancer Discov..

[588] Royer B (2016). Bortezomib, doxorubicin, cyclophosphamide, dexamethasone induction followed by stem cell transplantation for primary plasma cell leukemia: a prospective phase ii study of the intergroupe francophone du myelome. J. Clin. Oncol..

[589] van Tilburg CM (2019). Phase I/II intra-patient dose escalation study of vorinostat in children with relapsed solid tumor, lymphoma, or leukemia. Clin. Epigenetics..

[590] Li X (2018). High PARP-1 expression predicts poor survival in acute myeloid leukemia and PARP-1 inhibitor and SAHA-bendamustine hybrid inhibitor combination treatment synergistically enhances anti-tumor effects. EBioMed..

[591] Ghelli Luserna Di Rora A (2016). Prexasertib, a Chk1/Chk2 inhibitor, increases the effectiveness of conventional therapy in B-/T- cell progenitor acute lymphoblastic leukemia. Oncotarget.

[592] Ikeda M (2017). A Phase I/II trial of continuous hepatic intra-arterial infusion of 5-fluorouracil, mitoxantrone and cisplatin for advanced hepatocellular carcinoma. Jpn J. Clin. Oncol..

[593] El Dika I (2020). Phase II trial of sorafenib and doxorubicin in patients with advanced hepatocellular carcinoma after disease progression on sorafenib. Cancer Med.

[594] Bridgewater JA (2020). Systemic chemotherapy with or without cetuximab in patients with resectable colorectal liver metastasis (New EPOC): long-term results of a multicentre, randomised, controlled, phase 3 trial. Lancet Oncol..

[595] Correction to Lancet Oncol 2019; 20: 663-73. *Lancet Oncol*. **20**, e242, (2019). 10.1016/S1470-2045(19)30216-5 30952558

[596] Gabrielson A (2015). Phase II study of temozolomide and veliparib combination therapy for sorafenib-refractory advanced hepatocellular carcinoma. Cancer Chemother. Pharmacol..

[597] Niesvizky R (2015). Phase 1/2 study of cyclin-dependent kinase (CDK)4/6 inhibitor palbociclib (PD-0332991) with bortezomib and dexamethasone in relapsed/refractory multiple myeloma. Leuk. Lymphoma.

[598] Khashab T (2019). Long-term overall- and progression-free survival after pentostatin, cyclophosphamide and rituximab therapy for indolent non-Hodgkin lymphoma. Br. J. Haematol..

[599] Cohen JB (2015). A phase II study of bortezomib added to rituximab, cyclophosphamide, doxorubicin, vincristine, and prednisone in patients with previously untreated indolent non-Hodgkin’s lymphoma. Br. J. Haematol..

[600] Schaefer NG, James E, Wahl RL (2011). Poly(ADP-ribose) polymerase inhibitors combined with external beam and radioimmunotherapy to treat aggressive lymphoma. Nucl. Med Commun..

[601] Flaig TW (2006). A phase II trial of dexamethasone, vitamin D, and carboplatin in patients with hormone-refractory prostate cancer. Cancer.

[602] Buonerba C (2011). Phase II trial of cisplatin plus prednisone in docetaxel-refractory castration-resistant prostate cancer patients. Cancer Chemother. Pharmacol..

[603] Pishvaian MJ (2020). A Phase I/II Study of Veliparib (ABT-888) in Combination with 5-Fluorouracil and Oxaliplatin in Patients with Metastatic Pancreatic Cancer. Clin. Cancer Res..

[604] Okano N (2020). Phase II clinical trial of gemcitabine plus oxaliplatin in patients with metastatic pancreatic adenocarcinoma with a family history of pancreatic/breast/ovarian/prostate cancer or personal history of breast/ovarian/prostate cancer (FABRIC study). Int J. Clin. Oncol..

[605] Wang-Gillam A (2019). NAPOLI-1 phase 3 study of liposomal irinotecan in metastatic pancreatic cancer: Final overall survival analysis and characteristics of long-term survivors. Eur. J. Cancer.

[606] Bendell J (2015). Phase I study of olaparib plus gemcitabine in patients with advanced solid tumours and comparison with gemcitabine alone in patients with locally advanced/metastatic pancreatic cancer. Ann. Oncol..

